# Perturbation Biology: Inferring Signaling Networks in Cellular Systems

**DOI:** 10.1371/journal.pcbi.1003290

**Published:** 2013-12-19

**Authors:** Evan J. Molinelli, Anil Korkut, Weiqing Wang, Martin L. Miller, Nicholas P. Gauthier, Xiaohong Jing, Poorvi Kaushik, Qin He, Gordon Mills, David B. Solit, Christine A. Pratilas, Martin Weigt, Alfredo Braunstein, Andrea Pagnani, Riccardo Zecchina, Chris Sander

**Affiliations:** 1 Computational Biology Program, Memorial Sloan-Kettering Cancer Center, New York, New York, United States of America; 2 Tri-Institutional Program for Computational Biology and Medicine, Weill Cornell Medical College, New York, New York, United States of America; 3 Department of Systems Biology, The University of Texas MD Anderson Cancer Center, Houston, Texas, United States of America; 4 Program in Molecular Pharmacology, Memorial Sloan-Kettering Cancer Center, New York, New York, United States of America; 5 Human Oncology and Pathogenesis Program, Memorial Sloan-Kettering Cancer Center, New York, New York, United States of America; 6 Department of Pediatrics, Memorial Sloan-Kettering Cancer Center, New York, New York, United States of America; 7 Laboratoire de Génomique des Microorganismes, Université Pierre et Marie Curie, Paris, France; 8 Politecnico di Torino and Human Genetics Foundation, HuGeF, Torino, Italy; Cancer Research UK Cambridge Research Institute, United Kingdom

## Abstract

We present a powerful experimental-computational technology for inferring network models that predict the response of cells to perturbations, and that may be useful in the design of combinatorial therapy against cancer. The experiments are systematic series of perturbations of cancer cell lines by targeted drugs, singly or in combination. The response to perturbation is quantified in terms of relative changes in the measured levels of proteins, phospho-proteins and cellular phenotypes such as viability. Computational network models are derived *de novo*, i.e., without prior knowledge of signaling pathways, and are based on simple non-linear differential equations. The prohibitively large solution space of all possible network models is explored efficiently using a probabilistic algorithm, Belief Propagation (BP), which is three orders of magnitude faster than standard Monte Carlo methods. Explicit executable models are derived for a set of perturbation experiments in SKMEL-133 melanoma cell lines, which are resistant to the therapeutically important inhibitor of RAF kinase. The resulting network models reproduce and extend known pathway biology. They empower potential discoveries of new molecular interactions and predict efficacious novel drug perturbations, such as the inhibition of PLK1, which is verified experimentally. This technology is suitable for application to larger systems in diverse areas of molecular biology.

## Author Summary

Drugs that target specific effects of signaling proteins are promising agents for treating cancer. One of the many obstacles facing optimal drug design is inadequate quantitative understanding of the coordinated interactions between signaling proteins. *De novo* model inference of network or pathway models refers to the algorithmic construction of mathematical predictive models from experimental data without dependence on prior knowledge. *De novo* inference is difficult because of the prohibitively large number of possible sets of interactions that may or may not be consistent with observations. Our new method overcomes this difficulty by adapting a method from statistical physics, called Belief Propagation, which first calculates probabilistically the most likely interactions in the vast space of all possible solutions, then derives a set of individual, highly probable solutions in the form of executable models. In this paper, we test this method on artificial data and then apply it to model signaling pathways in a BRAF-mutant melanoma cancer cell line based on a large set of rich output measurements from a systematic set of perturbation experiments using drug combinations. Our results are in agreement with established biological knowledge, predict novel interactions, and predict efficacious drug targets that are specific to the experimental cell line and potentially to related tumors. The method has the potential, with sufficient systematic perturbation data, to model, *de novo* and quantitatively, the effects of hundreds of proteins on cellular responses, on a scale that is currently unreachable in diverse areas of cell biology. In a disease context, the method is applicable to the computational design of novel combination drug treatments.

## Introduction

### Signaling in cancer cells

Abnormal biomolecular information flow as a result of genetic or epigenetic alterations may lead to tumorigenic transformation and malignancy and is classically modeled as changes in signaling pathways [1]. Targeted anti-cancer drugs, which bind and inhibit specific components of aberrant signaling pathways, are a promising alternative to conventional chemotherapy, with recent successes in melanoma (RAF inhibitor) [2] and prostate cancer (AR inhibitor) [3], [4] following in the footsteps of the pioneering BCR-ABL inhibitor Imatinib [5] and EGFR inhibitors Gefitinib and Erlotinib [6], [7], [8]. Combinations of targeted anticancer drugs hold considerable promise because of the emergence of resistance to initially successful single agents and the highly robust nature of the signaling pathways with multiple feedback mechanisms [9].

### Data-driven models of cell biology

High throughput measurements on response profiles of living cells to multiple perturbations such as drug combinations provide a rich set of information to construct quantitative cell biology models. In this paper, we construct context specific *de novo* mathematical models of signaling pathways through the use of systematic paired perturbation experiments and network inference algorithms. Such network models provide insight into mechanistic details of signaling pathways, predict the response of cellular systems to multiple perturbations beyond those from which models are derived, and guide the design of perturbations for a desired response.

### State of the art in network inference in cell biology

Previous mathematical models of molecular signaling in cells have been effective in modeling pathways and enhancing drug discovery [10], [11], [12], [13], [14], [15], [16], [17], [18], [19]. Techniques for network modeling of signaling pathways span a wide spectrum of complexity. Detailed chemical kinetics and spatiotemporal models [17], [20], [21] can provide mechanistic explanations of observed behavior, but are often incompletely parameterized, needing tens or hundreds of sensitive parameters for medium-sized systems. Moreover, such models may not be valid in biological contexts that differ substantially from dilute solution chemistry. On the other end of the spectrum, pattern matching or machine learning models such as neural networks and correlation-based models such as maximum entropy [22] can accurately provide purely data-driven models of signaling. However, such methods have limited power to explain mechanistic details and, in most cases, are insufficient for quantitative predictions of system behavior in conditions beyond those from which the models are derived.

### Data-driven and context-specific predictive models

**Figure 1:**
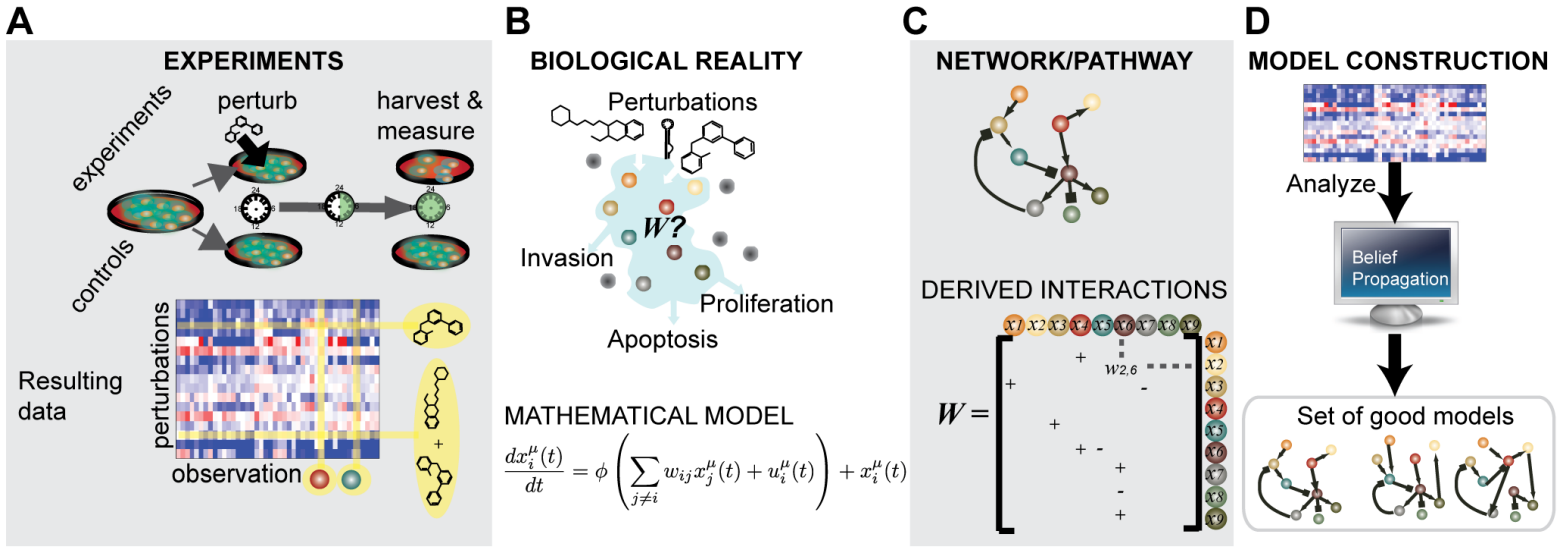
Perturbation cell biology. Perturbing cancer cells with targeted drugs singly and in pairs (A) reveals context-specific response to therapies and illuminates protein interactions. We construct dynamic mathematical models of the cells' response to drugs that have both quantitative parameters (B) and a qualitative network interpretation (C). We use an inference algorithm called Belief Propagation (BP) to construct a set of good, i.e., predictive models (D).

We take a unique modeling approach to construct context specific, *de novo* and predictive network models of signaling pathways from drug perturbation data (Figure 1). Here, *de novo* means that network inference is done without depending on known molecular interactions extracted from literature or pathway databases, which do not account for biological context. This approach also emphasizes context specificity since it relies on rich experimental data from a single biological context as its training set. The models are constructed through parameterization of a simple model equation, which has been used in other network modeling approaches [13], [23], [24], [25], [26], [27]. The model equations contain parameters that are mechanistically descriptive of direct or indirect interactions in the system. Finally, the models are computationally predictive of cell-type specific response to new drug perturbations and their combinations. We expect that this conceptual framework and the technical advances in network inference will empower the community to identify unique drug targets and combinations that are particularly efficacious within specific disease contexts.

### Network modeling *de novo* or with prior information

*De novo* construction of signaling network models at scales relevant to problems related to complex biological phenomena such as cancer has long been a challenge in system biology. Thus, quantitative models of protein signaling pathways are typically constructed on the basis of existing prior knowledge from literature searches [16], [17] and interaction databases [19], [28], [29]. However, different cancer contexts have unique genetic and proteomic alterations to normal protein signaling. For example, distinct mutations in effector proteins of the PI3K pathway are oncogenic in unique ways, i.e., they lead to distinctly context dependent functional consequences in different cancer types, subtypes and patients [30]. The method we introduce here is capable of inferring parameterized network models with prior knowledge, yet the results in this study do not include prior knowledge. An advantage of *de novo* inference is independence from prior knowledge interactions that may be incorrect or incomplete in a particular biological context.

### Model inference from perturbation data for larger systems is hard

The largest obstacle to *de novo* model construction is the combinatorial explosion in the number of possible network models, which defines the solution space [31]. Model inference problems of this type are NP-hard [32]. The number of possible configurations for a model with N nodes, and K possible values for each parameter grows super-exponentially as 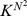. We have previously described a method named CoPIA for *de novo* construction of dynamic nonlinear network models from perturbation data [33]. CoPIA is based on the combined use of a Monte Carlo stochastic search algorithm, which is used to search the network configurations and an efficient gradient descent algorithm [34] for quantitative parameter optimization. However, without algorithmic improvements, such Monte Carlo based methods are limited to modeling fewer than approximately 15–20 biological entities [33]. Increasing the scale of network (or pathway) models of cellular signaling processes to levels sufficient to describe complex biological problems in quantitative detail is therefore extremely challenging and has been approached with a diversity of methods [35].

### A statistical physics approach can handle the complexity for larger systems

An ingenious, two-step approach to deal with network inference in larger systems is based on first calculating probability distributions for each possible interaction in the model and then computing distinct solutions by sampling these probability distributions. For this purpose, we employ a probability model of network configurations inspired from statistical physics principles. Following a set of approximations to simplify the probability model, we apply a custom adaptation of an iterative algorithm called Belief Propagation (BP). BP involves local optimization updates to probability distributions of individual model parameters that converge to a stable set of probability distributions, which collectively describe a set of good network model solutions [36], [37], [38]. BP has been applied to various complex inference problems, some of them NP-Hard such as K-SAT [38] and graph coloring [39]. BP has garnered some attention in biological network inference [40], [41] and parameter estimation [42], [43]. Here, we tailor the BP algorithm to large-scale perturbation data that is capable of increasing the scope of the models to hundreds of nodes. The result of BP is a set of probability distributions for each model parameter, often referred to as marginal probability distributions, or ‘marginals’. Each marginal describes the inferred distribution of a particular parameter across a range of high probability solutions. Individual models are created via sampling from these marginals [44]. Consequently, the time-complexity of the problem is strongly reduced, the prohibitive cost from combinatorial complexity is circumvented and, although the method provides only an approximate solution, one obtains useful, non-trivial results.

### In practice: from systematic perturbation to response profiling to network model inference

Our algorithmic network pharmacology approach involves four major steps: (i) perturbation experiments with combinations of targeted compounds; (ii) high-throughput quantitative measurements of proteomic changes (e.g., reverse phase protein arrays or mass spectrometry) and phenotypic changes (e.g., cell viability or apoptosis); (iii) inference of quantitative network models of protein signaling that explain and link these changes; and (iv) use of the network models to predict cellular and molecular responses to diverse perturbations, beyond the conditions on which the network models are derived.

### Experimental and computational technology for network model inference and application to drug effects on melanoma cell lines

In this work, we adapt BP to construct quantitative network models of signaling pathways from systematic perturbation experiments. We evaluate the speed and accuracy of BP on toy data generated from biologically inspired network structures. The inference on this toy data reveals that BP offers a significant improvement in computational efficiency compared to traditional Monte Carlo simulations without a sacrifice in accuracy. Furthermore, we construct network models of signaling in a RAF inhibitor resistant melanoma cell line (SKMEL-133), which has the BRAFV600E mutation [45], [46]. The models are predictive of both the proteomic and phenotypic response to drug combinations. Model simulations successfully predict the phenotypic response profiles of SKMEL-133 cells to novel drug targets. With the introduction of many novel targeted drugs and patient specific genomic profiling, this network pharmacology approach aims to provide an effective tool to develop individualized combination therapies against multiple cancer forms.

## Results

### Theory

#### Mathematical framework of the network model

Key decisions in modeling a biological cellular system include the choice of variables and the mathematical framework for representing system dynamics. Here, we work with a fairly simple but powerful *ansatz* or framework, in which the time behavior of the cellular system {*x_i_(t)*} in a set of perturbation conditions {*u_i_^μ^*} is modeled as a series of coupled non-linear differential equations (Equation 1) [33].

*Equation 1: Non-linear network model for the time behavior of the cellular system*

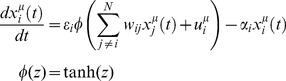
(1)


The system variables *x* represent quantities of particular biological entities one wishes to measure and model. In this work, quantities are restricted to relative changes in protein and phospho-protein abundances and cell viability levels. The variables are nodes in the network model. The model parameters *w* in the matrix ***W*** formally quantify the interactions between nodes and correspond to directed edges in the network model. Equation 1 includes an independent time variable *t*, which denotes that variables are functions of time. The term *u* represents an external force on a model variable, which models interventions from targeted drug perturbations. A vector of *u*-values defines the set of targeted perturbations; combinations are simple additions of these vectors. In principle, *u* is time-dependent, but not in the current implementation. The variable index *i* maps to a single network node, and the experimental index (*μ*) maps to a single experimental perturbation condition. A biological system is therefore modeled by a collection of coupled equations of the form defined in Equation 1. Perturbations to any node propagate in time through the network interactions producing trajectories *x(t)*, which present the behavior of the system over time.

Theoretically, the model variables can quantify any measure of interest. While absolute protein concentrations are one option, such data is difficult to acquire in high throughput assays. In this study, we focus on log_2_-ratios of abundances in perturbed conditions to abundances in the unperturbed condition. Consequently, model variables can take both negative and positive values, which denote decreased or increased quantities of the corresponding biological entity. We choose to normalize all measurement levels against unperturbed levels in order to focus on signaling differences due directly to perturbation.

The rate of change of any variable, in this formulation, is predominantly influenced by the additive linear combination of upstream nodes {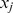} weighted by their respective interaction strength {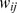}. Only non-zero values of 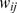 are interactions in the network model. We incorporate nonlinearity with a sigmoidal function 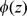 that limits both the maximum positive and negative rates of change [24], controlled by the parameter 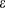. The 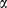 parameter models the rate of restoration at which a model variable would return to its initial value before perturbation, in the absence of interactions. This is analogous to the degradation rate in models of positively valued protein concentrations. The parameters 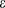 and 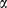 are not inferred with BP. For the remainder of this section they are assumed to be 1, and are dropped from the equations. They are reintroduced in the final stage of modeling, when individual models are optimized with gradient descent.

The network models are parameterized by the square interaction matrix ***W*** = {*w_ij_*} of size N (N*^2^* entries), where 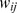 represents a directed interaction between nodes, quantifying the influence of *x_j_* on the rate of change of *x_i_*. In chemical kinetics, the *w_ij_* is analogous to rate constants in units of inverse time, although no explicit rates are derived here. Equation 1 describes the dynamic behavior of the system, given a constant interaction matrix ***W***. In this work, we explicitly forbid self-interactions, therefore the N diagonal entries of ***W*** are set to 0 and only the remaining N*^2^-*N are subject to fitting. These apparently simple models can represent biologically realistic regulatory motifs, such as serial and parallel pathway connectivity, positive and negative feedback loops and feed-forward control. The models used here are dynamic in the sense that they can be simulated as temporal trajectories that converge to a steady state. In this work, the parameters are inferred based solely on data assumed to represent the biological steady state. Thus, only the endpoints of the simulated trajectories are constrained. Despite not being used in this study, both the model and the learning method can generalize to incorporate time-series data.

#### The problem of model inference

The problem of deriving useful models of a (biological) system is called ‘model inference’. The objective of model inference, given a mathematical framework like that described above, is to find a set of parameters such that the model equations best reproduce a training set of experimental data and have predictive power beyond the training set. In the present modeling framework, we aim to find numerical values for the N*^2^–*N free parameters in the interaction matrix ***W***, such that descriptive and predictive power of the model is optimized. Genuine predictive power, rather than just descriptive power, requires both low error and low complexity of the model, combined as low *cost*. We quantify the cost of ***W*** by an objective cost function *C(* ***W*** *)* that penalizes: (i) discrepancies between predicted 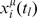 and experimentally measured 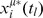 values of the system observables at a set of time points *{t_l_}* in condition *μ*; and (ii) the number of non-zero interactions in ***W***. Lower cost models tend to have more predictive power than higher cost models.

*Equation 2* *: Model configuration cost function*

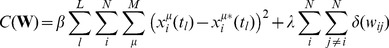
(2.a)

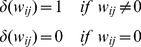
(2.b)


*C(* ***W*** *)* is thus the error-plus-complexity cost of a parameter configuration ***W***. The cost components are weighted by 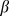 and 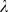, respectively. The complexity cost term is an *L0* penalty [47], [48], [49], [50], [51] that penalizes non-zero entries in ***W*** and is included to both avoid overfitting and reflect the empirical observation that realistic biological networks such as gene regulatory networks or protein-protein interaction networks are sparse [52]. While *L1* penalties are convex and therefore amenable to efficient convex optimization methods, they are not used here since they provide weaker constraint on the complexity of the interaction matrix ***W*** [25], [53], [54], [55]. We do not wish to include direct self-interaction in the present version; thus, the sum in the complexity term does not include the diagonal elements 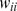. The computational challenge of network inference is to translate the information contained in a set of experimental observations into an optimal set of models, as represented by a set of low cost interaction matrices ***W***. In this report, we work in the steady-state approximation and thus ignore the time variable in the cost function in Equation 2.

#### De novo network inference is a hard problem

In principle, to infer optimal network configurations one has to compute the cost of all possible network configurations. However, explicit enumeration and cost calculation of all possible parameter configurations ***W*** is a prohibitively complicated task for even moderately sized systems. To estimate the complexity of this task, assume that any *w_ij_* can take on K discrete values out of a value range *Ω*, for example, 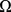 = {−1,0,+1} for K = 3, representing inhibition, no interaction and activation, respectively. As the number of model nodes N increases, the number of possible parameter configurations increases as 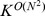. Even for moderate N, e.g., 20 nodes, the number of distinct configurations is of order 10^190^, obviously a very large number, making explicit enumeration prohibitive.

The solution space refers to the set of all possible model configurations. A reasonably clever strategy to traverse this enormous solution space is guided random exploration, e.g., by a traditional Monte Carlo search, in which random moves in multi-dimensional parameter space are kept or rejected based on the cost of the resulting configuration, with a non-zero but small probability of accepting higher cost configurations in order to facilitate the escape from local minima. In an earlier study, we successfully used a Monte Carlo search followed by a modified gradient descent method to derive a set of low cost models for a relatively small system. This earlier algorithm achieved a reasonable exploration of solution space for a system of 14 variables, as assessed by the recurrence of dominant interactions across the set of a few hundred low-cost models and the agreement of those interactions with well-established knowledge of signaling pathways in cell biology. However, the 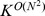
argument above and explicit computational benchmarking indicate that such Monte Carlo searches become prohibitively expensive for larger systems.

#### Fast inference via a probability model of network configurations

In search of a more efficient algorithm, we adopt an idea originally developed in statistical physics, and widely used in solving complicated optimization problems in computer science and other areas. Instead of sampling a prohibitively large, unrestricted solution space by traversing a set of individual configurations, the idea is to first calculate high probability regions and then restrict exploration to this smaller solution space. In particular, we describe high probability regions by calculating probability distributions of individual model parameters over possible value assignments. Then, we can generate distinct model configurations by sampling from the calculated probability distributions.

Models with a large error (or cost) have low probability, while those with a low error have high probability. More precisely, the probability of any particular model can be computed from its cost, which depends on the parameters in the interaction matrix ***W*** and the experimental data (Equation 2). In statistical physics, there is an analogous relationship between the Hamiltonian for the states of a system and its Boltzmann-Gibbs probability distribution over all states. In terms of Bayesian inference, the equation below relates the posterior distribution of the model on the left to the likelihood function and prior distributions on the right.

*Equation 3: The probability model of network configurations*

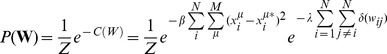



The variable Z is the partition function, which ensures that the sum of the probabilities over all model configurations is equal to one. In the statistical physics analogy, the exponents contain interaction energies and the parameter β is an inverse temperature (1/T), such that higher values of 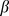 assign higher probability to lower cost configurations. The parameter λ is the weight of the complexity penalty. The choice of 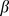 and 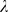 is non-trivial and is an open area of research (see Methods). Given the probabilistic model in Equation 3, the practical challenge is to identify configurations of model parameters in ***W*** that represent maximally probable models, given the data. The explicit computation of probabilities for all possible sets of parameters is not feasible even for moderately sized (N>15) systems. One therefore has to invent practical algorithms for effectively exploring the total solution space and approximately determining sets of good models.

#### Iterative optimization of the probability model

An effective solution is to use an iterative algorithm to approximate the probability distributions of the individual parameters by themselves, often called marginal probability distributions, or simply ‘marginals’. From these marginals, we can describe high probability model configurations for the full system. This iterative algorithm begins with a set of random marginals. In each iteration step, one assumes approximate knowledge of all parameter marginals (‘global information’) and then performs optimization updates on an individual marginal (‘local update’). The local update takes immediate effect and becomes part of the ‘global information’ for successive iterations as the algorithm traverses over all marginals for individual updating. The iteration terminates when it converges to a stable set of marginals. The nature of any local update to a single parameter (e.g., a node-node interaction parameter) is a calculation optimizing a balance of fitness to experimental data and consistency with the global information. The iterative application of this ‘global to local and back’ optimization strategy results in marginals for all system parameters given a probabilistic model. Such optimal marginals are informative by themselves, but are also useful for constructing a population of explicit individual high probability model configurations, which are useful for model simulation studies.

This type of probabilistic method originates in statistical physics and has been generalized to a number of hard optimization problems in statistical physics and computer science. An early application of such probabilistic inference was inverse parameter inference for disordered diluted spin systems [56], [57], [58]. A well known formulation in terms of Bayesian statistics led to the term ‘belief propagation’ [59] (BP).

**Figure 2:**
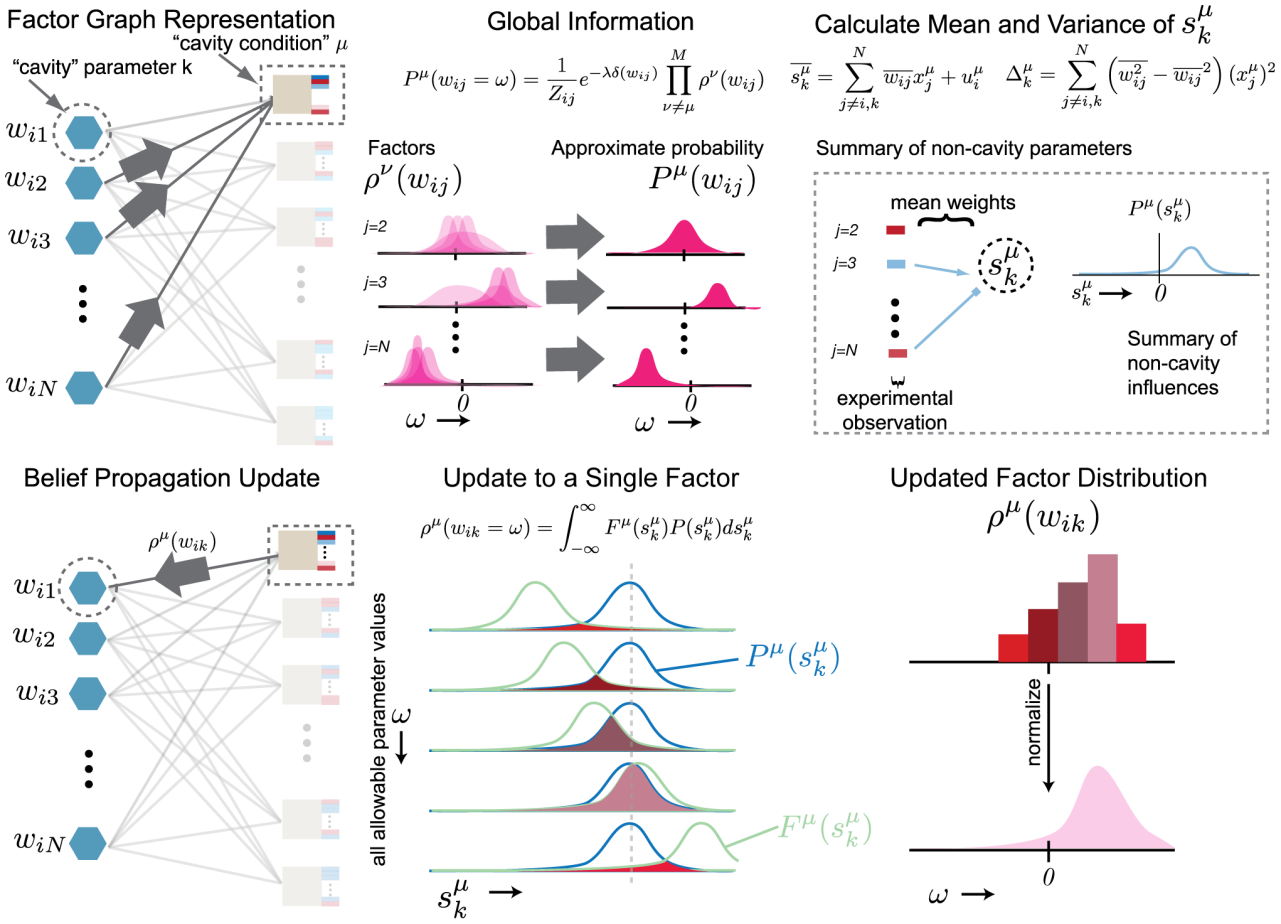
Iteration process for Belief Propagation. Top panel: the global information consists of collecting the probability distributions of the non-cavity parameters without the contribution from the cavity condition. This is a simple product over all 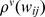 factors except that from the cavity constraint *μ*. Distributions centered on zero denote unlikely interactions (see *j = 2*), centered on the right of zero denote likely positive interactions (see *j = 3*), and centered on the left denote likely negative interactions (see *j = N*). These distributions inform the parameters of the Gaussian distribution for the mean-field, aggregate sum variable 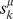. The distribution 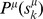 summarizes the state of the non-cavity parameters. Bottom panel: we calculate the probability of each possible parameter assignment 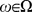 to the cavity parameter *w_ik_* constrained to the data in the cavity condition. This calculation boils down to a simple convolution of the fitness function with a fixed parameter assignment 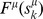 with the probability of the aggregate sum variable 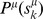, obtained by integrating over all values of 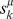. Each assignment 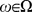 contributes proportional to the area under the curve. The resulting update is the contribution of condition *μ* on the distribution of 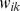, denoted 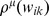. This recently updated distribution becomes part of the global information for successive updates to other parameters.

The BP approach, also known as the Bethe-Peierls approximation or cavity method in statistical physics, provides an approximate method for computing marginal probability distributions on a class of probabilistic graphical models called factor graphs. In general, a joint probability distribution over many variables may factorize into a product of factors. A factor in a factor graph represents an independent contribution to the joint probability distribution, and is connected to the variables that depend on that factor. Typically, a factor defines a constraint on a subset of variables. The BP method is proven to be exact on tree-shaped factor graphs. It has many useful applications in approximating distributions on sparse factor-graphs [60], [61] where the influence of loops in the factor graph is expected to be weak. More recently, several applications to dense, loopy factor-graphs have been proposed [40], [62], [63], [64], [65]. The problem we address here is a dense factor graph, where each factor is connected to N variables, which in this framework are the model parameters in Equation 1 (Figure 2).

A major advantage of BP algorithms is the reduction of computational complexity. This not only leads to a substantial reduction in computational effort for smaller systems but also opens the door to solving inference problems for larger systems, which would otherwise be prohibitive.

#### Simplified probability model of network configurations

We use a series of assumptions, described below, to factorize the probability model in Equation 3 into a form that can be efficiently calculated without sacrificing the quantitative and predictive nature of the models. The assumptions below reduce the problem from a probability distribution over whole model configurations (Equation 3) into a collection of marginal probability distributions for each individual parameter. Subsequent sampling from these individual marginals will result in efficient exploration of high probability model configurations.

#### Assumption 1: Discrete set of real valued parameter assignments

To simplify the probability model, we compute the probability distributions for model parameters over discrete values, from a set Ω, rather than for continuous values. The choice of discretization is an important detail and affects the convergence properties of the BP algorithm and the quality of the resulting marginals. Empirically, with the data set at hand we find that a set of 11 discrete values, centered at zero, rarely fails to converge to a stable set of marginals. Conversely, searching over only 3 weight values results in a high rate of non-convergence. As for the quality of the resulting marginals, the entropies are close to zero if we limit the search to only 3 discrete values (see Supplemental Text S1). The entropy is a statistical measure of the uncertainty in distributions, such that zero entropy distributions imply absolute certainty, i.e., each parameter is predicted to be 100% zero or 100% non-zero. This is an undesirable quality; one wants the BP marginals to constrain a set of highly probable solutions, not return one model configuration. In practice, discretizing with 11 weights gives intuitively reasonable marginals and balances the restriction and exploration of solution space. In the final stage of network inference strategy, we refine the set of discrete valued model parameters to solutions with continuous model parameters using a local gradient descent optimization algorithm [33].

#### Assumption 2: Decoupling of variables at steady state

In the dynamic model, the system variables are coupled such that a change to any variable propagates to all others via the time derivatives as in Equation 1. A rigorous way to compute the fitness of a configuration is to simulate a configuration and then compare simulation output to the training data. Such a computation, while feasible in principle, is very costly. An alternative is to take advantage of the relationship at the steady state (Equation 4) where the time-derivative is equal to zero.

*Equation 4: Model equation at steady state*

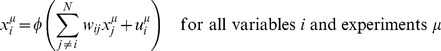



Equation 4 is a system of self-consistent equations for all variables {*x_i_*}. To avoid having to do numerical simulation, we replace {*x_j_^μ^*} on the right hand side of Equation 4 with experimentally observed {x_j_^μ*^} at the expense of self-consistency (Equation 5).

*Equation 5: Approximate model equation at steady-state*

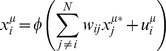



This approximation decouples the variables from each other: the model predicted value of 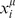 depends only on the parameters in the *i* ^th^ row of the interaction weight matrix ***W*** and on the set **{** x_j_^μ*^ **}** of experimentally measured values in condition *μ*. Thus, the posterior probability *P(* ***W*** *)* can be factorized as a product of independent posterior probability distributions over configurations of individual rows of the weight matrix. Consequently, we have an independent probability distribution for each row of the interaction weight matrix 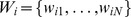. These rows describe interactions from variables 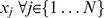 to the single variable 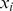. The resulting factorized expressions are:

*Equation 6a* *–* *d* *: Probability model of configurations with decoupled nodes*

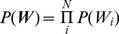
(6.a)

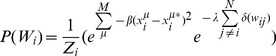
(6.b)

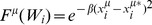
(6.c)

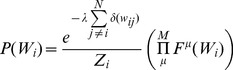
(6.d)


We introduce the notation 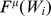 to denote the fitness of the model configuration *W_i_* to the data from experimental μ. It is important to note that the posterior probability distribution in Equation 6d factorizes over the fitness functions, such that 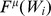 contributes independently to the full probability distribution of that configuration. The probability distribution in 6d, however, does not factorize any further, since each parameter *w_ik_* in *W_i_* depends on the other parameters in *W_i_*. In order to reach good solutions for 6d without enumerating all configurations *W_i_*, we apply an iterative method to infer marginals for the constituent parameters {*w_ij_* 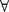 *j*}.

#### Belief Propagation algorithm: Iterative updates of probability estimates

As already mentioned above, the BP method consists of randomly ordered updates to the marginal probabilities for individual parameters, one at time. Updates continue until convergence, when the marginal probabilities do not change between consecutive updates. We describe the method in detail below for single *W_i_* since the procedure is independent and identical for all rows 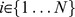 due to Assumption 2. The update calculation is schematically diagramed in Figure 2.

#### Local updates with global information

A local update takes place inside an abstract ‘cavity’, which isolates a single parameter whose marginal distribution is to be updated (*w_ik_*) and data from a single experimental condition (μ). The global information is simply the most up-to-date approximation of the marginal distributions for all the other parameters plus the experimental data. A local update optimizes the balance between fitting the experimental data in the cavity condition *μ* and compatibility with global information, which evolves as the algorithm iterates. A single distribution is locally updated in one step and becomes part of the global information for updating other distributions in successive steps. This local update is repeated in all possible cavities (i.e. all combinations of parameters and conditions) until global convergence of the distributions is reached.

Recall that we are optimizing parameters in a single row of ***W*** so for the following equations the index *i* is fixed. We define *ρ^μ^*(*w_ij_*) to be a factor, which is a probability distribution of a single parameter that describes its fitness to the data in a single experiment *μ* that is compatible with the other parameters. By definition, each factor is independent so that the final marginal is simply the product of all of its factors.

The BP algorithm begins with random *ρ^μ^*(*w_ij_*) for all *μ* and *j*. The initial choice of cavity parameter (*w_ik_*) and cavity condition (*μ*) is also random. Once the cavity is selected, the algorithm collects the global information, which is simply the set of approximations for the marginal probabilities of all the other model parameters (Equation 7). They are approximate in two ways: they lack the contribution from the cavity factor *μ*; and until the algorithm converges, the factors collectively do not describe the true marginal distribution.

*Equation 7: Global information of non-cavity parameters*

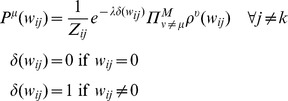



The exponent *λδ(w_ij_)* is an independent penalty for non-zero parameter assignments, which encodes prior knowledge that any parameter is likely to be zero. The superscript *μ* in 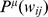 denotes the exclusion of the contribution from experimental condition *μ*. In the following step, the factor *ρ^μ^*(*w_ik_*) is updated to fit the data in a single experiment *μ* given the global information.

#### Calculating local update to *ρ^μ^*(*w_ik_*)

We define *W_i\k_* to be a configuration of the *i^th^* row of ***W*** where the parameter *w_ik_* is fixed to some value *ω*. The factor *ρ^μ^*(*w_ik_*) reflects the fitness of the parameter with *W_i\k_* weighted by the probability of observing the particular configuration **(** *W_i\k_* **)** as in Equation 8.

***Equation 8*** ***: Local update to probability distribution of cavity parameter***

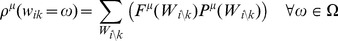
(8.a)

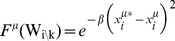
(8.b)


In the field of optimization algorithms, these equations are sometimes referred to as messages because they communicate information between variable nodes and factor nodes on the factor graph, where in this case the variable nodes in the factor graph correspond to model parameters. Thus, BP belongs to a class of ‘message-passing’ algorithms. It is common to see Equation 7 referred to as messages from the variable nodes to the factor nodes and denoted 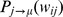. Similarly, Equation 8 can be thought of as a message update from a factor node to a variable node, denoted 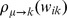.

#### Assumption 3: Independent model parameter distributions

Equation 8 is the mathematical definition of the factor distribution *ρ^μ^*(*w_ik_*). However, a brute force approach for calculating *ρ^μ^*(*w_ik_*) as in Equation 8a is computationally prohibitive. The first complication is that computation of the joint-probability distribution P*^μ^*(*W_i\k_*) is not possible if we consider the interdependencies of the non-cavity parameters. To circumvent this problem, we assume that in the context of the local update, each parameter probability distribution is independent. Then the joint probability distribution can be approximated as the product of the individual parameter distributions.

*Equation 9: Approximation of the joint distribution for the cavity update*

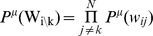



Therefore, Equation 8 becomes Equation 10.

*Equation 10: Local update with factorized probability distributions*

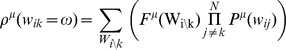



This equation is equivalent to the sum-product formulation, which is standard in BP literature [60]. It is important to note that the assumption that the joint probability distribution factorizes as the product of individual distributions (Equation 9) is exact on tree-shaped factor graphs, but is only an approximation in general. This assumption does not extend beyond the context of the cavity update calculation.

#### Assumption 4: Gaussian mean-field approximation

Another complication in Equation 10 is that a brute force implementation of the sum operation requires enumeration over an exponentially large number of configurations, which in total is K^N*-1*^. Here, we replace the sum over multivariate configurations (*W_i\k_*) with an integral over a single scalar variable (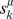). To achieve this, we substitute the fitness function's dependence on the multivariate configuration *W_i\k_* with a single scalar variable 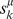. This substitution is explicitly defined in Equation 11, where the new variable 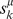 represents the aggregate contribution of the non-cavity parameters to the fitness.

*Equation 11* *: Aggregate effect of non-cavity parameters*

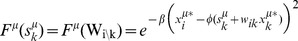
(11.a)

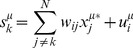
(11.b)


To complete the substitution of *W_i\k_* for 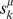 in Equation 10, we also require a description of the probability distribution for the new variable i.e., 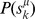. Note that the dependence of 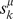 on *W_i\k_* is through a linear combination of the individual parameters (Equation 11.b), which by assumption 3 are independently distributed. We invoke the central limit theorem to approximate 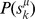 as a Gaussian [66]. The mean and variance of this Gaussian are described by the means and variances of the distributions 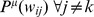. Thus, we replace the sum over multivariate configurations (Equation 10) with the Gaussian integration of 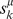 (Equation 12).

*Equation 12: Gaussian integration of local update to cavity parameter*

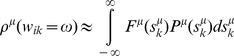



The explicit calculation of 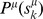 is described in Equations 13a–d, where the over-bar denotes the arithmetic mean.

*Equations 13a* *–* *d* *: Statistical description of mean-field parameters*

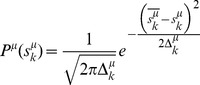
(13.a)

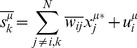
(13.b)

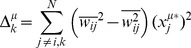
(13.c)

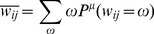
(13.d)


#### Iteration of update equations

In summary, the following BP equations are calculated for each cavity update iteratively until convergence.

*Equations 14a* *–* *b* *: Update equations*

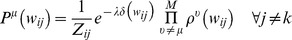
(14.a)

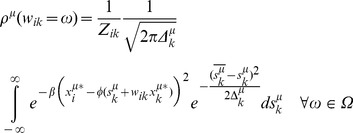
(14.b)


#### Calculation of parameter probability distributions

When the above iterative process converges, the final marginals are calculated from the set of factors, reflecting the information from experimental constraints.

*Equation 15: Final marginal calculation*

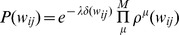



The BP algorithm provides marginals characterizing a set of good models. Thus, we reduce the unbounded model search space to a set of tractable probability distributions for model parameters. Next, one must generate high probability models by drawing from the BP calculated marginals.

#### Network model instantiation by BP guided decimation

We need distinct model solutions to proceed with predictive and quantitative analysis of signaling pathways via explicit model simulations. Distinct solutions are derived by the BP guided decimation algorithm [44]. The decimation algorithm works as follows: (i) an initial BP is run to compute probability distributions *P*(*w_ij_*) for all possible interactions; (ii) a possible interaction (suppose an edge that connects node k and node l) and an associated edge value (*w_kl_ = ω*) is chosen with probability proportional to the corresponding BP marginal; (iii) a subsequent round of BP is run with P(*w_kl_* = *ω*) = 1; (iv) steps i–iii are repeated until an edge value is fixed for all possible interactions in the system. Parallel repetition of this procedure generates any number of network models with varying configurations and error profiles. The non-zero parameters in each model are further optimized using a gradient descent algorithm, which relaxes the discretization of parameter values and further lowers the error by fine-tuning the real number values of the parameters. Moreover, the gradient descent refinement ensures that the network models are mathematically in steady state and the nodes in the network models are fully coupled [33]. Each model is then a set of differential equations describing the behavior of the system in response to perturbations.

#### Direct vs. Indirect Interactions

Due to limitations in experimental measurements, not all proteins and their many phosphorylated states are directly measureable. The result is that many key intermediate players are excluded from the model. For this reason, direct interactions in our model do not necessarily imply direct biological interactions (Supplemental Figure S15). Increasing the number and quality of protein and phospho-protein measurements increases the chance of modeling direct interactions.

### Technical performance

The success of BP depends on whether or not the simulations of the models taken from BP are quantitatively predictive of cellular response to drug combinations. It is also useful, as an exercise, to evaluate the overall performance of the BP algorithm on data sets engineered from completely known networks. With such toy datasets we achieve the following: (i) demonstrate that BP converges quickly and correctly; (ii) compare BP network models to a known data-generating network; and (iii) evaluate performance in biologically realistic conditions of noisy data from sparse perturbations. The synthetic data is generated without the assumptions used in the formulation of BP, and therefore serves as a reasonable test of the sensitivity of the BP method to those assumptions. See methods for more information on how the toy data is generated.

#### Belief Propagation is fast and accurate

Monte Carlo (MC) simulation and optimization is a strategy for sampling the space of explicit solutions, in which full parameter configurations are searched as a whole. Short of infinite coverage, a thorough MC search yields reasonably accurate approximations of the ‘true’ probability distributions: both posterior probability distributions of explicit configurations, and marginal probabilities of individual parameters, which are calculated by counting the frequency of any parameter assignment across the set of good solutions. MC is a frequently used optimization strategy in statistical physics, and thus a valuable candidate for comparison. We examine speed and accuracy performance of MC and BP for increasingly large models. To do this, one toy data generator is constructed for each of the ten different sizes from N = 10 to N = 100. In each case, the number of training patterns equals the number of nodes for consistency of comparison, i.e., M = N. Both methods search a very large parameter space of 41 possible parameter assignments with 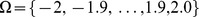; thus for this toy dataset the search space of all configurations is of size 41^N^.

**Figure 3:**
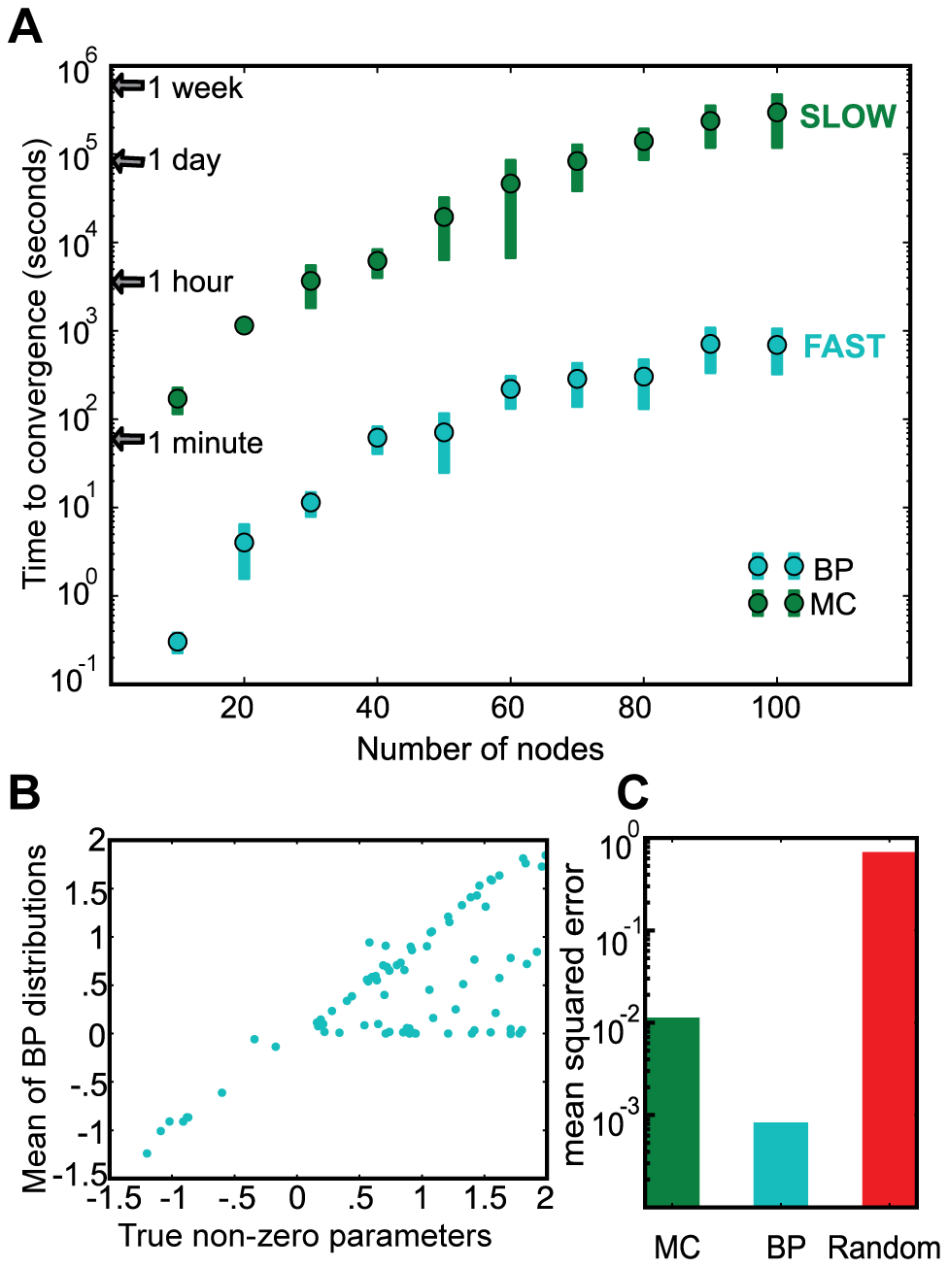
BP is significantly faster than Monte Carlo (MC) with comparable accuracy. (A) BP converges three orders of magnitude faster than MC, even as the size of the system increases to 100 nodes. In this test, the number of training patterns equals the number of nodes in both BP and MC. (B) The means of the distributions from BP are plotted against the true non-zero parameters from the set of the data generators. BP has a high correlation (R = 0.7) with the true parameter values, with many points exactly on the diagonal. (C) MC and BP produce low errors per data point compared to random interaction assignments (Red bar).

The first criterion of interest is time of convergence (Figure 3A). For both methods, the time required for convergence increases as the size of the system increases, but MC is consistently three orders of magnitude slower than BP. The speed advantage of BP is vital for our ability to scale up the size of *de novo* model construction for biological systems with hundreds of nodes.

The second criterion is accuracy. While we are interested primarily in the accuracy of predicting responses to new perturbations, we are also interested in the accuracy of the inferred interactions as an indicator of the models' explanatory and predictive power. In practice, we find that BP does no worse than MC on these datasets given reasonable termination conditions for MC (Supplemental Text-S2).

A Pearson correlation coefficient between the set of non-zero parameters in the data generators and the average values inferred by BP is a reasonable measure of agreement between true and inferred parameters. BP results in a correlation of R = 0.7, (Figure 3B) which is quite high considering the relatively small number of training patterns used for each inference (M = N). As the number and quality of training pattern increases, the correlation approaches 1 (Supplemental Text S3, Supplemental Figures S2 and S3). A more critical metric of accuracy is the agreement with the training data. The average parameter strengths inferred from BP and MC are used to calculate the expected value of the data points. Both MC and BP reproduce the training data very well as quantified by mean squared error per data point (Figure 3C). For reference, both BP and MC errors are at least two orders of magnitude lower than what was expected at random.

While other parameter search methods such as genetic algorithms [67], simulated annealing [68], regression [54], [69], Lasso-regression [54], Hybrid Monte Carlo [70], [71], [72] and Kalman filtering [73] may offer improvements in speed over the standard MC comparison used here, they also suffer from poor scaling properties in the absence of prior knowledge. A statistical-mechanics analysis demonstrates that BP outperforms mutual information based inference algorithms since BP takes into account the collective influence from multiple inputs [74]. We conclude that BP offers a tremendous speed advantage over MC at no observable loss of accuracy.

#### BP reproduces true interactions

BP inference is fast and almost perfect when the system has been sufficiently explored by perturbations. In the case of toy data, one can perturb any set of nodes simultaneously with complete control, and generate information-rich data sets. Use of rich data sets provided a sufficient training set for BP to nearly perfectly infer the underlying system (Supplemental Figure S2 and S3). In biological experimental conditions, however, we are limited by the availability, strength and specificity of the drugs, by the availability of reporters (such as antibodies), and by the technical accuracy of the measurements. We are further limited by the financial and temporal cost of testing all combinations, even for the drugs that are available.

Here, we evaluate BP performance in biologically realistic conditions; small number of sparse perturbations applied individually and in pairs. The inference is repeated with added noise to evaluate sensitivity to noise. The Gene Network Generator GeNGe [75] constructed the structure of positive and negative interactions for the data generator. The data generator network contains several common regulatory motifs, including feedback loops, single/multiple input motifs, multi-component loops and regular chains. For this study, a drug is represented as exhibiting strong inhibition of a main target and smaller positive or negative effects on four or fewer other nodes, which are meant to simulate off-target effects. Complete knowledge of the perturbations and off-target effects is used for the noise-free results, while only knowledge of the main targets is used for the noisy data results, thus mimicking inference on drugs with unknown off-target effects. We simulate the system to steady state in response to each condition of 14 *in silico* drugs applied individually and in pairs. The steady state profiles are recorded and serve as the training data, while those conditions that oscillate are excluded.

Ultimately, the predictive power of the inferred models can only be assessed by explicit simulation of individual models and comparison with experiment. However, the average value of the BP inferred probability distributions can be used in a descriptive sense and either guide human intuitive understanding of biological pathways or be compared to prior knowledge.

The performance features for evaluating the inferred interactions are recall and precision. Recall is the fraction of interactions from the data-generating network that are correctly inferred by BP marginals. False negatives decrease the recall fraction. Precision is the fraction of interactions inferred from BP marginals that are also in the data-generator. False positives lower the precision fraction.

**Figure 4:**
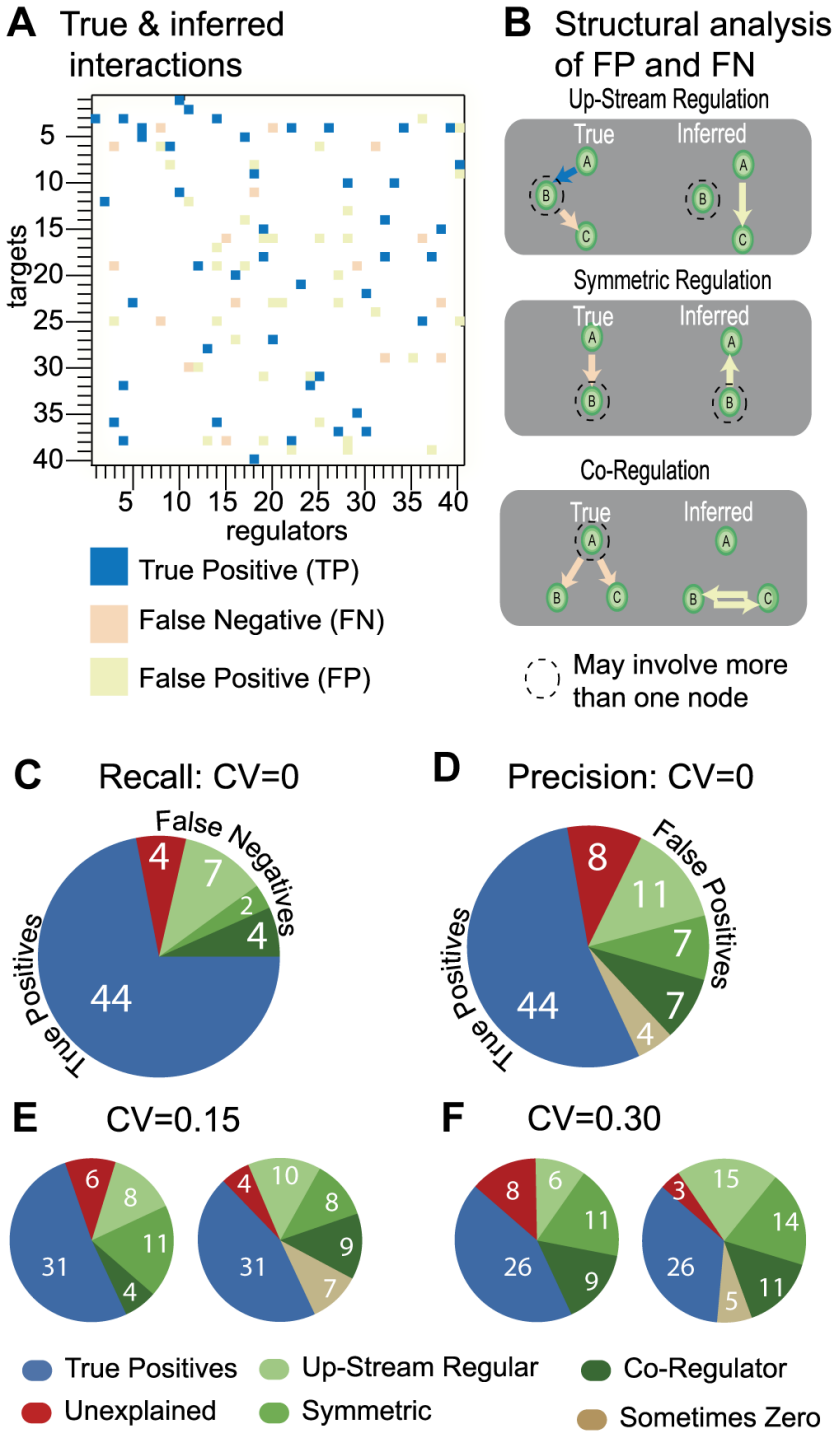
Detailed performance on a single synthetic data-generating network. The average parameters from the BP distributions are compared with the true interactions in the synthetic data generator. The color-coded matrix (A) summarizes all inferred and true interactions. While BP recovers many of the true interactions, some of the interactions are missing (orange; false negatives) while others are incorrect (yellow; false positives). We identified three compensatory motifs (B), which relate false positives to false negatives. Collectively, these classes of compensatory motifs contribute to most of the false negatives (C) and false positives (D). In D, we've also included a category for interactions that have a significant probability of being zero (a non interaction). Even in the presence of considerable noise, (E, F) a significant number of interactions are correctly captured and most of the falsely inferred edges participate in compensatory motifs.

The average interactions from the BP marginals yield a sparse network with a significant number of true interactions (Figure 4A). Importantly, some incorrectly inferred interactions are not mutually exclusive of correctly inferred interactions. That is, since each row of ***W*** is inferred independently (Assumption 2), one might expect a row to be either all correct or all incorrect, yet many rows in Figure 3B have both true and false inferences.

Interestingly, false positives and false negatives are somehow structurally related. In other words, BP tends to miss one or more true interactions (false negatives) and replace them with one or more compensatory interactions (false positives) that are structurally adjacent to the missed interactions. We observe three common structural correlation motifs, which we refer to as (a) upstream, (b) symmetric, and (c) co-regulation motifs (Figure 4B).

In the upstream motif, nodes A and C are connected through an intermediate node B, but an edge is inferred from A to C directly. In the symmetric motif, a false positive connects two directly connected nodes but in the wrong direction. In the co-regulation motif, node A directly regulates B and C separately, but a false positive exists between B and C directly. In addition to being structurally correlated, the numerical correlation between nodes involved in false positives are observably high in the training data (Supplemental Figure S1).

BP misses 17 of the 60 true interactions, giving a recall of 74%. Only 4 of these 17 false-negatives are not involved in one of the three structural correlation motifs. Meanwhile, BP predicts 37 false positive interactions for a precision of 55%. However, 29 of these are either involved in one of the three motifs or have a significant probability of being zero (and therefore ceasing to be false-positives). Consequently, we conclude that while many of the false positives may seem worrisome, they are supported in the data and in the underlying data-generating network.

The results on this toy data confirm that this implementation of BP has trouble disambiguating correlation and causation from steady-state data, which is a difficulty common in analysis of steady-state data [76]. BP infers interactions between highly correlated nodes even if they are not causally connected. BP is better able to infer causality when there is sufficient perturbation of the nodes involved in a potential interaction.

It is likely that the assumptions inherent in this BP algorithm may cause the incorrect edge predictions, in particular assumption 2, which separates the likelihood function from the dynamics of the system. We expect that combining a tailored likelihood function to incorporate time-series data may dramatically improve the ability of a similar BP method to infer causality more efficiently.

#### The inference of network parameters is only moderately sensitive to noise

With toy data, we can accurately analyze the effect that noisy data has on the accuracy of inferring network interactions. Noise from measurement technology can have deleterious effects on network inference and introduce sensitivity to data outliers. For example, RPPA produces Gaussian distributed data in the absence of substantial biological variability [77]. We estimate a coefficient of variance (CV) of 15% on the measurements from RPPA. To examine the effects of Gaussian noise on BP inference, we apply Gaussian distributed noise (*G*_0,γ_) with a mean of zero and a standard deviation of γ representing the CV as in Equation 16.

*Equation 16*

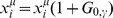



We construct two data sets with added Gaussian noise; one with a realistic CV of 15% (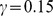) and one with high CV of 30% (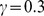) as a worst-case scenario. Though both recall and precision decrease with added noise (Figure 4E, 4F), the number of unexplained interactions stays roughly constant. Importantly, BP is still able to identify key regulatory influences from the noisy steady-state data from sparse perturbations without any dependence on prior knowledge.

This analysis of BP inferred interactions is limited to a thorough examination of a single set of interactions, taken as the average of each parameter from the BP generated marginal probabilities. We demonstrate that BP is fast, accurate and minimally sensitive to realistic amounts of noise. Moreover, BP is sufficiently strong in distinguishing causal from correlated relationships even though we are currently limited to steady-state data from a small set of perturbation conditions. We know how good the inference of interactions is in a scenario where a perfect model of the data generator exists. The comparison gives us an idea of the structural plausibility of the interactions inferred in real biological contexts.

### Network models of signaling pathways in melanoma cells

The probabilistic nature of the BP algorithm is the key feature that enables *de novo* inference on large and complex problems in cell biology, e.g., signaling processes involving more than a hundred molecular and phenotypic variables, which previous methods could not reach. Given this opportunity, we apply our network pharmacology approach to SKMEL-133, a melanoma cell line resistant to the RAF inhibitor (Vemurafenib, PLX4032), which inhibits the *BRAFV600E* mutant protein kinase more strongly than other RAF proteins.

#### Experiments: Systematic perturbation of SKMEL-133 cells using drug pairs

**Figure 5:**
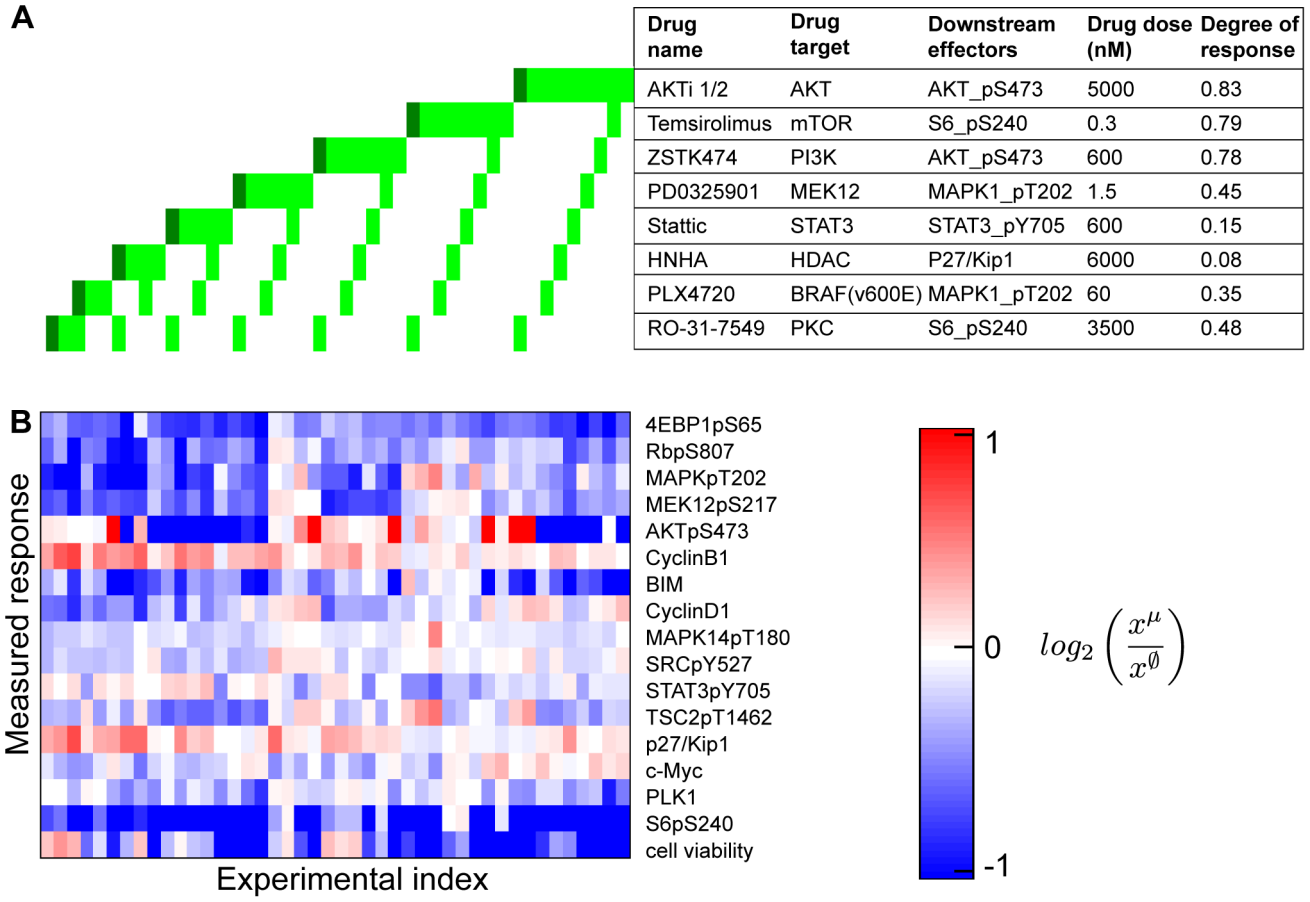
Systematic perturbation experiments. (A) Perturbation experiments with systematic combinations of eight small molecule inhibitors, applied in pairs and as single agents in low (light green) and high (dark green) doses. The perturbation agents target specific signaling molecules, detailed in the table. The listed drug dose is the standard drug dose (light green), and two times the standard dose was used for the high dose conditions (dark green). The degree of response is the approximate ratio of downstream effector levels in treated condition compared to untreated condition. (B) The response profile of melanoma cells to perturbations. The response profile includes changes in 16 protein levels (total and phosho-levels, measured with RPPA technology) and cell viability phenotype relative to those in no-drug applied condition. The slashed-zero superscript denotes the unperturbed data.

We systematically perturb SKMEL-133 cells with a panel of 8 targeted drugs (Figure 5) used singly and in pairwise combinations totaling 44 unique experiments. The set of perturbations includes the 8 drugs applied individually at two unique doses (low and high) and all pairs of the 8 drugs at the single low dose. The drugs are selected on the basis of their target specificity, availability and usability in clinical settings. The selected drugs predominantly target the PI3K/AKT and MAPK pathways, which are known to affect the response to RAF and MEK inhibitors in some melanoma cell lines and clinical samples [78], [79], [80], [81], [82]. Drug doses are chosen based on the measured effect of each single drug on a presumed downstream effector of its target. The changes to presumed targets are measured with Western blot experiments in different drug concentrations, and protein IC40 values are estimated from the dose-response curve (Supplemental Figure S4). Protein IC40s are the drug concentrations that reduce the abundances of a presumed downstream effector by 40%; compared to proliferation IC-values, which relate drug doses to phenotypes such as cell viability, protein IC-values tend to be much smaller than the proliferation IC-values.

As an example, the AKT inhibitor (AKTi) concentration is chosen based on reduction in AKT phosphorylation at S473 (AKTpS473). The drug dose response curve indicates that ∼5000 nM of AKTi is required to reduce AKTpS473 levels by 40% compared to untreated controls. Therefore, 5000 nM is the so-called protein IC40. In this study, we choose to work with protein IC40 concentrations, which is a compromise between the competing requirements of gentle perturbations and observable effects.

The main intent of the systematic perturbations is to explore diverse aspects of the signaling response and to maximize information in the response profiles for model inference.

#### Experiments: Observation of response profiles in SKMEL-133 cells

We use an array technique (reverse phase protein arrays, RPPA [83]), in which cell lysates are interrogated by antibodies against proteins and phospho-proteins of interest. Compared to Western blot assays, RPPA has the advantage of higher throughput, higher sensitivity and better dynamic range. These are crucial advantages for the quantitative inference of network models. However, unlike Western blots, RPPA cannot separate stain based on size, which limits the number of suitable antibodies and puts high specificity requirements on RPPA antibodies. Three independent biological replicates are spotted for each experimental condition. After perturbation, we quantify the response for each protein as the log_2_-ratio of the measured level in the perturbed condition against the measured level in the untreated control condition. In this work, a response refers to a log_2_-ratio value. Viability of the cells after drug perturbation is measured using a resazurin assay 72 hours after the cells are perturbed.

#### Concept of network models of signaling in SKMEL-133

Collectively, the responses for 16 protein/phospho-proteins and the cell viability phenotype from the 44 perturbation conditions constitute the training data for *de novo* network inference. We also include 8 activity nodes (see Methods section) to represent the perturbed but unmeasured activity of the drug targets. We build network models of signaling pathways in the melanoma cell line SKMEL-133 to predict the response of inhibition to single and multiple protein targets. We expect the network models to generate hypotheses about previously unidentified interactions, some of which may be accessible to biochemical experimental tests. Further, these models provide a quantitative and intuitive model of the signaling cascades in SKMEL-133 cells and predict novel single drug targets that may be effective in reducing cell proliferation. In future work, we expect similar models will guide discoveries to overcome or prevent the emergence of drug resistance.

#### Leave-k-out tests of predictive power of network models

In order to test the accuracy and the predictive power of the models, we use a leave-k-out cross validation test. For each leave-k-out test, we withhold k experiments from the training data, infer network models from the corresponding subset of training data, predict response profiles (via simulation) for the withheld perturbation conditions, and then compare the predicted profiles against those from the withheld test data. Specifically, each leave-k-out test focuses on the removal of a single drug from the training data; all combinations involving the drug of interest are removed, leaving only data from the experiment in which the drug of interest was applied alone (single dose). For each test, we generate 1000 network models with the BP guided decimation algorithm and keep only the top 100 models (those with lowest error in the training set). Overall, eight sets of network models are generated; one for each of the 8 unique drugs.

**Figure 6:**
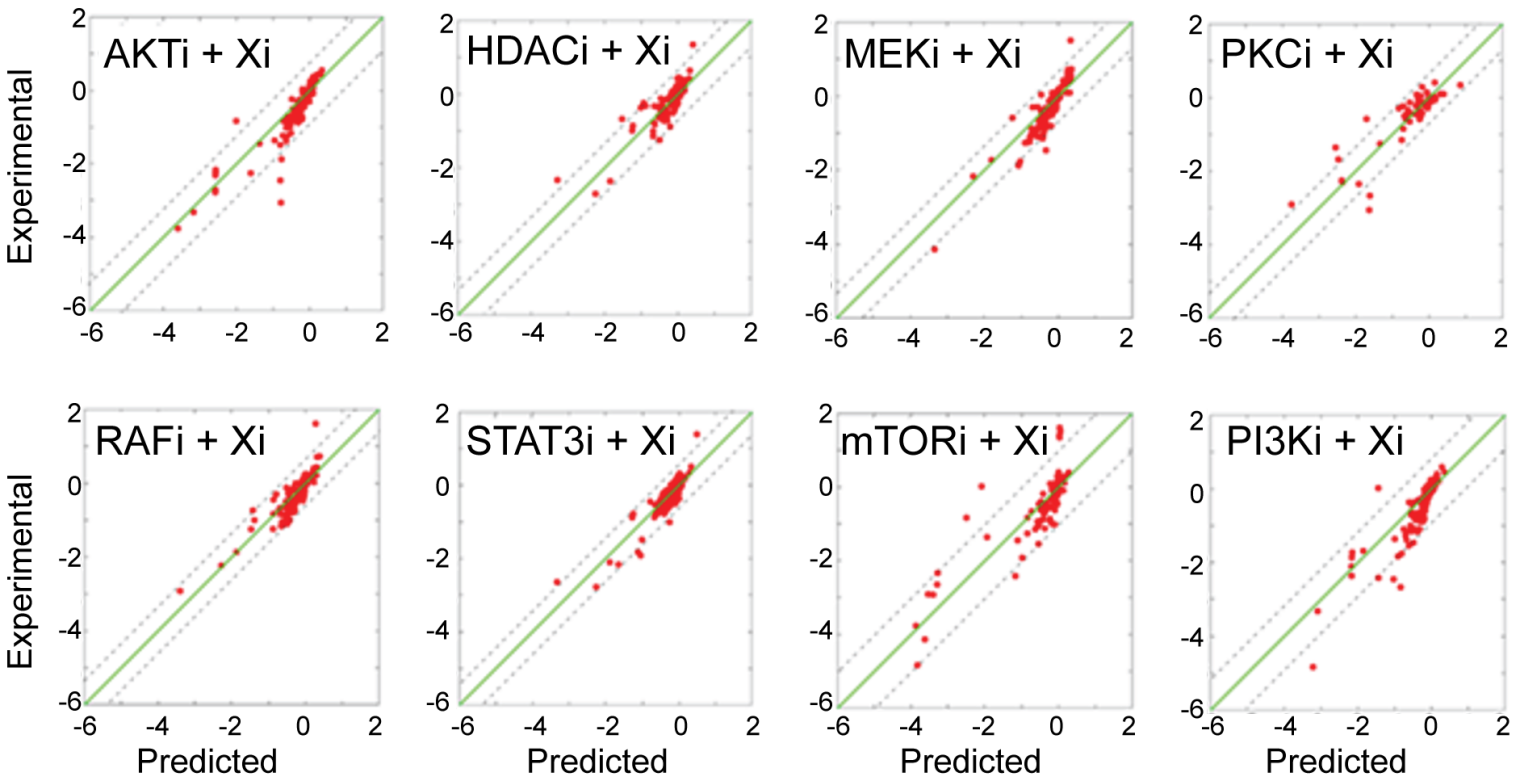
Predictive power of network models. Eight distinct leave-7-out cross validation calculations indicate a strong fit between the predicted and experimental response profiles. In each cross-validation experiment, network models are inferred with partial data, which lacks responses to all combinations of a given drug. Next, network models are executed with *in silico* perturbations to predict the withheld conditions. The cumulative correlation coefficient in all conditions between predicted and experimental profiles is 0.87 (CV = 0.05). Few prediction outliers deviate from experimental values more than 1 σ (standard deviation of the experimental values, dashed lines).

High correlations between simulated and withheld experimental profiles indicate substantial agreement for each of the 8 independent tests (Figure 6). The comparison reveals an overall Pearson correlation coefficient of 0.87, with a cross-validation error CV = 0.05 (see Methods) between experimental and predicted response profiles (Supplementary Figures S7 and S8). Such high correlation suggests genuine predictive power.

#### Average network as a guide

**Figure 7:**
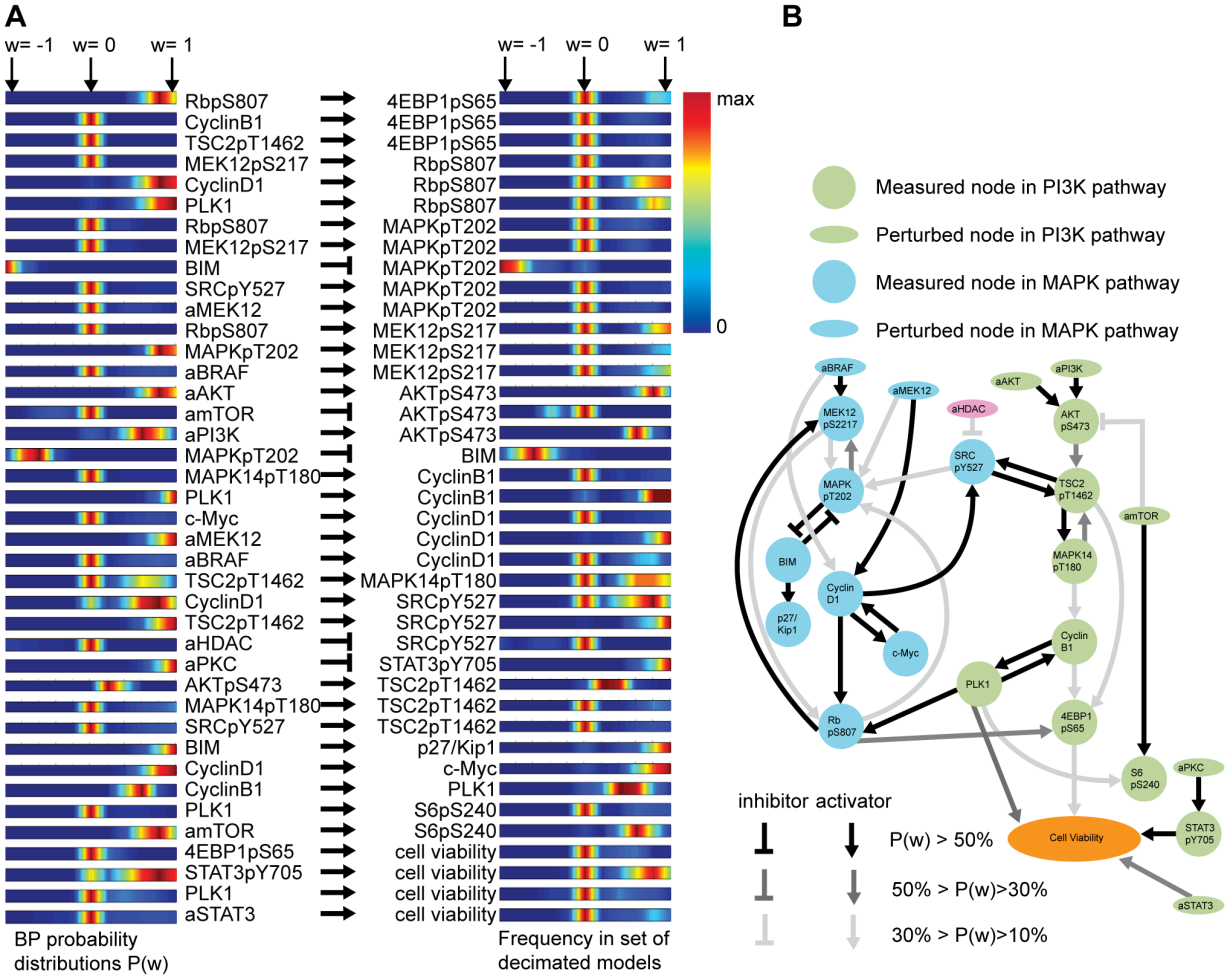
The distribution of edges in all network models and average network model. The probability distribution of edge values (*w_ij_*) after BP (A, left) are similar to the histograms of the corresponding interactions after decimation (A, right). An interaction strength is nonzero when it has high amplitude and frequency in solution space. We generate instantiated models with BP guided decimation algorithm followed by gradient descent optimization. According to the agreement between the distributions in two panels, BP probability distribution and final model histogram are similar to each other with important exceptions. The BP guided decimation algorithm goes beyond simply sampling from the BP models and may encounter features such as mutual exclusivity during the creation of the final models. (B) The average network model over the 100 best solutions with lowest error capture known interactions such as those in the RAF/MEK/MAPK and PI3K/AKT pathways. The opacities of the edges scale with the absolute probability of the edges. Note that the letter ′a′ is prefixed to so-called activity nodes as explained in Methods section.

1000 unique models are drawn via the BP guided decimation algorithm. From those, the top 100 models are kept for the analysis. The marginal distributions before decimation (Figure 7A, left) and after decimation (Figure 7A, right) reveal substantial similarity. Some notable discrepancies (e.g., interaction from RbpS807 to 4EBP1pS65) may reflect the effects from mutually exclusive interactions, where two high probability interactions never occur in the same model configuration. Such phenomena illustrate the value of recalculating BP to handle conditional dependencies as interactions are fixed during model construction.

For simplicity of interpretation and visualization, a single network representing the set of average interactions, called the ‘average network’, is presented (Figure 7B). These are the average interaction strengths across the top 100 models. Note that the average network is not suitable for simulation-based predictions. However, the average network depicts the qualitative features of the high probability region of solution space by reporting interactions that are present at high frequency across a set of good models. Remarkably, the average network captures many of the signaling interactions within the major pathways important for melanoma progression, particularly the canonical (accepted as standard) MAPK or PI3K/AKT pathways. In addition, the average network is suggestive of a series of additional interactions (see Supplementary Table S1 for details), some of which may represent novel biochemical interactions and are good candidates for follow-up experimental investigation and validation.

The network's description of pathways is limited to the scope of observed model variables. The details of our network may deviate from the intricate details of canonical pathway models due largely to the existence of unobserved nodes. As a natural consequence, the predictions we generate from our networks are also limited by the scope of observed model variables. As the scope expands, the network models may converge to the detailed pathway descriptions with additional capacity for context-specific quantitative predictions.

#### Interpretation: AKT pathway

The canonical PI3K/AKT pathway is characterized by a series of complex interactions resulting from the activity of the upstream kinase PI3K and reaching more downstream regulatory proteins [84]. Although the detailed spatiotemporal regulation of the PI3K/AKT pathway and its phenotypic output ranging from proliferation to metabolic changes are highly complex, our average network captures the major known interactions in this pathway.

The indirect positive effect of PI3K on AKT phosphorylation, phosphorylation of TSC2 on T1462 by AKT activity, the regulation of S6 and 4E-BP1 by the PI3K/AKT pathway are all represented in the inferred average network. In our network, amTOR, the activity of mTOR, has no upstream node. This is an artifact of our modeling approach, which prohibits incoming interactions to unmeasured activity nodes. However, the interaction connecting TSC2pT1462 to 4E-BP1pS65 links the upstream components of the pathway to the downstream components. An inhibitory interaction from amTOR to AKTpS473 in the PI3K/AKT pathway is reminiscent of the reported feedback loops in this pathway [85].

#### Interpretation: MAPK pathway

In the RAF/MEK/ERK pathway, the average network captures many of the known interactions that link the MAPK activity to Cyclin D1 levels and Rb phosphorylation [86]. However, the interactions that link this pathway to cell viability are indirect through phosphorylated 4E-BP1 and have a relatively low effect on this phenotype (See below for a quantitative predictions). The network has a bidirectional interaction between PLK1 and Cyclin B1 [87]. A strong direct interaction between PLK1 and cell viability is also present in the network, consistent with the known PLK1 and Cyclin B1 regulation of the G2 to M transition in the cell cycle and the multifaceted role that PLK1 plays in mitosis, including spindle formation [87].

#### Prediction: Logical and biochemical interactions in the melanoma cell line

In addition to the agreement with well-studied biological interactions, the average network also indicates a series of potentially novel interactions.

First, a strong bidirectional interaction is inferred between RbpS807 and MEKpS217. One hypothesis for the MEK to Rb interaction is that it stands in for the known direct RAF to Rb interaction in the absence of any measurements of phosphorylated RAF. Disruption of the RAF to Rb interaction induces apoptosis in melanoma [88], [89]. Conversely, the Rb to MEK interaction has not been previously reported. Given that RAF, RAS, and MAPK are all in the same pathway with MEK, the Rb to MEK interaction is plausibly consistent with the observations that: (i) *RB1* (the gene that encodes the Rb protein) and *KRAS* double-knockout mice studies indicate genetic interaction between Rb and KRAS [90]; and (ii) inactivation of Rb results in elevated RAS and MAPK activity [91], [92]. It is important to note that the phosphorylation of Rb on S807 is an inhibitory modification, so a positive interaction from RbpS807 to MEKpS217 is consistent with Rb inhibition causing an increase in phosphorylated MEK levels.

An inhibitory interaction from aHDAC (activity of HDAC) to SRCpY527, a critical phosphorylation site for the auto-inhibition of SRC [93], is consistent with the direct and indirect interactions of HDAC isoforms with Src observed in multiple cancer contexts [94], [95]. Finally, positive interactions connecting Cyclin D1 and TSC2pY1462 to the SRCpY527 node are inferred. To the best of our knowledge, no such interactions have been reported. The observed edges may correspond to logical indirect interactions that coordinate negative feedback loops acting on Src from the MAPK and PI3K pathways. All of the predicted interactions are suggestive of follow-up genetic and/or biochemical experiments.

#### Prediction: drug target identification in SKMEL-133 via computation of viability changes in response to previously untested perturbations

Given a set of network models of SKMEL-133 cells, we predict the effect of arbitrary *in silico* perturbations acting on any protein node present in the model. Such *in silico* perturbations predict the quantitative effects of hypothetical drugs (i.e., virtual drugs that target the nodes in a model) on the cellular phenotypes. Furthermore, one can quantitatively track signaling processes beyond the qualitative interpretation of the average network model.

**Figure 8:**
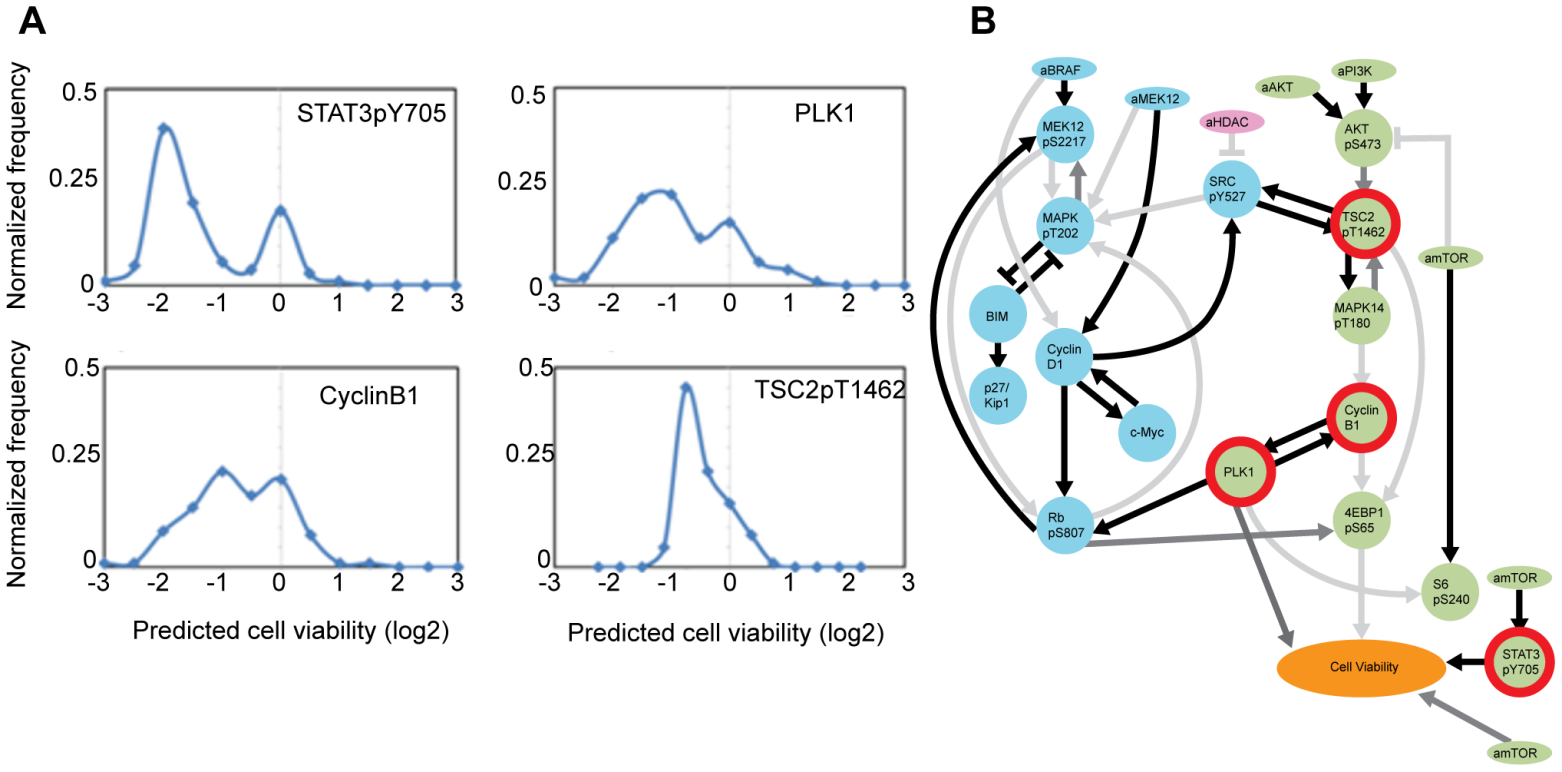
Novel predictions from *in silico* perturbations. (A) The histogram of phenotypic response profiles to the four most effective virtual perturbations from the best 100 network models. The response to STAT3p705 reflects the effect of PKCi on cell viability. Viability changes in response to perturbations on cell cycle proteins PLK1 and Cyclin B1 are genuine predictions from the network models. Perturbation of TSC2pT1462 (inhibitory phosphorylation) down regulates the PI3K/AKT pathway and leads to a decrease in cell viability in the PTEN null SKMEL133 cell line. (B) The perturbed nodes that lead to reduction in cell viability in the context of average network model (Circled in red).

As an example, we predict the cell viability response of SKMEL-133 cells to various targeted perturbations via explicit numerical simulations of our network models. The simulations for each of the network models produce predictions of the temporal trajectories for all model nodes: proteins, phospho-proteins and phenotypes, alike. In each simulation, a single perturbation to a single protein node *i* is encoded as an external force (*u_i_*) that reduces the relative abundance of that node by 50% (*x_i_* = log_2_(0.5) = −1), which in turn propagates throughout the network model. The simulated steady-state responses to four particular, individually perturbed target nodes predict substantial decrease in cell viability (Figure 8) and therefore represent potential novel, efficacious drug targets.

The most significant reduction in cell viability comes from the predicted response to perturbation of STAT3pS705, whose mean predicted inhibition of cell viability is roughly 56%, i.e., 44% of the unperturbed cell viability (*x_i_* = −1.16). In the average network, STAT3pS705 is downstream of aPKC, and our perturbation experiments do show that PKC inhibition leads to low cell counts. Thus, the response of STAT3pS705 inhibition simply reflects the effect of known PKC inhibition, which is included in the training set. Next, perturbation of both PLK1 and Cyclin B1 protein nodes lead to significant reduction in cell viability at 45% (*x_i_* = −0.85) and 38% (*x_i_* = −.69) inhibition, respectively. PLK1 and Cyclin B1 are two important cell cycle proteins regulating G2 to M transition and highly specific PLK1 inhibitors are potential agents in targeted therapy [96]. Importantly, the network approach predicts the effect of PLK1 and Cyclin B1 perturbations on cell viability without any training to responses of perturbations to these nodes.

**Figure 9:**
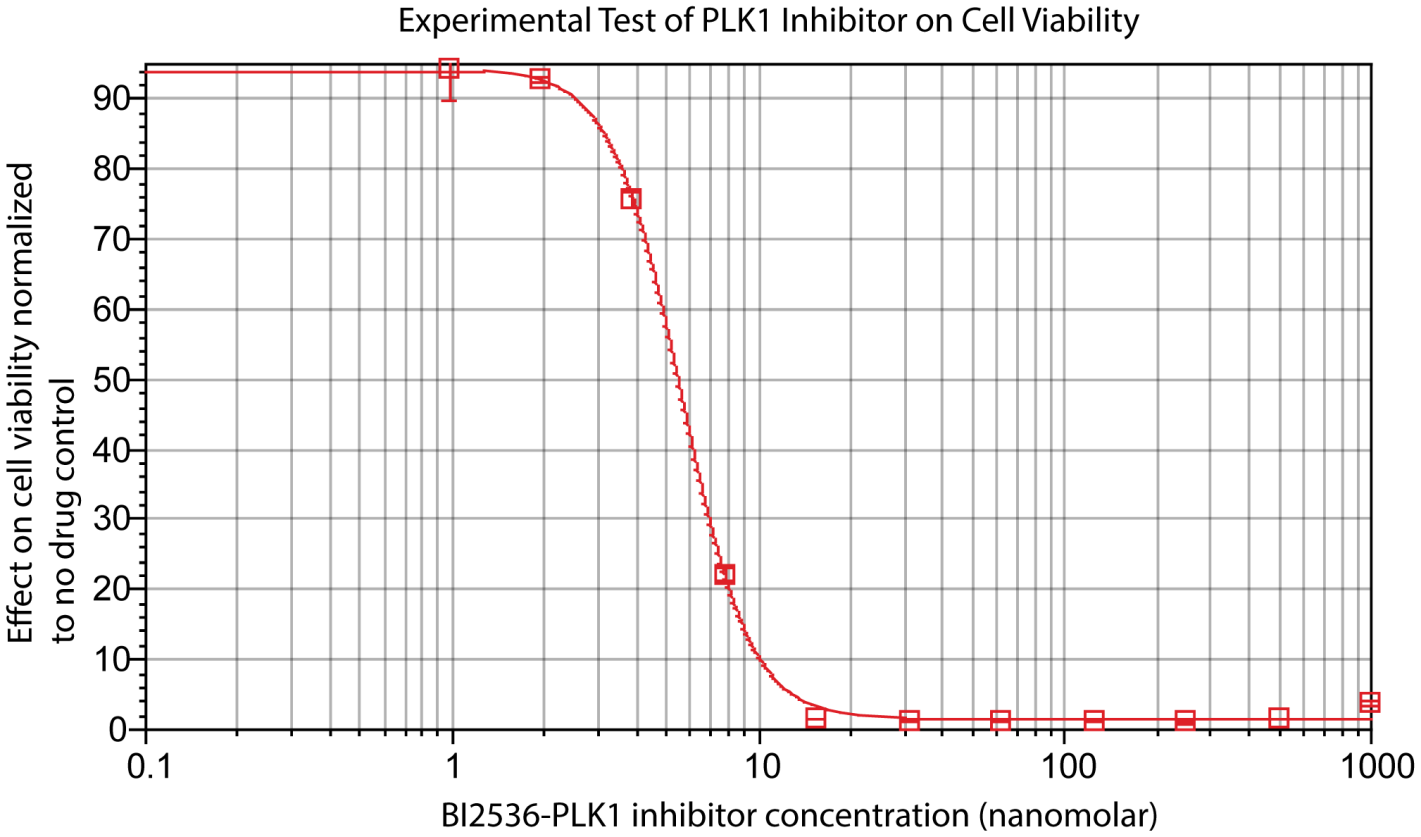
Experimental testing of computational predictions. Qualitative analysis of networks from *in silico* simulations nominates PLK1 and Cyclin B1 as potential targets to kill RAF inhibitor resistant melanoma cells. A validation experiment with the PLK1 inhibitor BI2536 shows extensive growth inhibition in SKMEL133 cells (Cell viability IC50 = 5.8 nm). PLK1 inhibitor is a pure prediction of the approach and was not included in the experimental drug set.

We subsequently tested one of these predictions in the lab by treating the SKMEL-133 cells with the PLK1 inhibitor (BI 2536) and measuring cell viability response with the resazurin assay (Figure 9). The experimental tests reveal that the cell viability IC50 for the PLK1 inhibitor is 5.5nM in SKMEL-133 cells and that approximately 99% of the cells are eliminated with a 15nM concentration.

The fourth perturbation that predicts significant change in cell viability is TSC2pT1462 inhibition, which has a central role in the average network model, particularly in PI3K/AKT pathway. It is regulated by AKTpS473 and also interacts with MAPK14pT180, which is upstream of PLK1 and Cyclin B1 in the models. Note that SKMEL-133 cell line is PTEN-null and that a constitutively active PI3K/AKT pathway may play a role in drug resistance in this cell line [46]. Thus, it is not surprising to us that deactivation of a central player in this pathway leads to reduction in cell viability. The corresponding best drug target is the kinase that leads to phosphorylation of TSC2 on T1462.

The predicted effect on cell viability for each of these four computationally perturbed nodes has at least one of the following explanations: consistent with known perturbation responses of immediately adjacent nodes, present in model training; consistent with the known genetic background of the model cell line (e.g., SKMEL-133 is PTEN-null); or represents a genuinely novel drug target, predicted to be highly efficacious and subsequently validated experimentally.

## Discussion

### Beyond classical molecular biology

We describe a combination of experimental and computational methods, in the field of network pharmacology, to construct quantitative and predictive network models of signaling pathways. The particular contribution is a set of algorithmic advances, which we adapted from statistical physics to infer network models in sizes and complexities not reachable by classical gene-by-gene molecular biology. The necessity of inference of complex network models stems from the fact that classical methods, in which a small number of perturbation experiments lead to functional description of carefully selected sets of genes and gene products is reaching technical limits. High-throughput proteomic and genomic profiling technologies provide much richer and more complex information about cellular responses than can be analyzed by a scientist's thought processes. At these levels of completeness and detail, predicting changes in physiological attributes from molecular data requires computational modeling and quantitative analysis. Our quantitative network models not only capture already known biological interactions but also nominate novel interactions and may detect complex regulatory mechanisms, such as feedback loops, in specific biological contexts. The quantitative analysis of molecular and cellular behavior in these models provides detailed understanding of the coupling between signaling processes and global cellular behavior. Such understanding is hard to achieve by reductionist approaches that focus on the relation between single or few molecules and cellular processes. Furthermore, we provide a systems biology platform to predict the cellular (i.e., proteomic and phenotypic) response profiles to multiple perturbations such as those in combinatorial cancer therapies.

### Network pharmacology in the era of personalized medicine

This investigation constitutes a proof-of-principle of a particular technology: a combination of a network inference algorithm and a technology for perturbing cells and measuring their response. The overall context is network pharmacology, by which we mean the science of using network models to derive and then test effective therapeutic targets and combinations. The particular challenge in cancer biology is the complexity and individual variation of genetic and epigenetic alterations that are plausibly cancer causing, and thus modulate the response to therapy; and, the emergence of resistance to successful targeted therapies, such as EGFR inhibitors in lung cancer or RAF inhibitors in melanoma. In our view, the fairly fragmented gene-by-gene classical methods of molecular biology, while extremely powerful as a reductionist method, are reaching a clear limit. Those methods struggle with effects such as ‘cross-pathway coupling’, ‘multigenic diseases’ or individual variation of response to therapy. More comprehensively quantitative and computationally predictive methods (‘systems biology’) are likely not only to increase our predictive abilities but also save substantial overall effort by computationally testing large numbers of cellular states on a large variety of genetic backgrounds and in exhaustively explored perturbation conditions.

### Power and limitations of the belief propagation method for network modeling of signaling pathways

The implementation of our BP algorithm enables us to infer much larger network models than are reachable with standard Monte Carlo search methods. First, we use biologically realistic toy data to illustrate the dramatic speed improvement of the BP method over standard MC methods. BP convergence times scale favorably with the size of the models at no measureable loss of accuracy. This is a desirable property for constructing large network models *de novo*. Even in the biologically realistic conditions of noisy data from sparse perturbation, BP infers most of the dominant interactions in the data-generating network. Although a fraction of the likely BP interactions are false positives, these false positives are consistent with the data and connect structurally correlated nodes. The assumptions inherent in this BP implementation are potential limitations to the overall efficacy and accuracy of our approach. However, assumption 1, while important, does not dramatically affect the results beyond a critical level of discretization. Furthermore, since BP performs as well or better than MC, which employs neither assumption 3 nor 4, we suspect by process of elimination that assumption 2 is the largest contributor to the observed limitations of our current method. For real biological data, the quality of BP's performance depends on the applicability of the model equations and both the richness and quality of the data used for training.

We previously published an MCF7 breast cancer cell line dataset, for which network models were derived with a nested MC search algorithm (CoPIA) [97]. Reassuringly, many of the strongest interactions from CoPIA are recovered by the BP method (Supplementary Text-S4, Figure S10). We have also modeled the same SKMEL-133 dataset with Gaussian Graphical Models, a popular probabilistic model that goes beyond pairwise correlations to distinguish between direct and indirect correlations. A comparison to the BP derived interactions suggests non-trivial overlap in the strongest couplings. A more detailed analysis is available in the supplement (Supplementary Text –S5, Figures S11 and S12). Finally, we have also compared BP against both prior knowledge models and Bayesian network models inferred with Bayesian inference [98]. That analysis indicates superior performance for the BP derived models for at least this kind of data and this style of model (See Supplemental Text S7 and Figure S17).

### Confirmed and predicted interactions in malignant melanoma cells

We are able to capture the known interactions in MAPK and AKT/PI3K canonical pathways through *de novo* network inference. Moreover, we predict potential interactions, either direct or indirect, such as a bidirectional interaction between Rb and MAPK pathways and a potential feedback loop from PI3K and MAPK pathways acting on SRC activity through inhibitory phosphorylation at SRCpY527. We also quantitatively predict the effect on cell viability from inhibition of novel targets via explicit *in silico* simulation. The *in silico* perturbations of Cyclin B1 and PLK1 lead to comparable reduction in cell viability. As neither Cyclin B1/PLK1 nor their direct regulators are inhibited for the model training dataset, this result is a genuine and nontrivial prediction. We test and validate the PLK1 prediction by measuring the cell viability after treating SKMEL-133 cells with a potent and selective PLK1 inhibitor. Indeed, the PLK1 inhibitor reduces cellular growth significantly in SKMEL-133 cells even when treated with the drug at nanomolar concentrations (Figure 9). Moreover, none of the *in silico* perturbations of the MAPK pathway lead to a significant change in cell viability, which is consistent with the experimental responses to perturbations acting on MEK and BRAFV600E and RAF inhibitor resistance of SKMEL-133 cells.

### The problem of drug specificity

We are aware that drugs do not usually have a single specific target. While this is potentially problematic for modeling and simulating drug effects, we find that the inference is driven largely by the correlations in the data arising from the effect of perturbations on the overall system. We estimate that BP is most sensitive to strong and untrue assumptions about the direct effects of a given drug. The use of activity nodes is an indirect way of dealing with off-target drug effects. These nodes, which are perturbed but not measured, represent the coupling of a drug to the rest of the system. Such coupling includes both specific and off-target effects. BP may infer interactions from these activity nodes to any number other measured nodes thus simultaneously inferring targets for the particular drug used. Drug specificity also affects the predictive power of the resulting models. All effects from the AKT inhibitor, for example, are assigned to single inhibition of the aAKT node, even if other targets of the AKT inhibitor are partially responsible for the measured outcomes. Therefore, any simulation-based prediction regarding perturbation of the aAKT node will be tethered to the off-target effects of the AKT inhibitor used in training. The complete solution of the drug specificity problem requires a more comprehensive and systematic analysis to determine the effect of off-target effects on quality and predictive power of inferred models.

### Failed predictions and optimal design of experiment

The leave-k-out analysis reveals few outlier points, where the predicted response profiles largely disagrees with test data. These (mis)predictions fall into two major categories. In the first category, (mis)predictions arise due to measurements with very low signal to noise ratios and high experimental uncertainties. The (mis)predicted S6pS240 levels and cell viability phenotypes in some of the perturbation conditions fall into this category. Note that the models are trained and simulated after logarithmic conversion (i.e., *x_i_* = log_2_-ratios of measured signals), which exaggerates the errors for low signals. In linear space, no such outliers are observed suggesting that those outliers can be considered as an artifact of our analysis in logarithmic space. In the second category, (mis)predictions arise due to insufficient experimental constraints. (Mis)prediction of AKTpS473 levels after mTOR inhibition falls into this category. mTOR inhibition leads to an increase in AKT phosphorylation possibly due to the disruption of a feedback loop. In our current analysis, mTOR inhibition and steady state measurements on AKT phosphorylation are the sole experimental inputs to detect the changes in this feedback mechanism. When the mTOR inhibition is withheld in leave-k-out tests, experimental constraints become insufficient to describe the regulation of AKT phosphorylation, thus leading to the (mis)predictions. A systematic experimental design to enrich the perturbations, better characterization of the AKT phosphorylation dynamic range and richer proteomic measurements on this part of signaling pathways are possible ways to improve the quality of these particular predictions. In general, careful optimization of perturbation conditions (drugs and combinations) and observations (protein arrays, mass spectrometry target list), within available resources, would significantly enhance the predictive power of this approach.

### Predicting effective novel drug combinations with BP based network modeling

Predicting the effect of drug combinations is highly challenging and has been the subject of many studies, e.g., [99], [100]. When a large repertoire of targeted drugs are available for screening, the search space for useful combinations of two or more drugs is combinatorially complex. Moreover, therapeutically promising drug combinations are not limited to simultaneously introduced perturbations. Potentially useful drug combinations may consist of combinations of relative doses, for two, three and four drugs applied simultaneously or sequentially after well-defined time intervals. Identification of such complicated combinations through experimental screening tests is prohibitively cumbersome. Here, we provide a potential solution to this problem in that *in silico* screens using predictive network models can cover a large space of possible drug combinations. The predictive power of the models derived here is apparent from the reasonable accuracy of predicting the results of withheld experiments using a leave-k-out cross validation. Network models inferred from perturbation experiments can thus be used to predict the responses to novel combinations. One can enlarge the search space for drug treatments from a few hundred experimentally screened combinations to tens of thousands of computationally tested combinations and guide subsequent, highly efficient experimental screens of the top predicted candidates.

Beyond the computational power of well-constrained and robustly derived network models, one may expect to achieve a conceptual understanding of the principles of epistasis of drug effects and the mechanisms of resistance to targeted therapeutics. For example, the initial system response to drug intervention on the scale of minutes to days may be indicative of subsequent epigenetic and genetic changes in a population of treated cells that represent the long-term and hard-to-treat emergence of drug resistance. In this context, reliable dynamic network modeling may be an excellent guide to strategies for blocking the emergence of resistance in the first place. The pre-clinical consequence is the selection of combinations of therapeutic interventions that not only are effective in slowing the proliferation or promoting the elimination of cancer cells, but also counteract resistance to otherwise effective treatments, such as RAF inhibitors in melanoma or AR inhibitors in advanced prostate cancer, in clinical trials.

### Method improvement

While the approximate solutions to the problem of network inference deliver interpretable biological results, there is much room for improving the power and information value of the method. For example, when time-dependent response measurements after perturbation are available, one can use analogous, extended algorithms to infer probability distributions over (time-independent) interaction parameters ***W*** that describe the behavior of a time-dependent system. Another extension of the formalism is the use of more complicated forms of the differential equations, such as those of enzyme kinetics. Also, even in the current approximation, careful design of experiments selecting a minimal set of maximally informative perturbation conditions would increase the efficiency of this experimental-theoretical approach.

A straightforward and powerful extension is the systematic use of prior information in the form of directed interactions adapted from the current scientific literature or pathway databases. Such prior information is easily incorporated as a set of additional constraints on the probability distributions of ***W*** in Equation 3. This has been utilized in our related work on modeling dedifferentiated liposarcoma drug combinations [43]. We are also actively pursuing a method for the systematic inclusion of prior knowledge interactions from curated databases. On the experimental side, measurement of richer phenotypic attributes of cells, such as apoptosis or cell cycle arrest, as well as markers of differentiation states would greatly increase the predictive power by providing more links between molecular and phenotypic quantities.

The network pharmacology approach described here provides a strong tool for a system level description of signaling events in cancer cells. Moreover, it presents a step forward in quantitative prediction of responses of cancer cells to drug perturbations. Beyond cancer biology, there is no reason to believe that the proposed technology cannot be used to derive accurate quantitative and predictive network models for biological cellular systems in general, provided sufficiently diverse experimental perturbations and sufficiently rich readouts are accessible. In this way, we hope to extend the power of classical molecular biology to a broad spectrum of cellular systems with targeted, and possibly clinical, applications.

## Materials and Methods

### Materials

#### Choice of drugs and drug concentrations

Eight small molecule drugs targeting mainly the MAPK or AKT/PI3K pathways were chosen based on the knowledge of target specificity and relevance for exploring BRAF signaling in SKMEL-133 cells (Figure 5). In order to select an appropriate drug concentration for the RPPA assay, Western blots were used to measure the dose-response effect of each drug on its presumed targets or downstream effectors (Supplementary Figure S4). We use the so-called ‘protein IC-values’, which are the concentrations that inhibit the most immediate downstream protein by a certain percentage. This is in contrast to the more common proliferation IC-values, which are the concentrations that reduce the number of proliferating cells by a certain percentage

#### RPPA and western blots

For Western blotting and reverse-phase protein arrays (RPPA) assays, BRAFV600E mutant SKMEL-133 cells were grown in 6-well plates to around 40% confluence in RPMI-1640 medium containing 10% fetal bovine serum (FBS). In a series of perturbation experiments, cells were perturbed with 8 drugs either singly or in paired combinations, and harvested after 24 hours by collecting and freezing the cell pellet. Non-perturbed control cells were treated with drug vehicle (DMSO) for 24 hours (elsewhere called “no-drug control”). Cells were thawed, lysed and protein concentrations were determined by the Bradford assay. Protein concentrations were adjusted to 1–1.5 mg/mL and proteins denatured in 2% SDS for 5 minutes at 95°C. For RPPA, cell lysates were spotted on nitrocellulose-coated slides in Gordon Mills' laboratory at MD Anderson Cancer Center, as described previously [83] and stain with antibodies. Each sample was represented in triplicates originating from three different biological samples (wells). The resulting antibody staining intensities are quantified using the MicroVigene automated RPPA module (VigeneTech, Inc.) and normalized as describes in [83].

#### Resazurin cell viability assay

Cells were grown in 6-well plates and perturbed in the same way as for the RPPA assays. After 72 hr drug treatment, resazurin (Sigma-Aldrich, Catalog # R7017) was added at a final concentration of 44 µM to each well and the fluorescent signals were measured after 1 hr incubation, using 530 nm excitation wavelength and 590 nm emission wavelength. For control wells (0 hr drug treatment), the fluorescent signals were monitored after 4 hr incubation. Standard curves of cell numbers were generated as well to back calculate the cell numbers in different wells. Cell viability measurements at 72 hours are used to ensure the phenotypic responses reached to steady state. Significant phenotypic response is observed as a consequence of changes in relatively early proteomic responses to drug perturbations. Indeed, analysis of cell viability changes at 0, 24, 72 and 120 hours after drug perturbations revealed no significant changes in cell viability between 72 and 120 hours. Conversely, the cell viability response at 24 hours had not reached steady state.

### Methods

#### Setting up belief propagation calculations for network inference

Network models are constructed using the measured proteomic and phenotypic response profiles to drug perturbations as experimental data. The reported network models contain 25 nodes and are trained to protein plus phenotype response profiles from 44 experimental observations. Each measured protein level is log normalized with respect to its measured level at no-drug control condition. For quantification of the activity nodes, see below. The probability distribution for each possible interaction strength in the system is computed using the belief propagation algorithm. In the current implementation, the edge strengths can assume values within the interval [−1, 1] with discrete steps of 0.2. The initial messages are sampled uniformly from a random distribution and the BP algorithm is run until the difference between marginals in consecutive iterations is less than 10^−6^.

The inverse-temperature scaling constant *β* and the complexity penalty term *λ* are the parameters in the BP algorithm that influence expected network connectivity. A systematic approach is taken to ensure the right approximate connectivity in the network models in order to prevent both non-descriptive, sparse models and overfit, highly connected models. Very sparsely connected network models have discontinuities in information flow and low predictive power. Overfit models lack generalizability of predictive power. Empirical representations of signaling networks in the extant literature are fairly sparse, with approximately 1–2 interactions per node. Generally, one has to select *β* and *λ* from a set of BP calculations with systematically varied *β* and *λ* such that a typical BP derived network has a desired connectivity (edges per node). In this work, we explored *β* and *λ* values in the interval (0,5.0], and selected the *β* and *λ* which resulted in the lowest expected error among the representative networks that had a desired connectivity of ∼1.5 edges per node. Representative networks in this case were based on the most probable parameter value for all parameters, each from their respective marginals; we also set a threshold of 0.2 below which parameters were set to zero. The optimal values (*β* = 2, *λ* = 5) are used for the computation of the instantiated solutions in subsequent decimation calculations. For each node. α_i_ is taken as 1 and ε_i_ is estimated from the dynamic range of each proteomic measurement sampled in the biological dataset. The ε_i_ and α_i_ parameters are further optimized with a gradient descent algorithm.

#### Inferring distinct, executable network models of signaling

Distinct model solutions are computed with the BP-guided decimation algorithm. The interaction parameters in each model are further optimized using the Pineda gradient descent algorithm [33], relaxing the assumptions of discrete interaction values and factorized probability distributions (Equation 6d). The gradient descent algorithm also includes optimization of both 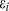 and 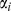 parameters. The optimized models are ranked according to their model errors and the best 100 models are used for simulations and the average network model (Supplementary Figure S6). The average network model is for summary and illustration purposes and is not in itself executable. Instead, predictions are made by simulation of individual, or sets of, instantiated network models.

#### Simulating signaling network models

Each network solution is simulated individually with specific virtual perturbations according to the model Equation 1 until the system reaches its steady state (Supplementary Figure S9) i.e., until no system variable changes in consecutive steps of simulation within machine precision. The DLSODE integration method (ODEPACK) [101] is used in simulations (default settings with, MF = 10, ATOL = 1e-10, RTOL = 1e-20) is used for simulations. Trajectories for the best 100 model solutions yield an ensemble of predicted outcomes in response to *in silico* perturbations.

#### Activity nodes

“Activity node” is a technical term defined within the context of applied perturbations and derived network models (Figure 7-Right, Supplemental Figure- S15). Each activity node quantitatively represents a molecular process or reaction, such as phosphorylation, involving a particular protein (or other signaling molecule) that is affected by a perturbation agent. Since we measure protein and phospho-protein levels and do not directly measure the biochemical activity of any kinase, the activity nodes stand in for the effect of each drug perturbation on the biochemical activity of the drug targets. At a basal level (no perturbation), the quantitative measure of an activity node is equal to the activity level in no-drug control experiments and is set equal to 0 as a reference point. In the presence of an inhibitor molecule affecting a particular activity node, it is calculated based on the influence of the drug on its presumed immediate or downstream target validated with Western blot experiments (Supplementary Figure S4). We demonstrate this with an example quantification of the MEK activity node (aMEK). We measure the strength of a MEK inhibitor by measuring the phosphorylation of its downstream target MAPK1 at residue T202. If the level of MAPK phosphorylation inhibition is 55% compared to a no-drug control experiment, the strength of the inhibition is *u_i_* = log_2_(0.55) = −0.863. Based on the model equations (Equation 1), the value for the activity node is *x_i_* = (β/α) tanh(*u_i_*) = −0.697. The α/β is initially assumed to be 1 and refined by the final gradient descent optimization step. Activity nodes are not allowed to have any upstream regulators except the inhibitor since we do not have any direct measurement of the activity node. All of the activity nodes are quantified using the above procedure and the responses from presumed targets.

#### Mathematical description of perturbations

A constant perturbation (*u_i_*) acting on a particular node *i* (Equation 1) impacts both the time derivative and the final steady state value (*x_i_*) of the perturbed node. The set of interactions and their strengths (*w*_ij_) are independent of the perturbation. As modeled by Equation 1, the dynamic properties and steady state value of node *x*_i_ are a functions of the combination of influence from all upstream nodes (*x_j_*) with nonzero interaction strengths *w*_ij_ and the strength of the perturbation. The perturbation term in Equation 1 models the effect of targeted interventions such as targeted small molecules. The model equation can also incorporate other perturbation forms such as genetic alterations or RNA interference (RNAi). In case of genetic alterations, the impact can be modeled by fixing *x_i_* to a desired value. For example, fixing a particular *x_i_* value to a large negative value (large relative to a given dataset) represents genetic knockdown of the corresponding gene, such as perturbations with RNAi. One may also fix the value of *x_i_* to a positive value to model the impact of amplification of a gene product (e.g., DNA copy number change).

#### Toy data

We generated toy data based on toy network models in order to test the performance of BP against a known set of true interactions. The toy models are generated by first fixing a topology of positive and negative values, which are then assigned a set of real values by drawing from an even distribution between 0 and a maximum strength of 2. The topologies are designed to represent cascade-like hierarchical networks to include parallel chains, feed-forward and feedback motifs. For the analysis focusing on true interactions, the topology is generated with the web-service Gene Network Generator (GeNGe). At the time of this analysis, popular toy data generators such as GeneNetWeaver focus on scale-free like network topologies that are common in gene regulatory networks, but not typical for signal transduction pathways. Given the network model, the data is generated by simulating the model according to Equation 1 in response to external perturbation, until the system reaches a stable steady state. The steady state values for all nodes are recorded in each perturbation condition. In rare cases, the simulations encountered perpetual oscillations and these results are excluded from the final toy data set. These simulations are purely deterministic as no stochasticity is incorporated into the simulations. Noise is added to the data post-simulation. We chose to simulate the dynamics of the toy networks with Equation 1 so as to remove the choice of model equation as a source of error.

## Supporting Information

Code S1 **This directory contains all of the fotran90 source code for performing all of the Belief Propagation, gradient descent and simulation analysis described in this paper.**
(ZIP)

Data Set S1 **This directory contains all of the SKMEL133 perturbation and protein data analyzed in this paper and is properly formatted to work with the provided version of the BP code.**
(ZIP)

Data Set S2 **This directory contains all of the models and data analyzed in Supplemental Figure S17.** The data in this directory is from a separate study of dedifferentiated liposarcoma from the DDLS8817 cell line.
(ZIP)

Figure S1 **Comparison of the correlations between false-positives and true-positives.** The nodes connected by false positive edges inferred by BP tend to be highly correlated (blue dots in Figures S1A and S1B), as analyzed here for two example false positives, each from different false positive motifs. Indeed the falsely connected nodes seem to have stronger correlation than the nodes connected by real edges (red and green dots). This analysis indicates that BP is indeed capturing significant correlations in the data although through false interactions. It also indicates that the edge parameters that BP misses (red and green edges) are somehow compensated by the false positive parameter (blue edges). In the absence of temporal data or more perturbations of the nodes in this sub-network, there may not be sufficient evidence to correctly infer the causal relationship between these connected nodes.
(TIF)

Figure S2 **Alternative higher-order perturbations produce better data for inference with BP than the systematic pair perturbation strategy.** Performance for both datasets is analyzed with mean squared errors (A), recall (B), precision (C), and the total number of BP-inferred interactions (D). The correlations between the training patterns are shown in analyzed in (E) for both perturbation strategies.
(TIF)

Figure S3 **Perfect BP inference with ideal data.** Totally randomized data (A) produces low correlations between training patterns (B). With this maximally informative, BP reproduces all of the true interactions (D), except 2 with zero false positives when compared against the underlying data generating network (C).
(TIF)

Figure S4 **Dose response curve of singe agent drug perturbations.** Dose response curves determined based on Western Blot experiments, shown on top. See table S1 for protein IC40 values, which are calculated with these curves and used in all paired perturbation experiments. Cell viability curve (not shown) was used to estimate PKC inhibitor (Ro-31-7549) IC40.
(TIF)

Figure S5 **Model abstraction in network models of signaling.** A relatively detailed model of biochemical events in PI3K/AKT pathway (i.e. consecutive phosphorylation of AKT at T308 and S473) (reactome.org) and the coarse-grained network model abstraction used in this paper (Red arrows and rectangles). In the model abstraction, PI3K and AKT activity nodes influence the final output of the activation mechanism (i.e. AKTpS473). In princible, we can improve and extend the model with additional measurements such as measurements on PDK1, AKTp308 (circled in gray) etc without altering the model abstraction. Such model improvement requires richer measurements and will lead to a shift from logical to potential physical interactions. Lower left. The PI3K/AKT interactions in the average network model computed with BP-guided decimation.
(TIF)

Figure S6 **6 of the 10 best network models.** Better models have lower cost. Two nodes (horizontal and vertical list) can be connected by a positive (red) or negative (blue) interaction *W_ij_*. Although each model is different in detail, each model represents the data well in the predictive sense. The differences reflect genuine uncertainties of model inference, normally not represented in molecular biology cartoon models. Substantial similarity exists between the top 6 models shown in Figure S6 A–F.
(TIF)

Figure S7 **Predictive power tested by prediction of withheld data.** The 8 distinct heatmaps of withheld (upper right) and predicted (lower right) respone profiles to withheld perturbations (upper left). For quantitative analysis of predictive power, please see Figure 7 in the main manuscript.
(TIF)

Figure S8 **Cumulative predictive power.** The cumulative scatter plot that combines the information in Figure S7 (C = 0.87).
(TIF)

Figure S9 **Sample simulated trajectory.** Pseudo-time trajectory of a single network model when perturbed with an *in silico* perturbation on AKTpS473 at virtual IC50 (i.e. log_2_ ([AKTpS47]_final_/([AKTpS47]_initial_) = −1).
(TIF)

Figure S10 **BP comparison with CoPIA results on mcf7 perturbation data.** The CoPIA network inference method is an older method based on a nested Monte Carlo search, and was previously applied to infer network models of mcf7 breast cancer data. The CoPIA network edges are color-coded based on the existence of those edges in BP inference (A) has many agreeing edges (green). Furthermore, most of the significant BP edges (B) are also significant in the CoPIA models (green). Further analysis is available in Supplementary Text – S4.
(TIF)

Figure S11 **Gaussian model couplings for the most likely cutoff.** The inference with no reweighting produces the highest likelihood estimate (A). The distribution of J and H couplings (B and C, respectively) are strongly centered at zero, demonstrating some discerning power between all possible couplings. Further analysis is available in Supplementary Text – S5.
(TIF)

Figure S12 **Comparison of the most likely Gaussian model couplings and those from BP.** The ROC curve (A) lies firmly above the diagonal line. The set of GGM edges taken from the F1-max cutoff (B) has many agreeing edges with those returned from BP (C). Further analysis is available in Supplementary Text – S5.
(TIF)

Figure S13 **Entropy as a function of the number of bins.** Entropy is normalized by the ratio of total entropy to maximum possible entropy. This analysis is focused on exploring the effects of different discretization strategies (Assumption 1) on the BP inference. Further analysis is available in Supplementary Text –S1.
(TIF)

Figure S14 **Entropy as a function of the number of bins.** This time, entropy is calculated on a collapsed distribution over three regions; aggregate probability mass for negative values, zero values and positive values. Further analysis is available in Supplementary Text-S1.
(TIF)

Figure S15 **Schematic illustration of interpreting model edges and activity nodes.** Direct interactions in our models do not necessarily imply direct biological interactions (A). Rather the separation between any two connected nodes is dependent on those nodes that are included in the model, which is in turn a function of the availability and specificity of assays for various proteins and phospho-proteins. Consider the linear cascade of 5 nodes (A). When intermediate nodes are excluded from the model, for whatever reason, the true direct interactions are also excluded. In their stead, the model will have interactions between the neighboring nodes. In that case, the direct model interactions do no correspond to direct biological interactions. The use of activity nodes may also confuse the interpretation of our model interactions. Activity nodes stand in for the activity of a protein (or phosphorylated protein) that is not directly measureable, as explained in the Materials and Methods section of the main manuscript. It is this activity that is being targeted by a given drug. We can assume that the activity is below basal (x is negative) when the drug is applied. However, in those conditions in which the drug is not applied we have no data or reasonable assumption with which to approximate the activity nodes. Consequently we do not have enough data with which to infer interactions into activity nodes. Activity nodes are therefore restricted to exist as ‘root nodes’ such that they have only outgoing edges (B). We consider the activity nodes to represent the effect of ad rug on the rest of the model nodes.
(TIF)

Figure S16 **Algorithm flow chart.**
(TIF)

Figure S17 **BP comparison against Bayesian Inference of Bayesian network models.** We compare models created from both BP and Bayesian inference (BI) against random and prior knowledge network models. For each class of model, we compare only the top 100 models based on mean squared error after simulation. Both BP and BI (purple and yellow, respectively) outperform both random and prior knowledge models (red and green, respectively). The analysis shows clear separation between all four classes of models, confirming that both BI and BP models are demonstrably informed by the data. Although BP models outperform BI models in this study, it is unclear whether this is generally true, which method predicts biological interactions more accurately, or how low the MSE should be for genuine predictive power to novel perturbations. See Supplementary Text S7 for more information.
(PDF)

Table S1 **This table provides notes curated from the literature for each of the most probable interactions inferred by BP for the melanoma data described in this paper.**
(DOCX)

Text S1 **Discretization of parameter space.** This section focuses on the consequences of different strategies for discretizing the network parameter space.
(DOCX)

Text S2 **Monte Carlo inference.** This section described in detail the Monte Carlo inference method used in the comparison studies in this manuscript.
(DOCX)

Text S3 **Training patterns.** This section contains results from an *in silico* experiment comparing the informative value of data from systematic low order perturbations (drug pairs) against random higher order perturbations (more than two drugs) with respect to BP inference.
(DOCX)

Text S4 **MCF7.** This section examines BP performance on a previously published dataset of perturbation responses in breast cancer from the MCF7 cell line. This section also compares BP to our previously published method for inferring models based on a greedy Monte Carlo search.
(DOCX)

Text S5 **Gaussian graphical models.** This section reports the results of modeling the melanoma data described in this manuscript with Gaussian Graphical Models; a popular and statistically rigorous method for discriminating between direct and indirect correlations between system variables.
(DOCX)

Text S6 **Implementation details.** This section briefly explains some of the more tedious details regarding implementation of the BP algorithm described in this paper.
(DOCX)

Text S7 **Comparison with Bayesian Inference.** This section reports the results of a preliminary comparison of BP models against those inferred from Bayesian Inference. It also compares both class of models to random models and prior knowledge models. The data used in this preliminary study is from similar perturbation experiments on the dedifferentiated liposarcoma cell line DDLS8817.
(DOCX)
