# Supplementary material for: Perturbation Biology: Inferring Signaling Networks in Cellular Systems
Source: PLoS Comput Biol. 2013 Dec 19;9(12):e1003290. doi: 10.1371/journal.pcbi.1003290 (PMC3868523; doi:10.1371/journal.pcbi.1003290)
Supplement: Code S1 — This directory contains all of the fotran90 source code for performing all of the Belief Propagation, gradient descent and simulation analysis described in this paper. (ZIP) [file pcbi.1003290.s001.zip › Code-S1/BeliefPropagation/documentation/README.rtf]

README.Authors: Evan J. Molinelli, Anil Korkut, Chris Sander.Full Belief Propagation model inference and construction from large scale perturbation and measurement data.A brief introduction into compiling the Belief Propagation inference method, preparing the data for inference, and performing the inference.   In the future, additional libraries will give the user option to run simulations.REQUIREMENTS:1) Fortran compiler.  Here we use the gfortran compilerCOMPILING THE CODE1) Change directories to the directory where you have downloaded "BP_version1.0"2) Create the Gradient Descent library >> cd GradientDescent >> gfortran -c *.f >> ar r libPineda.a *.o >> cd ..3) Type 'make' into the command line.  This makes an executable program called "doBP_v1" in the current directory.>> make4) Add this directory to your bash.  5) Change directories to the destination with your data.6) Once you have the 5 necessary input files (see below: PREPARING DATA FOR METHOD USE), type "doBP_v1 < input.txt" to run BP.>> doBP_v1 < input.txt7) Get some coffee.8) A directory is created with the results from this run.9) A sub-directory labeled 'Marginals/' contains all of the marginals in order and labeled with node name.10) The directory contains all decimated models.  PREPARING DATA FOR METHOD USEThe BP algorithm requires 5 input files (1) data.txt (2) pert.txt (3) name.txt (4) prior.txt and (5) input.txt.  The default pipeline is to perform belief propagation on all observed nodes, print the marginal distributions for all nodes, run decimation a user-specified number of times, and perform a gradient descent optimization on all decimated models.data.txt   -> expression, concentration values.pert.txt    -> perturbationsprior.txt   -> list of prior knowledge interactionsname.txt -> information about the observation status and name of each node.input.txt   -> user optionsFORMAT: data.txt, pert.txtThese files should contain N rows (one for each node) and M columns (one for each condition).  Each element in the matrices is a signed floating point number consisting of 5-character spaces (2 significant digits after the decimal point) with a leading white space, taking in total 6 character spaces per number.  e.g.)  -0.49  0.08 -0.26 -0.30 -0.14  0.26 -0.11  0.02 -0.07FORMAT: prior.txtEach row of this file contains a series of 3 space-delimited integers that lists the index of the regulating (upstream) node, the index of the target (downstream) node, and the sign of the interaction (+1 for activating, -1 for inhibiting)e.g)20 3 1This means that the 20th node activates the 3rd node.FORMAT: name.txtThis file contains the information about the model nodes.  Each row contains the information for a single node.  The first entry is a binary integer to indicate if a node is experimentally observed or measured (1) or not (0).  The second entry is the name of the node with no-spaces.e.g)1 AKT_pS4730 aAKTThe unobserved nodes are typically, in this modeling scheme, referred to as 'activity nodes' and represent the direct target of a drug.  For example, aAKT is the activity of AKT, which is down-regulated when an AKT-inhibitor is added to the culture.  We do this instead of treating the phosphorylated state of the target protein (AKT_pS473) as the perturbed node, because the phosphorylation state is not exactly representative of the kinase activity.  Such decisions are up to the user.FORMAT: input.txtThe method has 12 input parameters, 8 of which are user-options.  They are in order:1: SessionID				- a string label for this modeling session.  no spaces.2: Number of Experiments		- integer 3: Number of Nodes		- integer 4: Number of possible weights	- integer (should be an odd number). We recommend 115: Maximum weight value		- floating point number.  We recommend 1.6: BP convergence threshold  	- Exponential number.  We recommend 1.00E-067: Sparsity penalty (lambda)	- floating point number.  User choice (somewhere between 0-5)8: Inverse temperature (beta)	- floating point number.  User choice (somewhere between 0-5)9: Number of prior interactions	- integer.  Must match the number of rows in prior.txt10: Number of observed nodes	- integer.  Must match the number of 1's in name.txt11: Number of models		- integer.  Number of models to create via decimation.  User choice.12: Max number to skip in deci	- integer.  Number of parameter fixes without recalculating marginals.  Safes to use (1), but empiraclly ok to use (5)
