# Supplementary material for: Perturbation Biology: Inferring Signaling Networks in Cellular Systems
Source: PLoS Comput Biol. 2013 Dec 19;9(12):e1003290. doi: 10.1371/journal.pcbi.1003290 (PMC3868523; doi:10.1371/journal.pcbi.1003290)
Supplement: Code S1 — This directory contains all of the fotran90 source code for performing all of the Belief Propagation, gradient descent and simulation analysis described in this paper. (ZIP) [file pcbi.1003290.s001.zip › Code-S1/BP_code_usermanual.docx]

**Belief Propagation for network model inference from perturbation data: Version 1.2**

This software consists of a Fortran90 program for creating network models from perturbation data. The number of nodes in the network is the number of biological entities for which you have measurements, plus any perturbation targets for which no data is available (so-called “activity nodes”). The variable *N* represents the number of nodes in the network. The network is fully parameterized by the square interaction matrix ***W*** of order *N*, where each entry *wij* stands for a directed interaction from node *j* to node *i*. Zero-value entries in ***W*** stand for non-interactions.

The Belief Propagation algorithm, itself, produces only a list of probability distributions for all possible parameters in ***W*** (so-called “marginals”). The domain of the probability distribution is the same for all marginals, and consists of a discrete subset of real valued numbers in a user-specified interval centered at zero. It is imperative that the zero-value be included in the domain. The marginals can be analyzed themselves to look for the most probable interactions. However, these marginals do not constitute a usable model.

Usable models, in this context, are those that can simulate the system response to perturbation in a time-dependent manner. Specifically, the interactions must be incorporated as parameters of a set of coupled Ordinary Differential Equations, which describe the evolution (in time) of the system in response to external forces (perturbations to specific network model nodes) given some initial conditions. In this version, parameters of the ***W*** matrix are trained to data from a single time-point, presumed to represent the stable steady state of the underlying biological system. This is an explicit assumption in the inference formalism, and if this condition is not satisfied in your data, this method version is not suitable for your data.

We use a stochastic sampling method to generate usable models from the marginals. This method, therefore, happens after Belief Propagation. The sampling method picks an *i-j* pair at random, and then fixes the parameter *wij* according to the marginal probability for that entry. However, since the marginals are conditionally dependent on each other, fixing one parameter during the stochastic sampling may change the shape of the other marginals. To account for this, the stochastic sampling method automatically re-computes the marginals after a certain number of stochastic steps. The maximum number of stochastic steps before marginals are re-calculated is a user option (*opt_maxSkip*). The number of models to generate is also a user option (*Ndeci*).

After all of the models are generated, each is subjected to a gradient descent optimization to minimize the difference between simulated data and measured data. The resulting parameters and sum-of-squares error are reported for each model.

Using the models for simulating new conditions is not currently supported. Users may read in the model parameters and run simulations in a language of their choice, as most languages have well tested methods for simulating ODEs, such as ode45 in MATLAB.

**System Requirements**

This code requires the gfortran compiler and has been tested on Linux terminals in Mac OSX (10.5 and above).

**Compiling**

The code base consists of several fortran modules and subroutines, all called from a main file. In this document, terminal prompts will be marked with the dollar sign: $.

Once you have gfortran installed and working on your machine, you should put the code folder somewhere on your machine. Change directories into the code directory. We have included a make file, which will compile all the fortran code with a single ‘make’ command.

$ cd ~/[myPath]/bpCodeDirectory

$ make

This will generate all of the fortran .o and .mod files. This will also generate the binary program file named “doBP_Full”. This will be the command you issue when running the program.

We recommend that you add this directory to your PATH, so that the command can be issued from any other directory, particularly the directory that holds your project data, without having to include the bpCodeDirectory path in the command itself. To do this, simply export the bpCodeDirecotry path to the PATH environment variable in your profile file.

On Macs, using the pico editor:

$ pico ~/.profile

In your editor, type “export PATH=[mypath]/bpCodeDirectory:$PATH”, where obviously you should insert your own path information. IT IS IMPORTANT to include the “:$PATH” so that you do not delete any paths you may have added to your PATH environment variables.

**Running the code**

This section assumes you have added the bpCodeDirectory to your path. It also assumes that you have properly prepared your data for this analysis. In addition to the data files (see “Preparing Your Data”), the method depends on an input file that encodes all of the user’s preferences and some necessary information about the data. The input file consists of 12 lines, each line contains a single value that denotes either necessary information or user options (see “Input File Information by Line”). It is with the entries in this file that you control the output of the program.

Assuming you have all your files and options in your input.txt file, the command to execute the algorithm is simply:

$ doBP_FULL < input.txt

**Input File Information by Line**:

**Line 1 (SessionID)**: An optional Session ID name for the study. It may not contain any spaces. The reason for this option is that you may want to run the Belief Propagation algorithm under many different conditions or different subsets of your data. This line gives you the ability to label each study appropriately.

**Line 2 (Nexpts)**: The number of training patterns you are using in this study. This value must be an integer. The number must also match the number of columns in your data files.

**Line 3 (Nnodes)**: The number of network nodes. This value must be an integer. This number must also match the number of rows in your data files.

**Line 4 (Nwvals)**: The number of discrete values you want in your domain for the resulting parameter probability distributions. This number must be an ODD integer; if not, it will be made odd by the program by adding one. The reason for requiring an odd integer, is to guarantee that the zero-value is included in the domain of the resulting probability distributions.

**Line 5 (MaxWvals)**: The maximum value of the domain. This number must be a decimal number with two decimal places. This number, and the number in line 4, will determine together completely define the domain of your probability distributions. While this number can be any number less than 10, we believe it reasonable to start with a value of 1.00. Future versions will address this parameter in a more robust and statistically motivated way.

**Line 6 (thresh)**: BP termination threshold. This defines the maximum allowed difference between any marginal probability at two consecutive iterations within BP. The number must be in scientific notation. We strongly recommend a value of 1.00E-06, and see no reason to play with this number.

**Line 7 (lambda)**: The complexity or sparseness penalty (the parameter ). This must be a decimal number with two decimal places. We recommend values between 0.00 and 5.00, and is often a parameter that the user will have to experiment with to match their expectation of network connectivity. The larger this value, the fewer the number of non-zero parameters or interactions.

**Line 8 (beta)**: The inverse temperature (the parameter ). This must also be a decimal number with two decimal places. Here, we recommend values between 1.00 and 5.00. The larger this number, the smaller the expected sum-of-squares differences. Values too large may result in over-fitting and fitting to noise in your data.

**Line 9 (Nprior)**: The number of prior knowledge interactions you have in your prior knowledge data file. This number must be an integer. If you do not have any prior knowledge interactions, you should insert the value 0.

**Line 10 (Nobs)**: The number of network nodes for which you have measured data. This number must be an integer. This is the number of non “activity nodes”. Consider the case of a drug that inhibits the activity of MEK, but you do not have an appropriate antibody that corresponds directly to the activity of MEK. In this case, we would include a network node e.g. aMEK, that is an activity node for which we do not have data. We only know when (in which experimental conditions) aMEK is perturbed with the drug.

**Line 11 (Ndeci)**: The number of models you want to generate from the resulting BP marginal. This number must be an integer. If you only want the marginal probabilities, you should enter 0.

**Line 12 (opt_maxSkip)**: The maximum number of times the stochastic network generating method can fix parameters without having to recalculate the marginal probabilities of the unfixed parameters with BP. We have found that a value of 10 is sufficient for speedy network generation with no observable differences against models generated with the more rigorous value of 0.

**Optional Run Flags**

There are some option flags that you can use to change the behavior of the algorithm or output some extra information.

**(-r , --Random**): start the BP run with randomized initial guesses for the marginal probabilities. This implementation of BP is maximally “loopy” and therefore not guaranteed to converge, nor guaranteed to converge to the same unique stable solution. The performance can depend on the initial guesses for the marginals, from which the algorithm iterates. One can run BP many times, each from different initial conditions to more thoroughly explore the solution space.

**(-f, --Factors**): After the BP method converges, this flag will print the factors (the independent probability distributions contributing to a single marginal). For more information about these factors, please refer to ().

**Preparing Your Data**

Your experimental data must be prepared in a fairly specific way in order for it to be properly analyzed by this method. In addition to the input file (described above), the BP algorithm requires 4 files: data.txt, pert.txt, name.txt, prior.txt.

**The data file: data.txt**

This is where your measured data goes for model training. If you wish to partition the data into a training and test set, the test data must be removed from this file. This BP methodology assumes that the range of data values includes both positive and negative numbers; positive numbers correspond to increases in treated vs. no-drug controls, while negative numbers correspond to decreases in treated vs. no-drug controls. We recommend the following mapping from quantified measurement intensities (*I*) to data values (*x*).

The dimensions of the data are *N*-by-*M*, where *N* is the number of network nodes and *M* is the number of treatment conditions. Each number must be separated by a single space. The format for each entry is a signed number with two decimal places. There may be no more than two digits on either side of the decimal point. Positive numbers get no sign, while negative numbers are preceded with a minus sign:

Valid Positive Number: 1.03

Valid Negative Number: -1.03

Invalid Number: 0001.03

Invalid Number: 1.03000

Invalid Number: +1.03

Invalid Number: 100.03

Invalid Number: 1

As mentioned before, not all nodes are measured. For these “activity nodes” you should enter a row of 0.00 values, except for columns that correspond to a condition where a drug was applied that targets that node. For those special cases, you must enter a nonzero number, otherwise BP will never consider an edge involving those activity nodes. Which value to use depends on the dose of the drug in that condition.

Consider two kinds of drug inhibitors: drugs whose direct target is unmeasured, and drugs whose direct target is measured. The latter do not require special treatment, whereas an approximation must be used for the former case.

Data for activity nodes: Drugs for which a direct target is not measurable must be incorporated as activity-nodes. One way is to measure the dose strength of these drugs on the most immediate downstream target for which you can measure, presuming some prior knowledge. If you know the IC-value (the percent of maximal inhibitor concentration) of this drug on the measured downstream target, then you can you can use the following equation to produce a data-value for the activity node in those conditions in which they are applied:

In the above equation, is the value to be used in the data file, and the IC% is the percentage to which the downstream target intensity has reduced relative to untreated conditions.

If you do not have a suitable IC% for a drug concentration, then you must use a guess that scales with dose; smaller doses should have smaller magnitude x-values than larger doses of the same drug.

**The Perturbation File: pert.txt**

This file contains all of the information about the drugs or perturbation agents applied in each experiment. It has the same dimensions and format as the data file. Network nodes (rows) that are not DIRECTLY perturbed will have 0.00 values in all experimental conditions (columns). These entries are the *ui* variables in the model equation, representing the strength of a constant external force on the system through variable *i*.

For those nodes that are directly perturbed with drugs (all activity nodes) should have non-zero values in the columns corresponding to the experimental condition in which they were applied. The relationship between the steady-state value (*xi*) and the perturbation force (*ui*) is given by:

In the above equation, is a constant particular to each analyte (*i*). For the moment, we ignore the contribution from ***Wx***. The inverse function is only defined inside the interval -1 to 1, therefore we use to scale all such that the magnitude of the argument inside the inverse function is necessarily below 1.

Approximating : Let’s assume that across start all conditions, the maximum *x*i for each antibody is only 90% of its maximum possible value. Then we approximate . With this estimate of gamma, the argument inside the inverse tangent is defined. Thus, for each perturbed node in your system we estimate as

**The Names File: name.txt**

This file simply contains the name you would like to use for each node in your model along with a binary integer to indicate if the node is measured in your dataset. The order of nodes in this file must be identical to the order of the nodes in both the perturbation and data file. Each row in the names file corresponds to a single node in your system, and contains an integer and a string separated by a single space. The integer is either 1 or 0; 0 indicates that this node is not measured, while a 1 indicates that it is. Essentially, use 0 for activity nodes and 1 for all other nodes. The string is the name of your node and may not contain any blank spaces.

Example of normal node:

1 pAKT_pS473

Example of activity node (perturbed but not directly measured):
0 aPI3K

**The Prior Information File: prior.txt**

This file contains all of the prior knowledge interactions. These interactions are incorporated as Bayesian priors, and as such are not guaranteed to be included in the final models. Each row represents a single prior knowledge interaction. An interaction is registered with three integers denoting the index of the regulator (upstream) node, the index of the target (downstream) node, and a 1 or -1 to denote a positive or negative interaction. Indices start at 1, not zero. The integers must be separated by a single space.

**The Output**

This software generates file records of the marginal probability distributions, the ‘average’ network and the individual network models drawn from the marginal distributions. All of the output files will be located in an automatically generated subdirectory, the name of which includes the date (YYYYMMDD) and the Session ID that the user specifies in the first line of the input file. If the directory already exists, it is overwritten.

**The Average Network**: The average network is simply the set of average parameters from the BP calculated marginal probabilities. This information is provided in two forms. The file “W_average.txt” is a matrix of decimal numbers that report the average parameter value. Again, *wij*is an interaction from node *j* into node *i*. The other file, “W_average.sif” reports a list of all interactions whose parameter strengths are above 0.25. The interactions display both the node names and the type of interaction. This file is easily opened and visualized with cytoscape.

**The Marginal Probabilities**: The marginal probability files are all located in the “Marginals” subdirectory. The probability distributions are reported for each target node; each file contains N probability distributions for all possible interactions that point into a given node. The marginal probability file name contains three pieces of information: (a) the integer index of the target node, corresponding to the names file; (b) the binary value indicating whether the node was (1) or was not (0) classified as an observed variable, also corresponding to the names file; and (c) the name of the node.

In the present version, the rows and columns are not labeled, which make the results a bit tedious to interpret from these files alone. The rows correspond to all possible incoming edges, and the columns correspond to the normalized probability for each discrete value of the parameter. For example, if the user inputs a maximum weight value of 1, and 3 for the number of weight values, the marginal file will contain 3 columns representing P(w=-1), P(w=0) and P(w=1) for any interaction.

**The Individual Models**: If the user requests individual models in the input.txt file, the code will produce six files for each model. The first two files are Model_X.sif and Model_X.txt, which contain the interaction information generated from the decimation procedure. As with the average network, the .txt file contains the N-by-N matrix of real valued parameters, while the .sif file contains only the non-zero interactions and can be opened in cytoscape for network visualization. The other four files are the results from the gradient descent optimization and are indicated with file names beginning with “result”. The “resulta_X” file contains the degradation constants, while the “resultb_X” file contains the parameters controlling the saturation levels for each model node. The “resultW” file contains the optimized weight matrix and the “resultE” file contains the total sum of squares error of the simulated steady state values against the experimentally measured values (thus only penalizes differences of measured variables and not activity nodes).

**Optional factor file**:

If you run the program with the optional –f or –Factors flag, the program will also produce a single file called factors.txt. Recall that the final marginal probability is the product of many probability distributions, each particular to a single training pattern (data from one perturbation experiment).

The factors file contains all distributions , which encode the “belief” or constraint on a single parameter from the data in any given experimental condition. These distributions can be explored to analyze the informational contribution of each experiment and thereby assist in designing new experiments. It may also point to poorly constrained parameters, for which more information is needed. Each line in the file is a single factor distribution. The line contains an identifier of the factor distribution (the target node, the source node and the experimental index) followed by the normalized probability distribution itself. For a distribution over three parameter values (-1,0,1) you would see something like the example below.

pAKT_pS473/pEGFR_p442/5 0.27 0.60 0.13

pAKT_pS473 is the target node, pEGFR_p442 is the source node and 5 denotes the index of the experiment. The probability distribution for this factor indicates a 27% chance that *wij* is -1, a 60% chance of it being 0 and 13% chance of it being 1.

The file then contains NxNxM lines.
