# Supplementary material for: Perturbation Biology: Inferring Signaling Networks in Cellular Systems
Source: PLoS Comput Biol. 2013 Dec 19;9(12):e1003290. doi: 10.1371/journal.pcbi.1003290 (PMC3868523; doi:10.1371/journal.pcbi.1003290)
Supplement: Code S1 — This directory contains all of the fotran90 source code for performing all of the Belief Propagation, gradient descent and simulation analysis described in this paper. (ZIP) [file pcbi.1003290.s001.zip › Code-S1/TestData/ReadMe.rtf]

This folder contains an example set of files for analysis with the BP method.  This is a slightly out-of-date dataset from the initial melanoma perturbation experiments conducted on MEL-133 cell lines.  The cells were subjected to all single and pair combinations of 8 drugs, inhibiting the kinase activity of:1) MEK122) AKT3) HDAC4) BRAFm5) PKC6) STAT37) mTOR8) PI3KIt includes log-2 normalized measurements of 16 phospho-proteins from the Reverse Phase Protein Array (RPPA) platform.  The normalized measurements are available in the data.txt file and the corresponding perturbations are available in the pert.txt file.  In these datsets, each row is a unique model node and each column is a unique perturbation condition.For the sake of illustration, we have included a single prior knowledge interaction in the prior.txt file.  The input.txt file is a list of the necessary inputs to run the full Belief Propagation pipeline. It includes (in order)1) [string] session id for personal labeling2) [integer] Number of total experiments3) [integer] Number of total nodes (measured or perturbed)4) [integer] Number of allowed parameter assignments5) [double precision: 2 significant digits after decimal] maximum allowable parameter assignment6) [exponential] BP convergence threshold, usually 1.00E-067) [double precision: 2 significant digits after decimal] Sparsity penalty (lambda)8) [double precision: 2 significant digits after decimal] Inverse temperature (beta) fitness penalty9) [integer] Total number of prior knowledge interactions to include10) [integer] Total number of observed/measured nodes (less than or equal to the total number of nodes]11) [integer] Number of models to generate from BP probability distributions12) [integer] Speed of model creation, where 1 is the slowest but most accurate, and 5 is the fastest.
