# Supplementary figures and images for: Perturbation Biology: Inferring Signaling Networks in Cellular Systems
Source: PLoS Comput Biol. 2013 Dec 19;9(12):e1003290. doi: 10.1371/journal.pcbi.1003290 (PMC3868523; doi:10.1371/journal.pcbi.1003290)

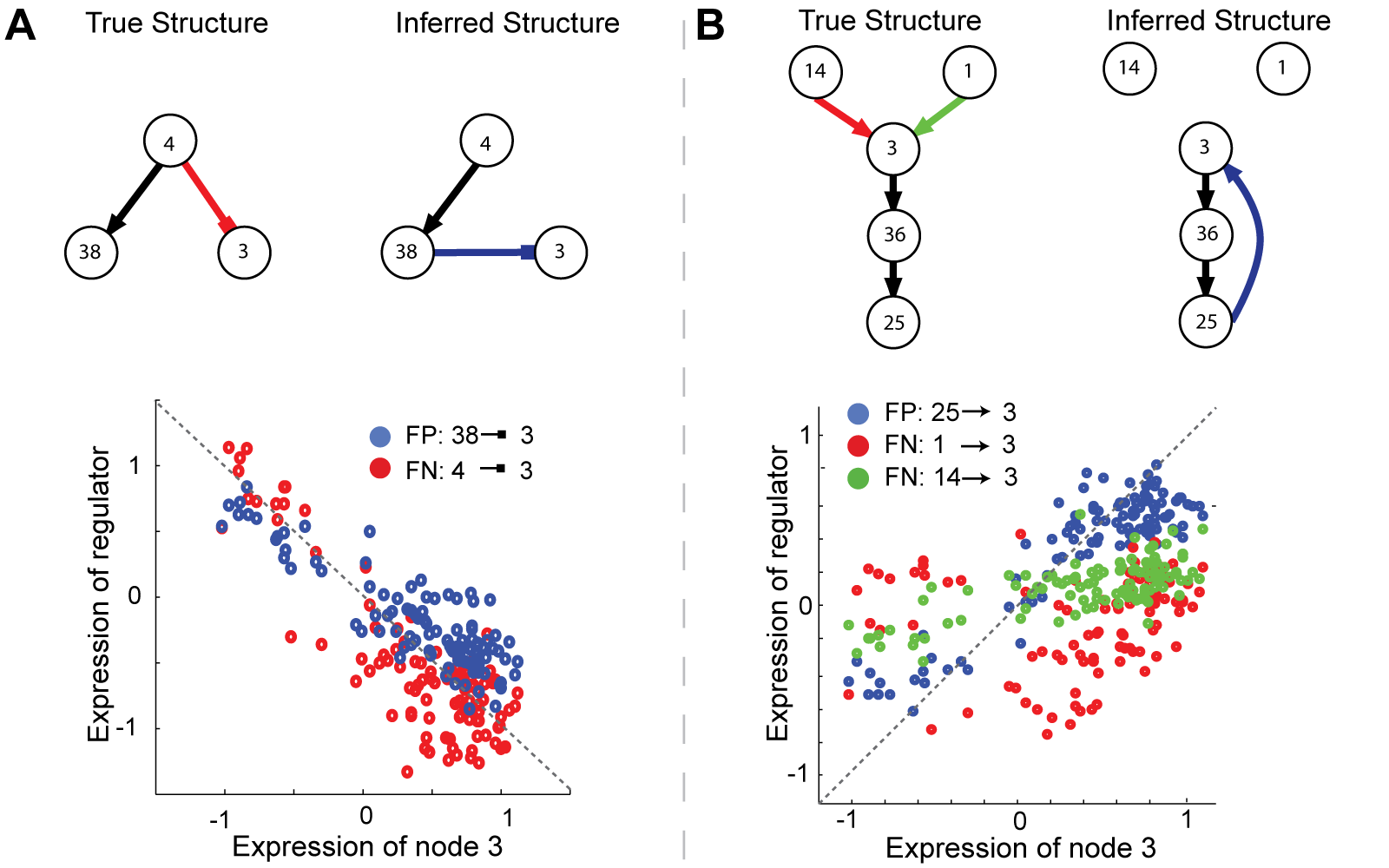

Supplement: Figure S1 — Comparison of the correlations between false-positives and true-positives. The nodes connected by false positive edges inferred by BP tend to be highly correlated (blue dots in Figures S1A and S1B), as analyzed here for two example false positives, each from different false positive motifs. Indeed the falsely connected nodes seem to have stronger correlation than the nodes connected by real edges (red and green dots). This analysis indicates that BP is indeed capturing significant correlations in the data although through false interactions. It also indicates that the edge parameters that BP misses (red and green edges) are somehow compensated by the false positive parameter (blue edges). In the absence of temporal data or more perturbations of the nodes in this sub-network, there may not be sufficient evidence to correctly infer the causal relationship between these connected nodes. (TIF) [file pcbi.1003290.s004.tif]

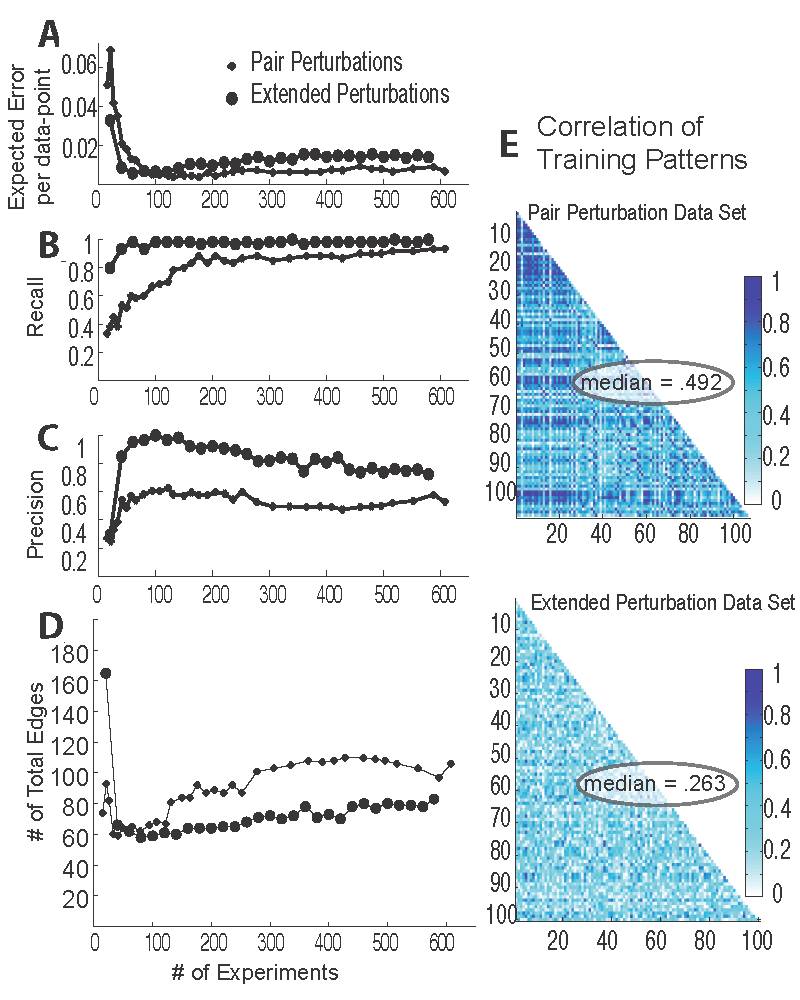

Supplement: Figure S2 — Alternative higher-order perturbations produce better data for inference with BP than the systematic pair perturbation strategy. Performance for both datasets is analyzed with mean squared errors (A), recall (B), precision (C), and the total number of BP-inferred interactions (D). The correlations between the training patterns are shown in analyzed in (E) for both perturbation strategies. (TIF) [file pcbi.1003290.s005.tif]

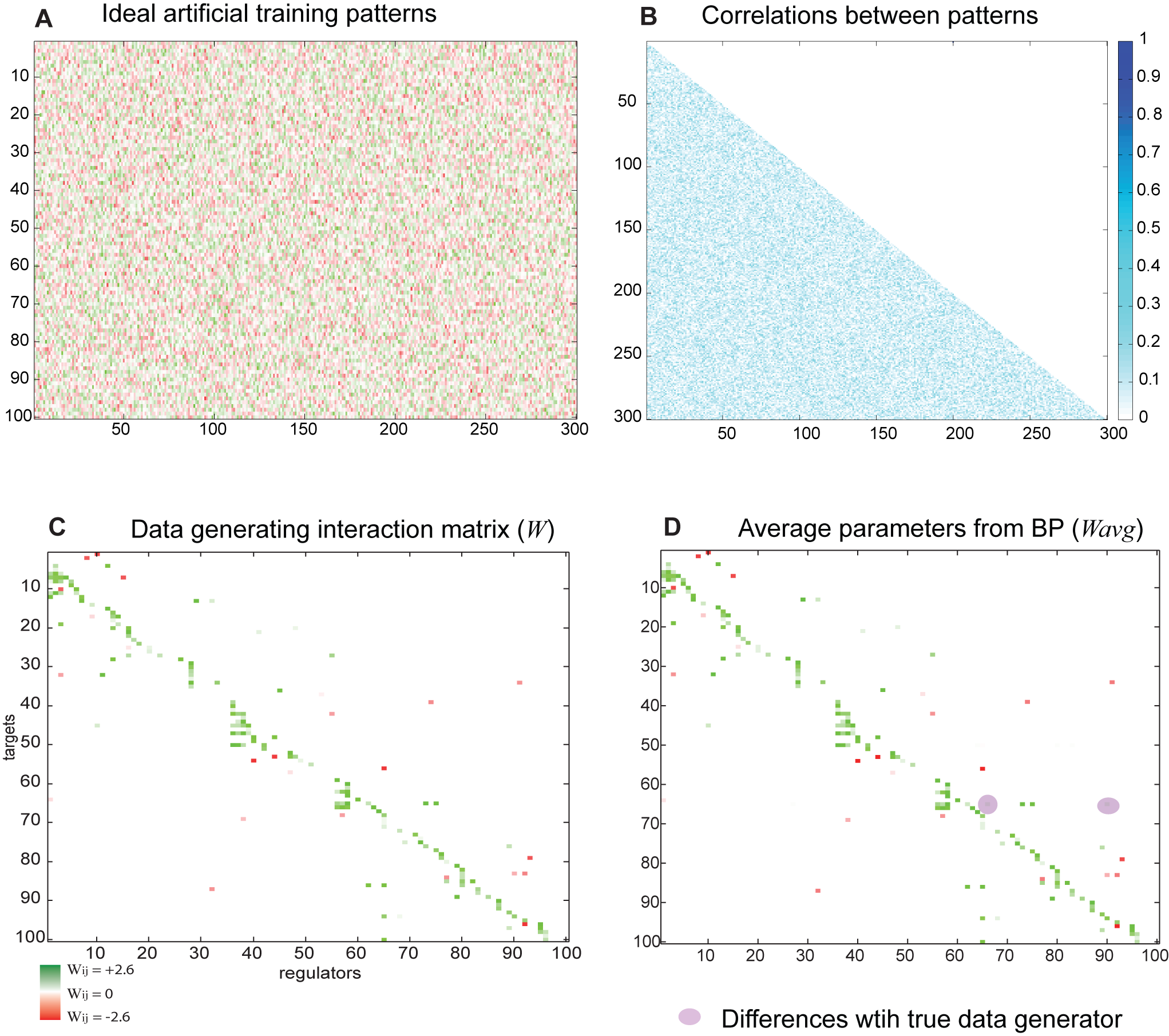

Supplement: Figure S3 — Perfect BP inference with ideal data. Totally randomized data (A) produces low correlations between training patterns (B). With this maximally informative, BP reproduces all of the true interactions (D), except 2 with zero false positives when compared against the underlying data generating network (C). (TIF) [file pcbi.1003290.s006.tif]

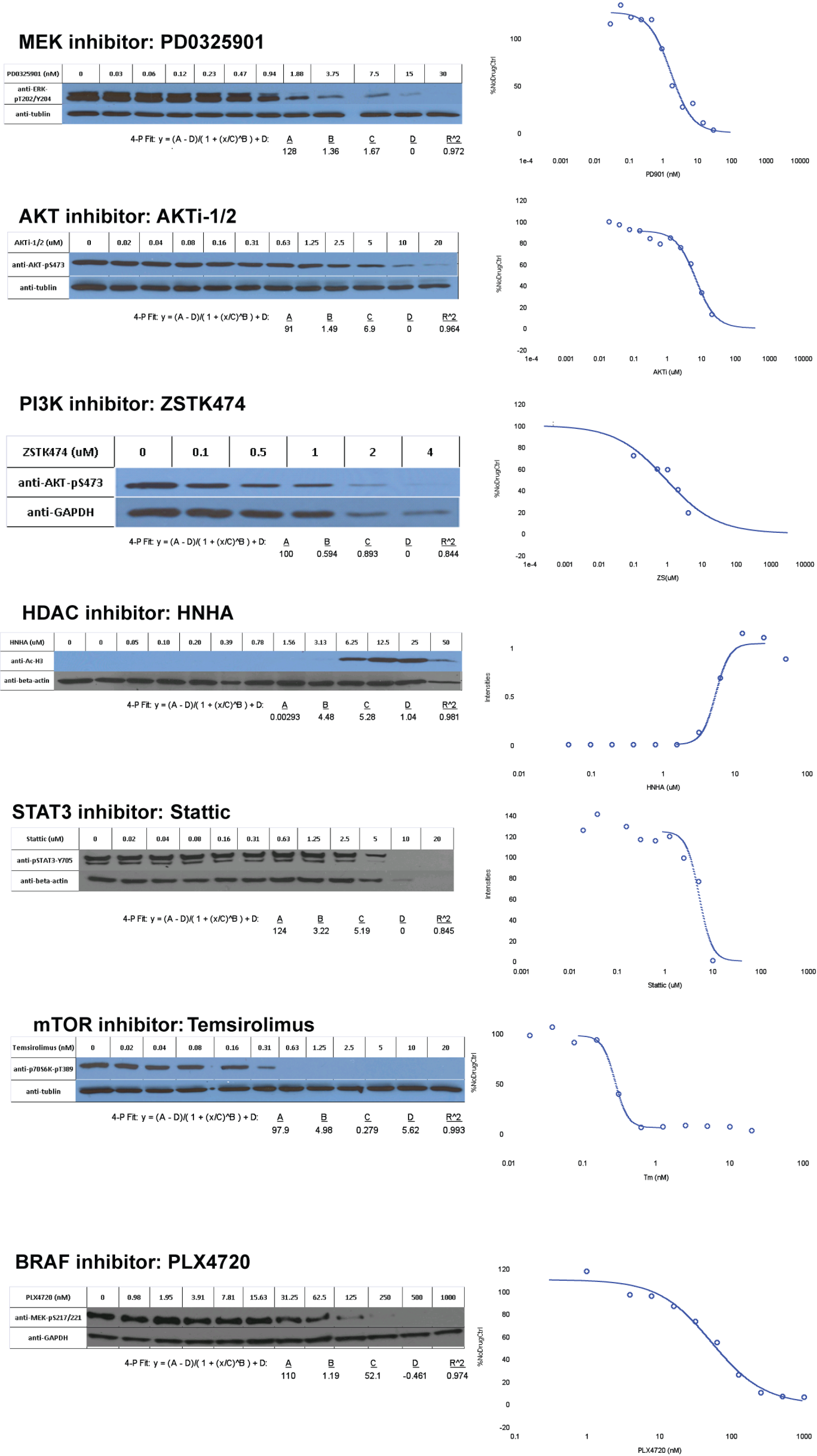

Supplement: Figure S4 — Dose response curve of singe agent drug perturbations. Dose response curves determined based on Western Blot experiments, shown on top. See table S1 for protein IC40 values, which are calculated with these curves and used in all paired perturbation experiments. Cell viability curve (not shown) was used to estimate PKC inhibitor (Ro-31-7549) IC40. (TIF) [file pcbi.1003290.s007.tif]

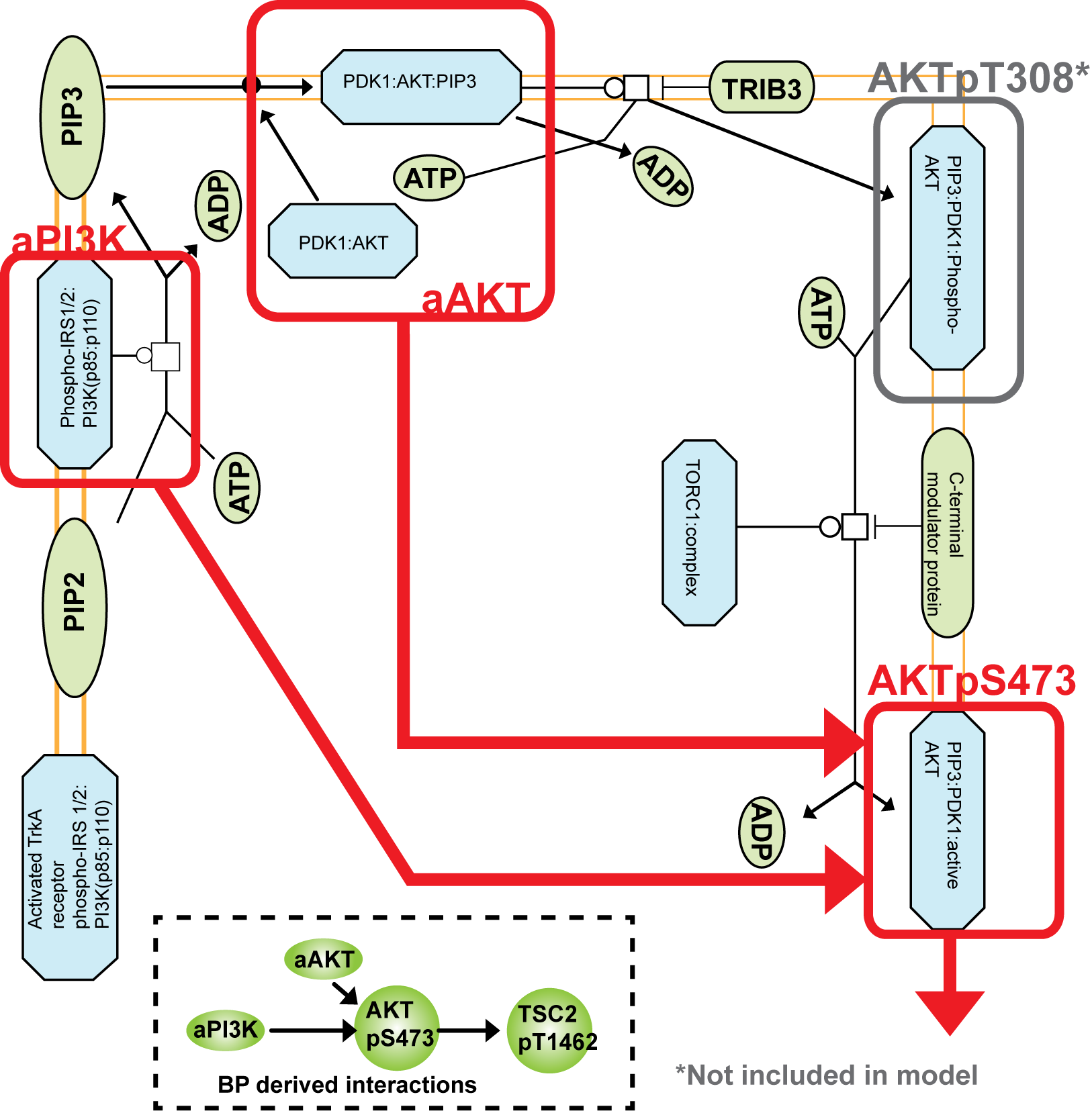

Supplement: Figure S5 — Model abstraction in network models of signaling. A relatively detailed model of biochemical events in PI3K/AKT pathway (i.e. consecutive phosphorylation of AKT at T308 and S473) (reactome.org) and the coarse-grained network model abstraction used in this paper (Red arrows and rectangles). In the model abstraction, PI3K and AKT activity nodes influence the final output of the activation mechanism (i.e. AKTpS473). In princible, we can improve and extend the model with additional measurements such as measurements on PDK1, AKTp308 (circled in gray) etc without altering the model abstraction. Such model improvement requires richer measurements and will lead to a shift from logical to potential physical interactions. Lower left. The PI3K/AKT interactions in the average network model computed with BP-guided decimation. (TIF) [file pcbi.1003290.s008.tif]

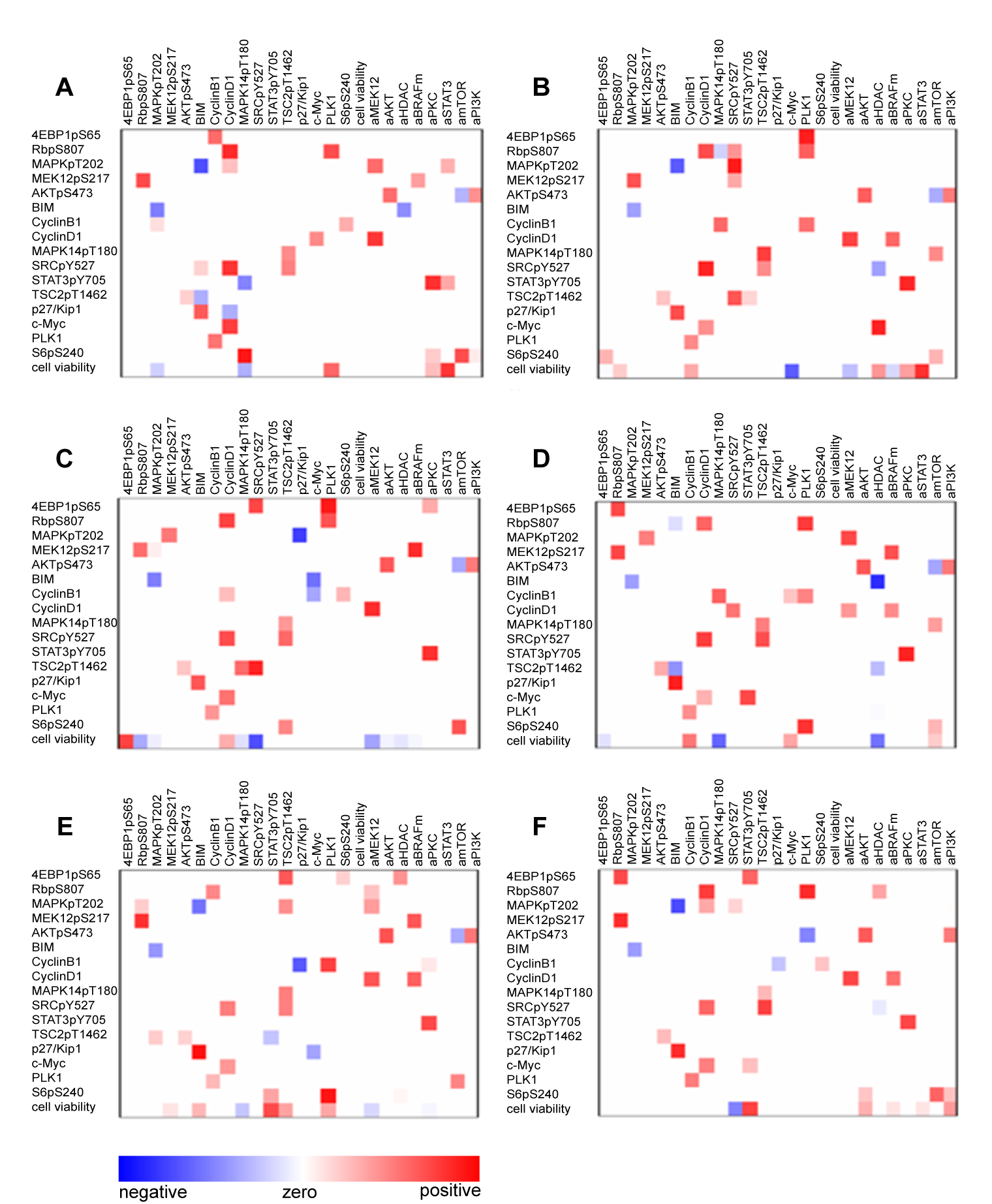

Supplement: Figure S6 — 6 of the 10 best network models. Better models have lower cost. Two nodes (horizontal and vertical list) can be connected by a positive (red) or negative (blue) interaction Wij. Although each model is different in detail, each model represents the data well in the predictive sense. The differences reflect genuine uncertainties of model inference, normally not represented in molecular biology cartoon models. Substantial similarity exists between the top 6 models shown in Figure S6 A–F. (TIF) [file pcbi.1003290.s009.tif]

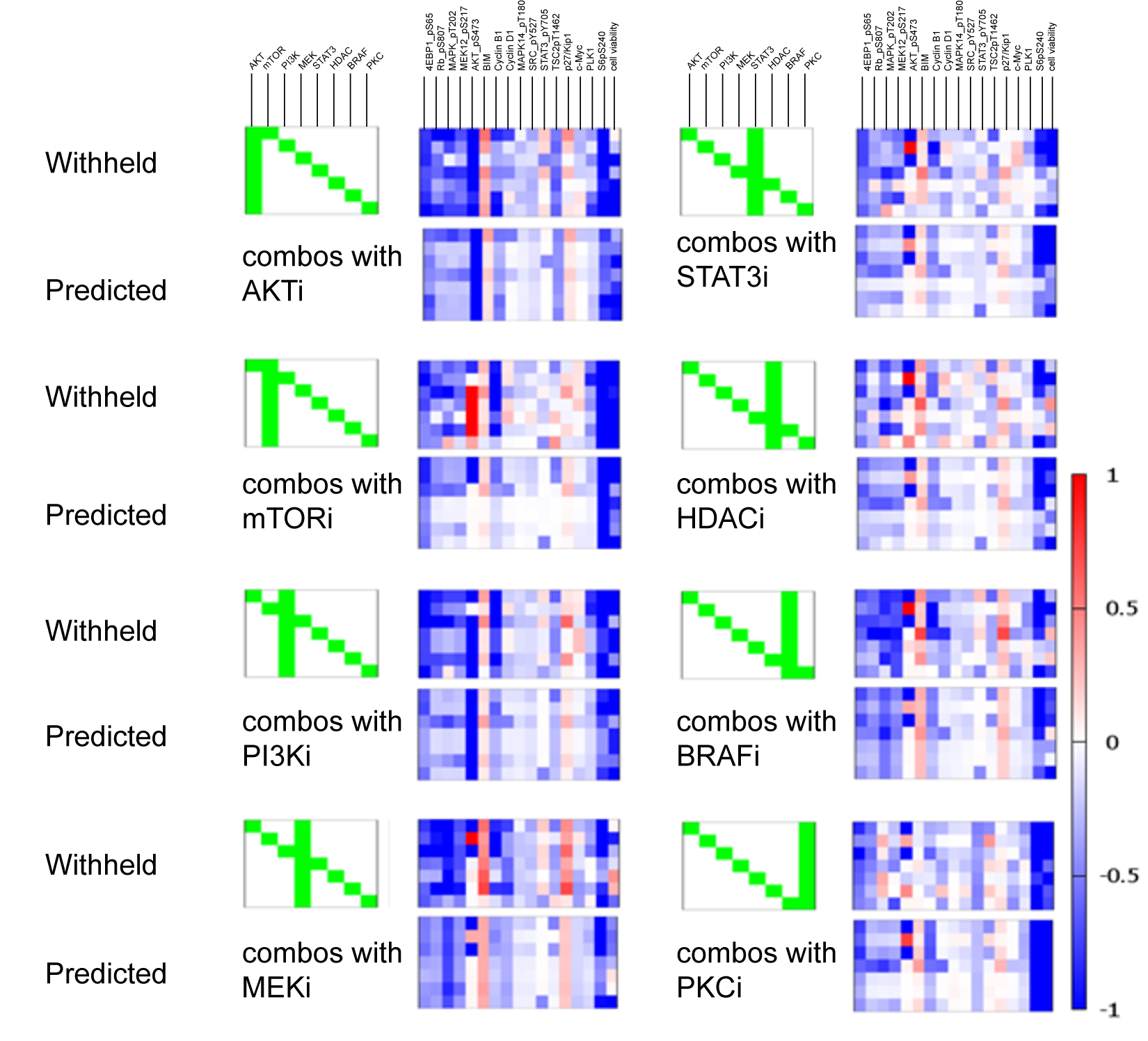

Supplement: Figure S7 — Predictive power tested by prediction of withheld data. The 8 distinct heatmaps of withheld (upper right) and predicted (lower right) respone profiles to withheld perturbations (upper left). For quantitative analysis of predictive power, please see Figure 7 in the main manuscript. (TIF) [file pcbi.1003290.s010.tif]

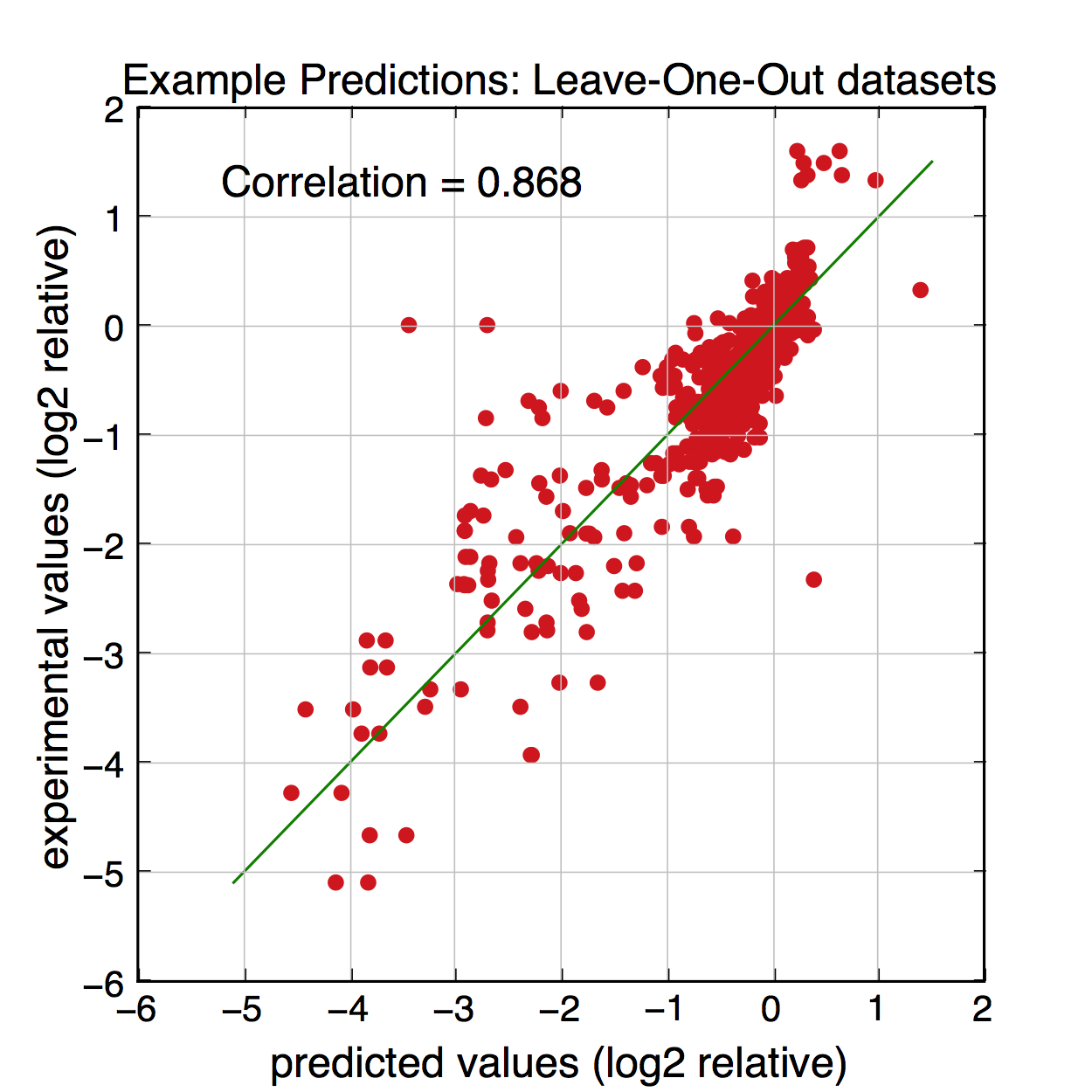

Supplement: Figure S8 — Cumulative predictive power. The cumulative scatter plot that combines the information in Figure S7 (C = 0.87). (TIF) [file pcbi.1003290.s011.tif]

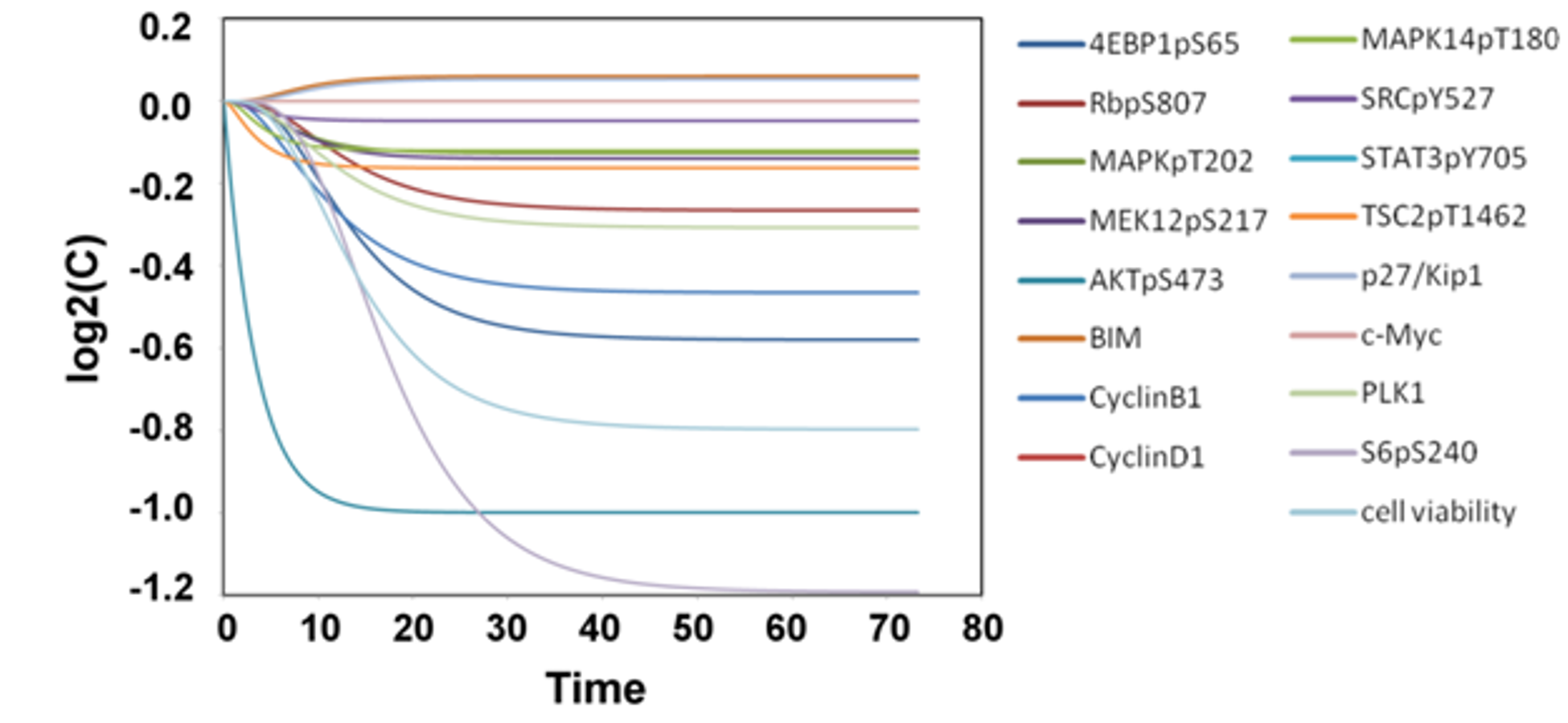

Supplement: Figure S9 — Sample simulated trajectory. Pseudo-time trajectory of a single network model when perturbed with an in silico perturbation on AKTpS473 at virtual IC50 (i.e. log2 ([AKTpS47]final/([AKTpS47]initial) = −1). (TIF) [file pcbi.1003290.s012.tif]

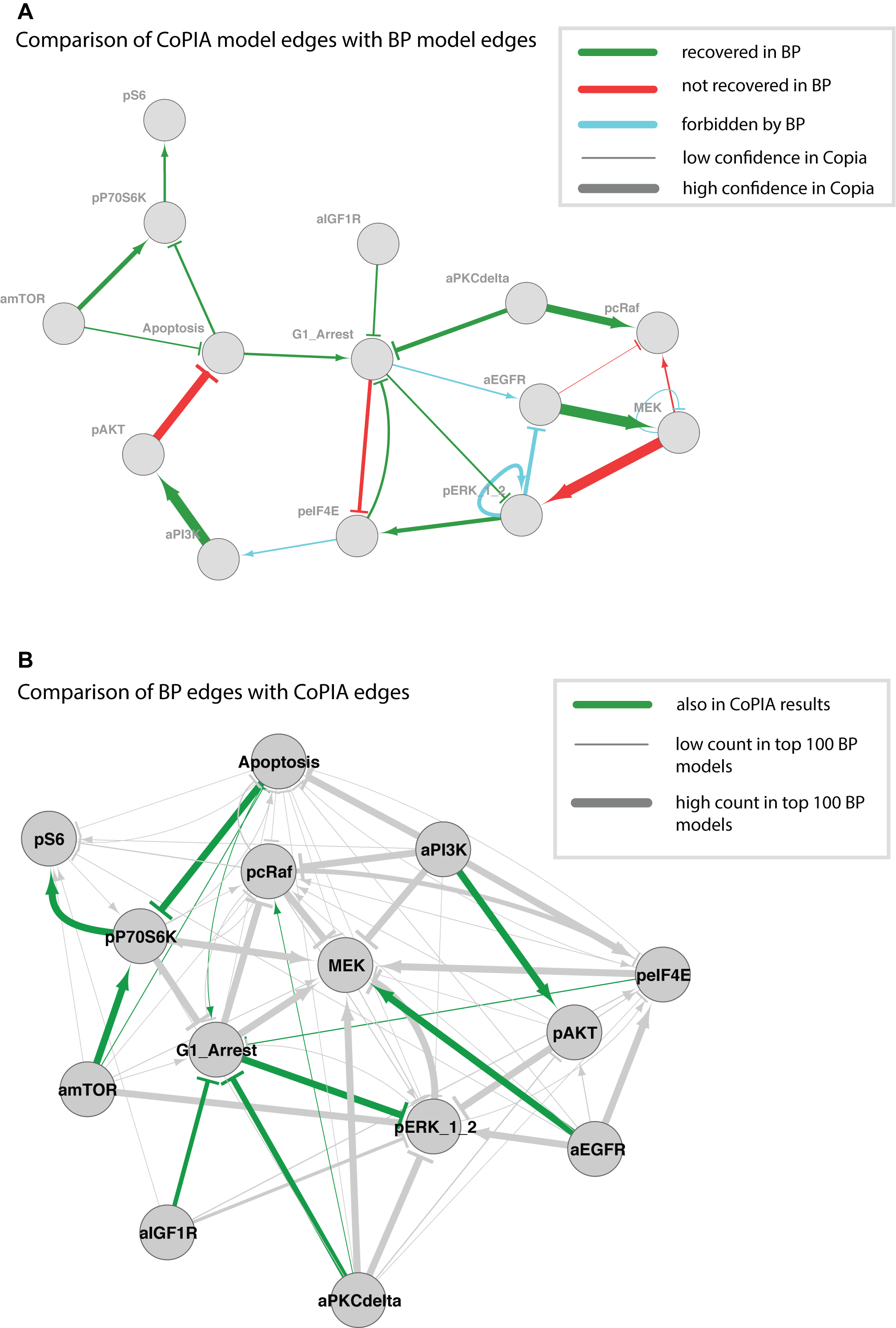

Supplement: Figure S10 — BP comparison with CoPIA results on mcf7 perturbation data. The CoPIA network inference method is an older method based on a nested Monte Carlo search, and was previously applied to infer network models of mcf7 breast cancer data. The CoPIA network edges are color-coded based on the existence of those edges in BP inference (A) has many agreeing edges (green). Furthermore, most of the significant BP edges (B) are also significant in the CoPIA models (green). Further analysis is available in Supplementary Text – S4. (TIF) [file pcbi.1003290.s013.tif]

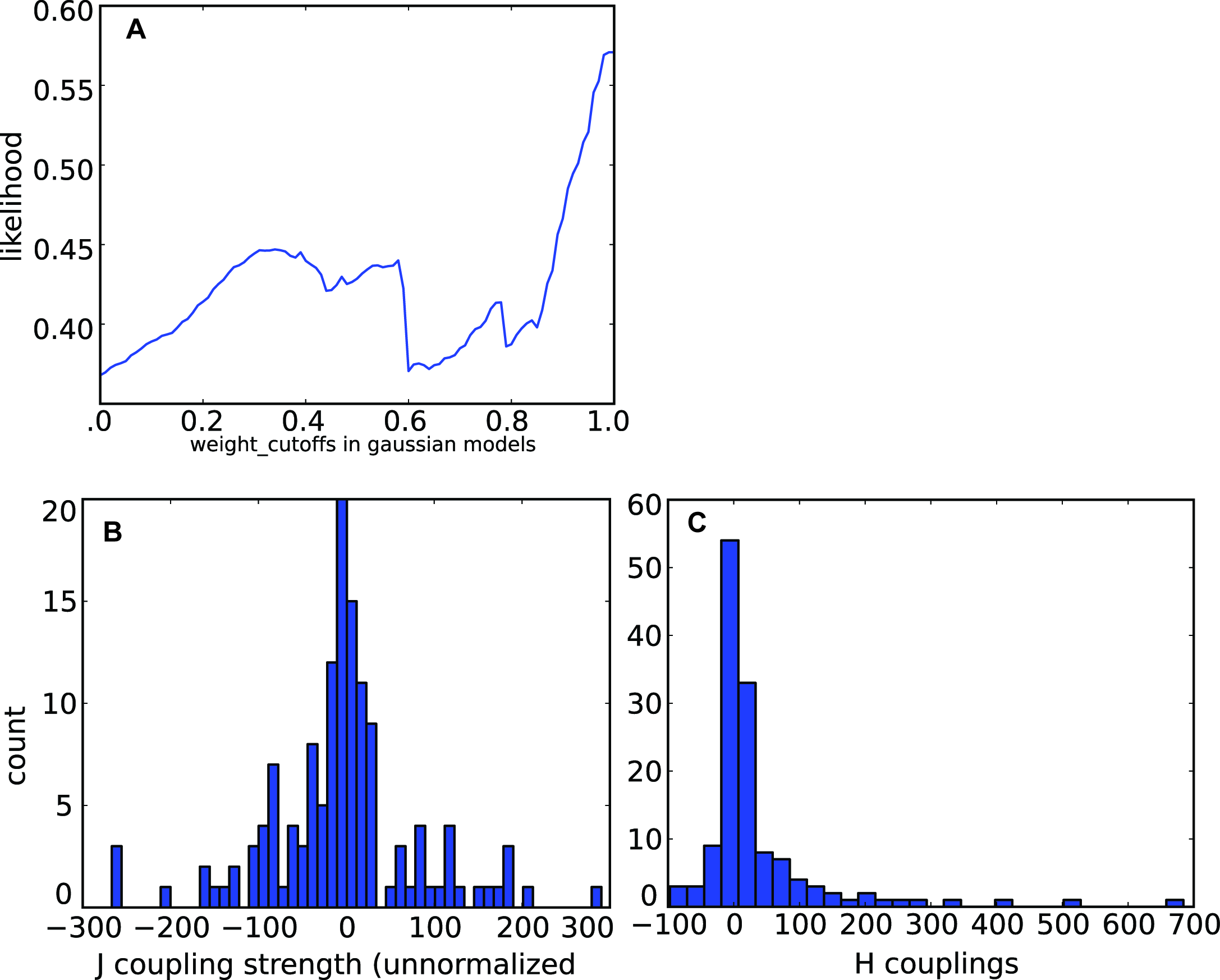

Supplement: Figure S11 — Gaussian model couplings for the most likely cutoff. The inference with no reweighting produces the highest likelihood estimate (A). The distribution of J and H couplings (B and C, respectively) are strongly centered at zero, demonstrating some discerning power between all possible couplings. Further analysis is available in Supplementary Text – S5. (TIF) [file pcbi.1003290.s014.tif]

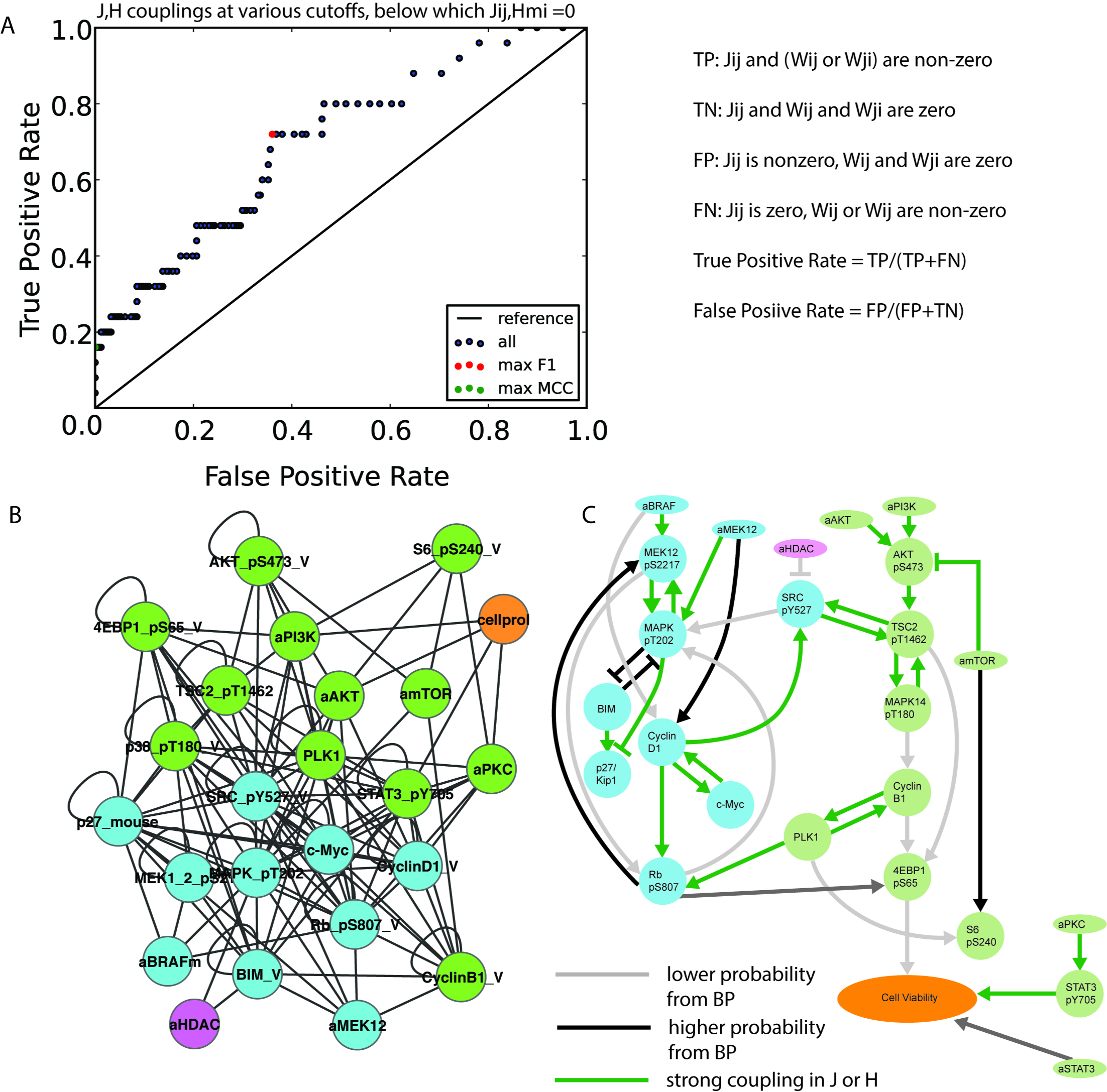

Supplement: Figure S12 — Comparison of the most likely Gaussian model couplings and those from BP. The ROC curve (A) lies firmly above the diagonal line. The set of GGM edges taken from the F1-max cutoff (B) has many agreeing edges with those returned from BP (C). Further analysis is available in Supplementary Text – S5. (TIF) [file pcbi.1003290.s015.tif]

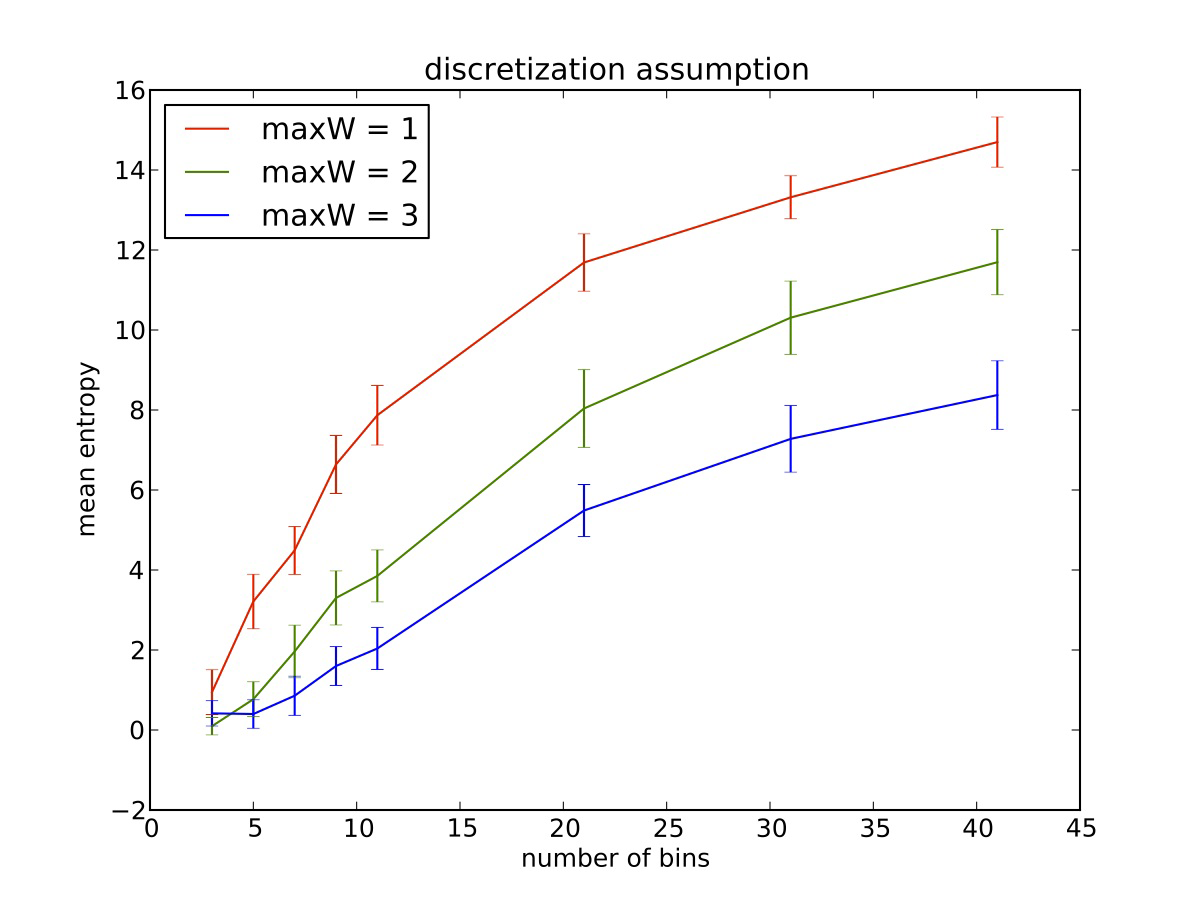

Supplement: Figure S13 — Entropy as a function of the number of bins. Entropy is normalized by the ratio of total entropy to maximum possible entropy. This analysis is focused on exploring the effects of different discretization strategies (Assumption 1) on the BP inference. Further analysis is available in Supplementary Text –S1. (TIF) [file pcbi.1003290.s016.tif]

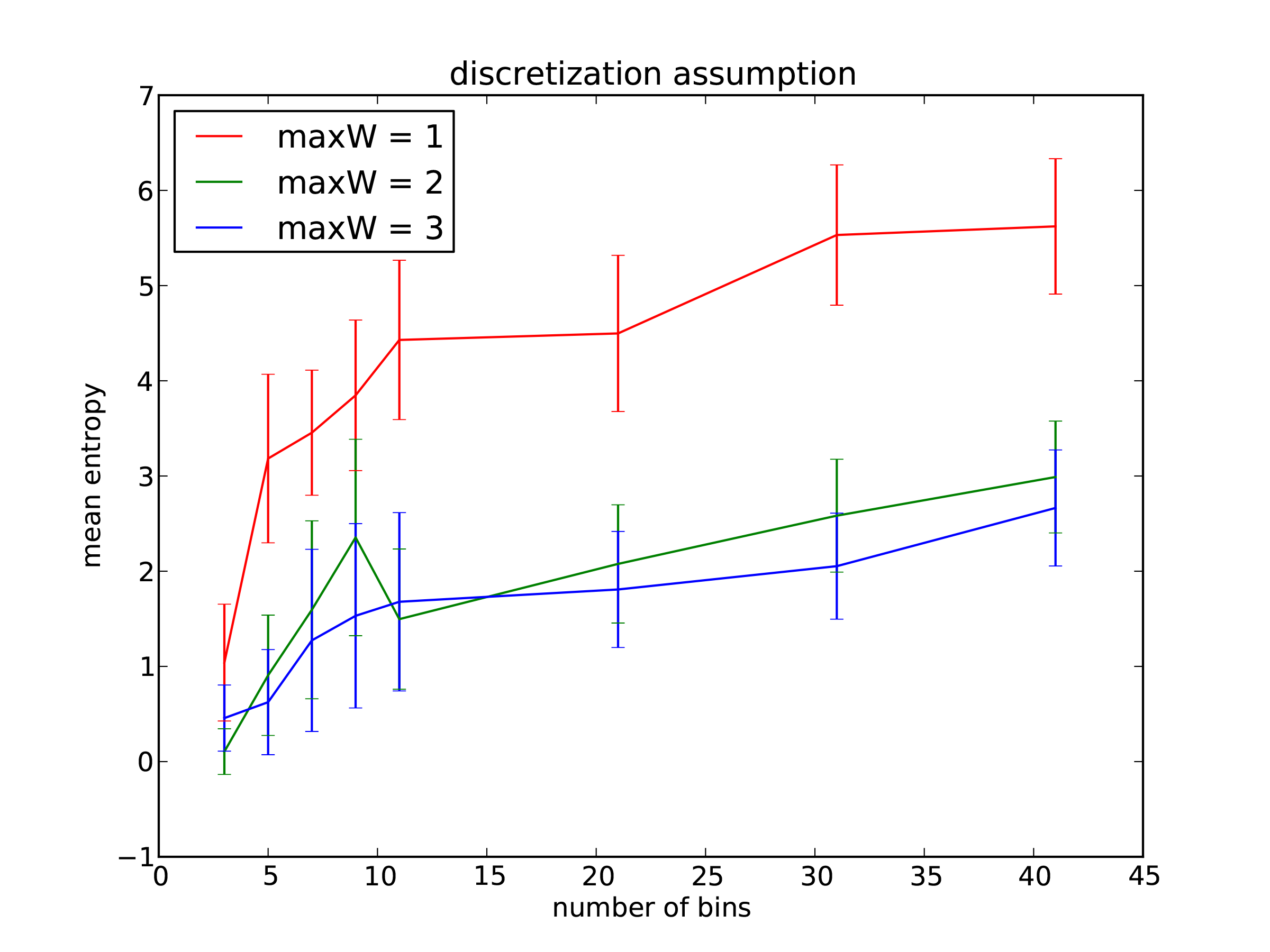

Supplement: Figure S14 — Entropy as a function of the number of bins. This time, entropy is calculated on a collapsed distribution over three regions; aggregate probability mass for negative values, zero values and positive values. Further analysis is available in Supplementary Text-S1. (TIF) [file pcbi.1003290.s017.tif]

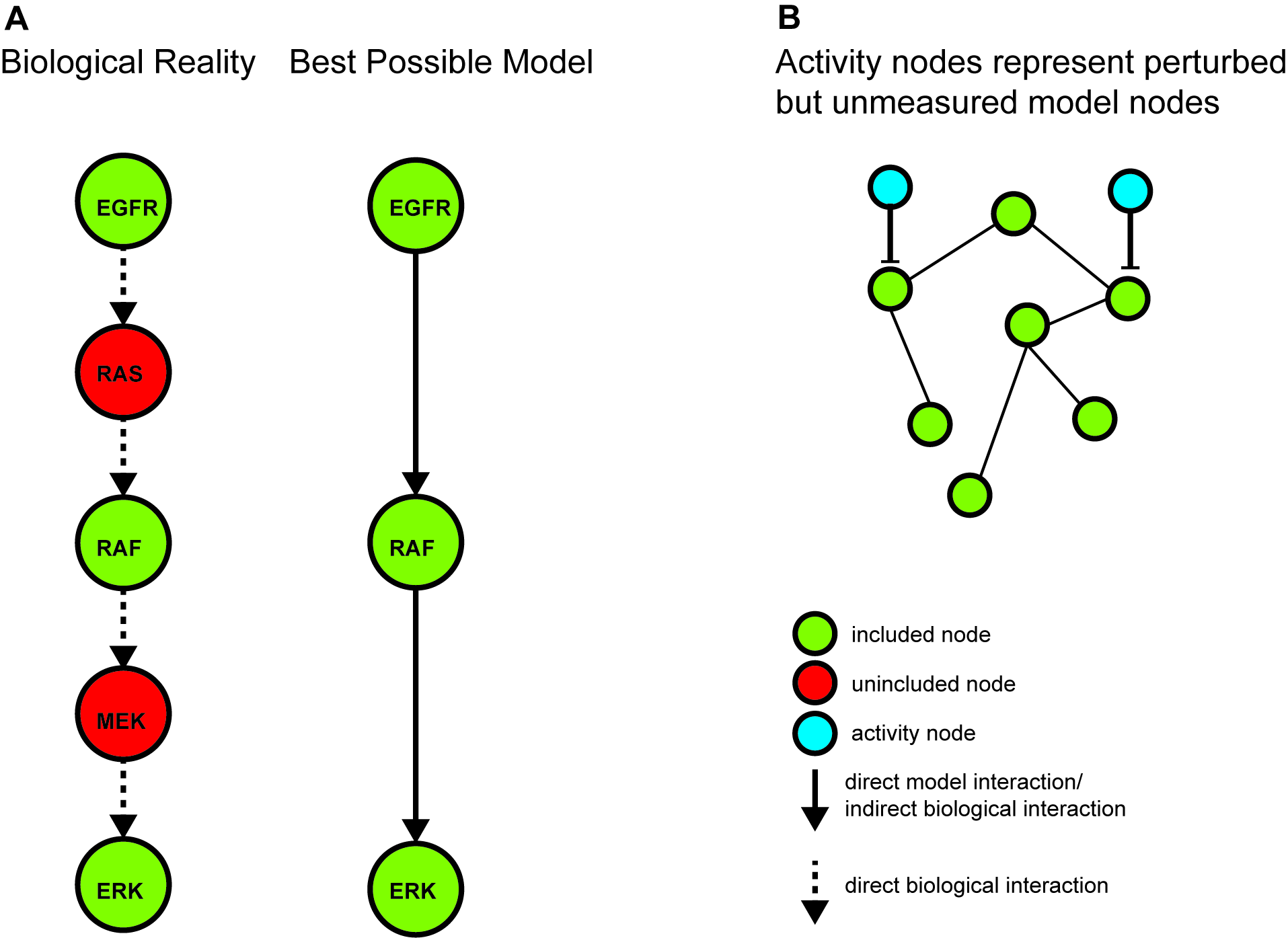

Supplement: Figure S15 — Schematic illustration of interpreting model edges and activity nodes. Direct interactions in our models do not necessarily imply direct biological interactions (A). Rather the separation between any two connected nodes is dependent on those nodes that are included in the model, which is in turn a function of the availability and specificity of assays for various proteins and phospho-proteins. Consider the linear cascade of 5 nodes (A). When intermediate nodes are excluded from the model, for whatever reason, the true direct interactions are also excluded. In their stead, the model will have interactions between the neighboring nodes. In that case, the direct model interactions do no correspond to direct biological interactions. The use of activity nodes may also confuse the interpretation of our model interactions. Activity nodes stand in for the activity of a protein (or phosphorylated protein) that is not directly measureable, as explained in the Materials and Methods section of the main manuscript. It is this activity that is being targeted by a given drug. We can assume that the activity is below basal (x is negative) when the drug is applied. However, in those conditions in which the drug is not applied we have no data or reasonable assumption with which to approximate the activity nodes. Consequently we do not have enough data with which to infer interactions into activity nodes. Activity nodes are therefore restricted to exist as ‘root nodes’ such that they have only outgoing edges (B). We consider the activity nodes to represent the effect of ad rug on the rest of the model nodes. (TIF) [file pcbi.1003290.s018.tif]

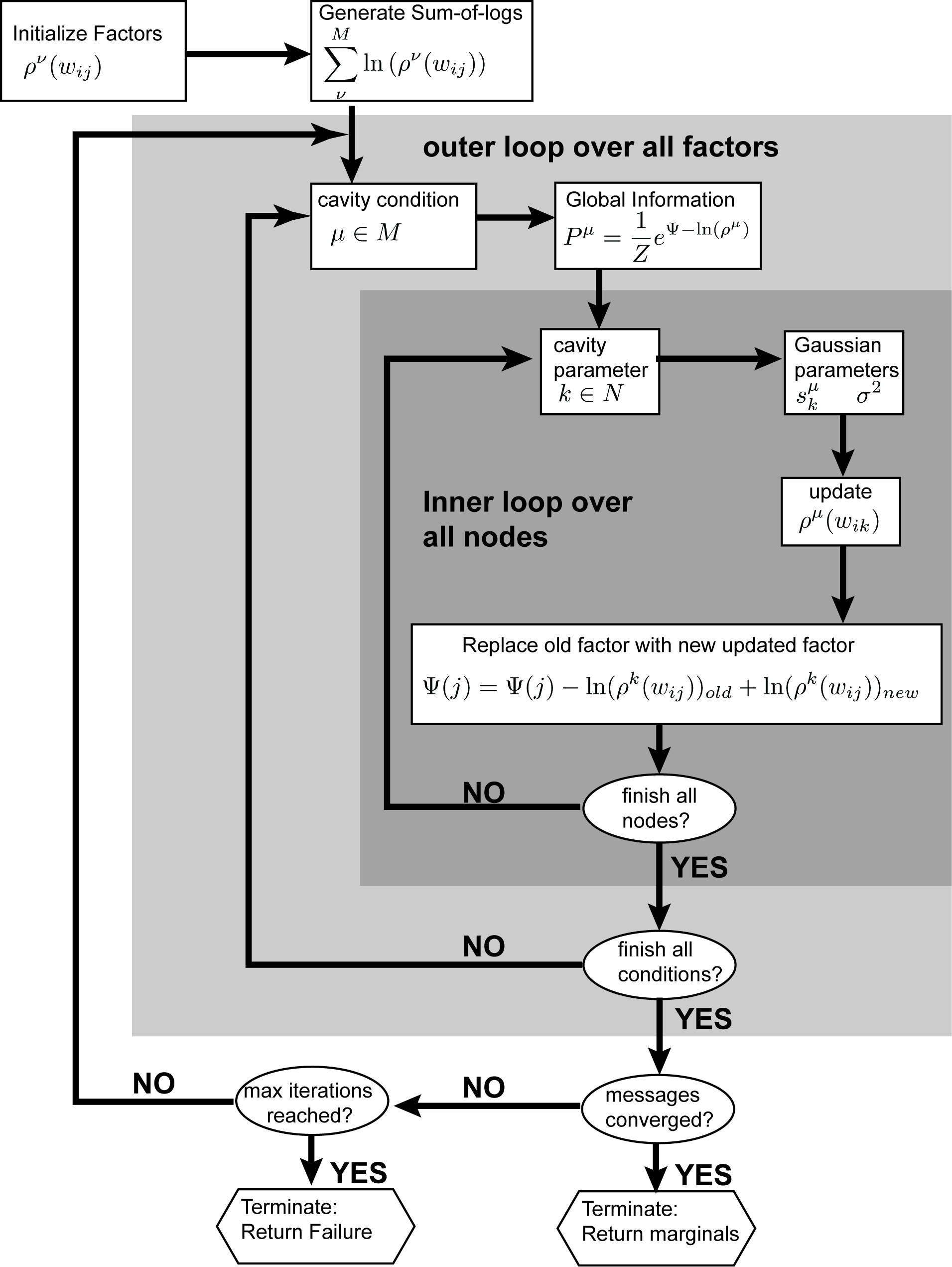

Supplement: Figure S16 — Algorithm flow chart. (TIF) [file pcbi.1003290.s019.tif]

Prior Knowledge Interactions in Modeling

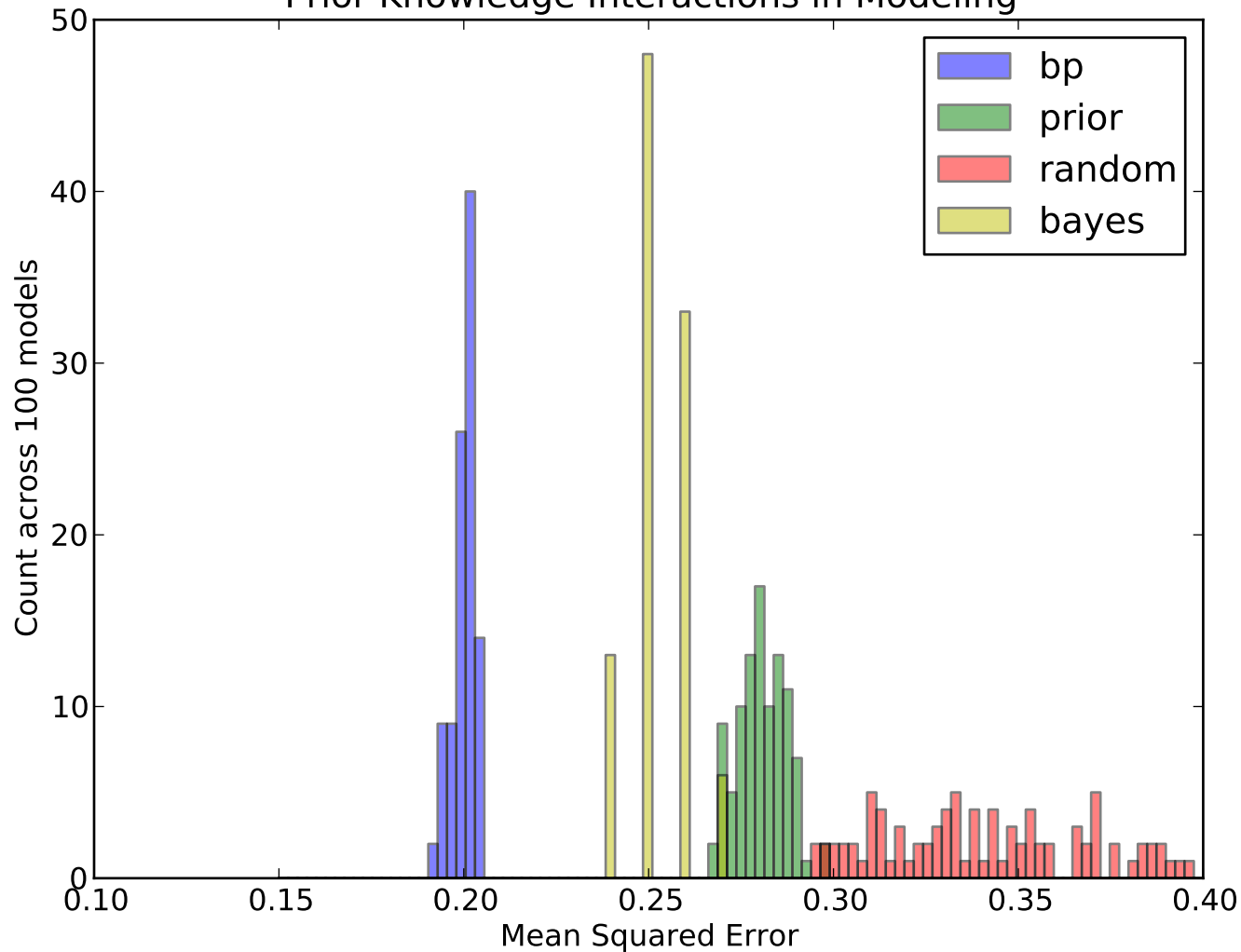

Supplement: Figure S17 — BP comparison against Bayesian Inference of Bayesian network models. We compare models created from both BP and Bayesian inference (BI) against random and prior knowledge network models. For each class of model, we compare only the top 100 models based on mean squared error after simulation. Both BP and BI (purple and yellow, respectively) outperform both random and prior knowledge models (red and green, respectively). The analysis shows clear separation between all four classes of models, confirming that both BI and BP models are demonstrably informed by the data. Although BP models outperform BI models in this study, it is unclear whether this is generally true, which method predicts biological interactions more accurately, or how low the MSE should be for genuine predictive power to novel perturbations. See Supplementary Text S7 for more information. (PDF) [file pcbi.1003290.s020.pdf]
