# Supplementary material for: Perturbation Biology: Inferring Signaling Networks in Cellular Systems
Source: PLoS Comput Biol. 2013 Dec 19;9(12):e1003290. doi: 10.1371/journal.pcbi.1003290 (PMC3868523; doi:10.1371/journal.pcbi.1003290)
Supplement: Table S1 — This table provides notes curated from the literature for each of the most probable interactions inferred by BP for the melanoma data described in this paper. (DOCX) [file pcbi.1003290.s021.docx]

| Modeled Interaction | Confirmed | <Wij> | Notes |
| --- | --- | --- | --- |
| ***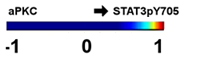*** | Y | 1.00 | PKC mediates the phosphorylation of STAT3 at Y727 following STAT3 phosphorylation at Y705 and dimerization. Thus, this is a potential logical interaction. |
| ***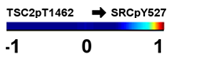*** | N | 0.97 | A predicted interaction. Possible negative feedback acting from PI3K/AKT pathway to upstream SRC. |
| ***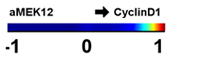*** | Y | 0.94 | Indirect/logical interaction. RAF/MEK/ERK signaling induces Cyclin D1 expression and regulates the transition from G1 to S phase. |
| ***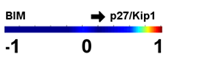*** | N | 0.92 | P27/Kip1 is a tumor suppressor that inhibits cyclin D1. BIM is a proapoptotic protein. The response profiles of the two proteins are highly correlated. The inferred interaction reflects this correlation. Observed edge may be due to a logical interaction. Both proteins are co-regulated by FoxO transcription factor in response to IL-2. |
| ***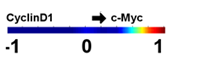*** | Y | 0.89 | The inferred edge between CyclinD1 and c-Myc is bidirectional. c-Myc transcription factor regulates expression of a large spectrum of oncogenic proteins including cyclins. The response profiles of CyclinD1 and c-Myc are highly correlated. However, the inverse regulation is not reported in literature. |
| ***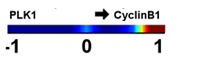*** | Y | 0.87 | PLK1 and CyclinB1 interaction is critical for G2/M transition in cell cycle. This is validated as a direct interaction. |
| ***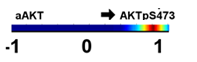*** | Y | 0.86 | Element of PI3K/AKT pathway. aAKT is an activity node, which spesifically corresponds to localization of AKT to membrane through PIP3 binding in our context.After membrane recruiutment, phosphorylation at S473 activates AKT. |
| *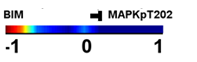* | Y | -0.85 | BIM and MAPK bidirectional interaction is inferred in network models. Multiple studies suggest that proapoptotic activity of BIM is regulated by MAPK through phosphorylation at multiple sites. |
| ***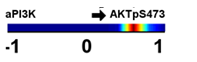*** | Y | 0.69 | Well established logical interaction. PI3K facilitates the membrane localization and phosphorylation of through PIP3 formation. Once localized, AKT is phosphorylated at T308 by PI3K dependent kinase 1 (PDK1). This phosphorylation is followed by a second phosphorylation at S473 by mTOR-rictor complex. |
| ***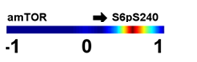*** | Y | 0.68 | Part of PI3K/AKT pathway. amTOR is an activity node. Phosphorylation at S240 activates S6. This is an indirect interaction that takes place through p70S6K (not included in the model.). S56 activation triggers Protein translation. |
| ***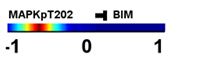*** | Y | -0.66 | MAPK phosphorylates BIM on multiple Serine sites leading to degradation of BIM. |
| ***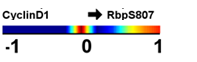*** | Y | 0.62 | Phosphorylation of tumor suppressor protein Rb at S807 by CDK4/6 leads to its inhibition of Rb and G1/S transition. CDK4/6 activation by Cyclin D1 has a major role in inducing Rb phosphorylation. |
| ***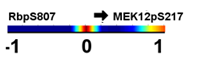*** | N | 0.59 | In canonical RAF/MEK/ERK pathway, an indirect, logical interaction could be defined from MEK to Rb deactivation. However, this interaction has an opposite direction. Few studies point an indirect, bidirectional genetic interaction with Rb and N-RAS, which is upstream of MEK. The observed edge could be a false positive due to the high experimental correlation observed in response profiles of two proteins or may reflect a highly complicated, indirect interaction between MEK and Rb. |
| ***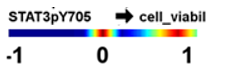*** | Y | 0.57 | PKC regulates STAT3 phosphorylation (see above) and PKC inhibition leads to cell death. The inferred edge reflects the influence of PKC inhibition on cell viability through STAT3 activity. |
| ***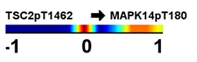*** | N | 0.56 | The reverse interaction is reported as an indirect event. This is a novel prediction and requires experimental validation. |
| ***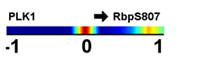*** | Y | 0.54 | PLK1/CyclinB interactions function to activate CDK1 activation, which in turn phosphorylates and activates tumor suppressor Rb. An inverse interaction is reported such that Rb activation leads to the suppression of PLK1 expression. |
| ***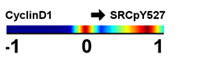*** | ***N*** | 0.54 | This is a possible negative feedback loop acting on Src from Cyclin D1. Src is deactivated when phosphorylated at Y527. Src is upstream of multiple pathways, whose activation lead to increase in Cyclin D1 expression. |
| ***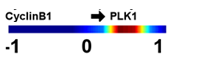*** | Y | 0.52 | CyclinB1-CDK1 and PLK1 activity induce G2/M transition in cell cycle. PLK1 is an important target for anticancer drugs. CyclinB-CDK1 complex and PLK1 regulate the activity of each other. However, the activation of PLK1 by CyclinB1 is most probably an indirect process in mammals and the precise functional relationship between them is highly dependent on biological context. Note that in X. leavis CDK1 directly phosphorylates PLK1 At S340 (not conserved in mammals) and induces its activation. |
| ***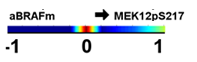*** | Y | 0.50 | Part of canonical BRAF/MEK/ERK pathway. |

Table S1. **Quantitative details and biological significance of inferred interactions**
