# Supplementary material for: Perturbation Biology: Inferring Signaling Networks in Cellular Systems
Source: PLoS Comput Biol. 2013 Dec 19;9(12):e1003290. doi: 10.1371/journal.pcbi.1003290 (PMC3868523; doi:10.1371/journal.pcbi.1003290)
Supplement: Text S1 — Discretization of parameter space. This section focuses on the consequences of different strategies for discretizing the network parameter space. (DOCX) [file pcbi.1003290.s022.docx]

**Text-S1: Effects of the discretization assumption (Assumption 1)**

The result of Assumption 1 in the main text is that BP can only approximate an otherwise continuous distribution (the marginal probability of any single parameter) with a discrete distribution. This begs the question of how to pick the scope (maximum weight magnitude) and the resolution or the number of intermediate values (number of bins). In the manuscript we state that both scope and resolution were picked based on heuristic evidence from extensive numerical studies, from which we conclude that 11 discrete values between -1 and 1 are sufficient to guarantee convergence and recover non-trivial variance in the resulting distributions, which balance exploration of a large solution space against the desire for sufficient constraints on the solution space. In this brief, preliminary study, we wish to share our current progress on investigating this issue more quantitatively. This analysis was done on a single dataset from mcf7 breast cancer cell lines.

The choice of discretization affects three main features of the BP algorithm: convergence, consistency and sensitivity. Convergence is the ability of the BP algorithm to terminate to a stable set of marginal probability distributions. Consistency is the agreement between replicate runs of BP on the same data and depends highly on the initial conditions (initial guess distributions), the order of the updates and the discretization. We have recently discovered that a large number of replicate runs of BP sometimes converge to a much smaller number of unique solutions, each of which is a stable local minima in the error landscape. This is a newly discovered and complex issue and is beyond the scope of this analysis.

Sensitivity concerns the ability to capture probability mass that is concentrated between any two discrete parameter values. Consider the hypothetical example where BP is restricted to consider only parameter values -1, 0, and 1 but where the true weight value is -0.5. This means that the update inside BP can only consider all-on (positive or negative) or all-off parameters. The worry is that the entire marginal might also converge to all-on or all-off distributions, such that one value is given 100% probability and all other values have a probability of 0%. Equally problematic would be the result where the probability distribution splits the probability evenly across -1 and 0, such that half of the samples drawn from this distribution would not have a true interaction and even then would be fixed to a value far from its true value.

To investigate this sensitivity, we track the entropy of the set of marginal probability distributions resulting from different discretization strategies. Roughly, the entropy quantifies the degree of uncertainty captured in a probability distribution. Importantly, it does not say anything about the accuracy of the resulting distribution, which is a separate issue. Distributions with absolute certainty have zero entropy, while maximum entropy describes even distributions where all weight values are considered equally likely. We consider three different scopes with maximum weight magnitudes of 1, 2 and 3. For each scope, we consider 8 different resolutions, where the range is divided into equally spaced bins. We consider a minimum of 3 bins covering one negative, one positive and one zero-valued weight.

The metric of interest is the sum of the entropies of all BP-calculated probability distributions. We calculate the entropy for each set of marginal distributions from 100 independent runs of BP to get a sense of the variability of entropies across replicates. One consideration is the choice of normalization, since the maximum entropy (entropy of even distributions) increases with the number of bins. One choice is to normalize the entropy by the maximum entropy for that bin number (Figure S13). Yet another choice is to collapse a distribution into a ternary distribution of negative, positive and zero values, by calculating the aggregate probability mass over all negative, positive and zero values, respectively (Figure S14). These collapsed ternary distributions are fundamentally different from the BP-calculated distributions over only three values in that prior to collapse, the BP considered a larger number of possible values and could capture intermediate positive and negative values.

In both Figures S13 and S14, discretizing over 3 bins produces distributions with low entropies near zero, confirming the suspicion that such discretization tends to yield all-or-nothing marginal probability distributions. These distributions are dangerous since they likely over constrain the search space. Furthermore, true non-zero values are given zero probability and thus would never appear in models drawn from these distributions. In short, appropriate uncertainty is not capture in these distributions.

Both normalization strategies show increase in the entropies with increasing the number of bins. Both curves are sub-linear and appear to saturate at increasing bins. There are two major differences in the features of these normalizations. First, is that the entropies over the ternary distributions saturate faster and most of the increase in this entropy happens between 3 and 11 bins. Conversely, the entropy increases substantially all the way up to 41 bins in Figure S13. Without supplementing this analysis with comparisons of marginal quality (in terms of accuracy or predictive power), it is unclear which discretization strategy is optimal and whether there is any useful advantage to higher resolutions beyond 11 bins.

The second major difference between Figures S13 and S14 are the vertical shifts in the curves between different scopes. This phenomenon in S13 is easily explained by the fact that increasing the scope for a constant bin number results in a proportional increase in distance between discrete weight values. Assuming that two distributions have identical means and variances, then widening the scope without a changing the number of bins, would concentrate the probability mass over fewer bins resulting in a lower entropy. This is likely why we see uniform decrease in entropies in Figure S13 for increasing scopes. This artifact is not applicable for the entropy calculation in S14. In Figure S14 we notice the vertical shift from high entropies with a scope of 1 to lower entropy curves that are very similar for scope of 2 and 3. The reason for this shift is not yet solved. One hypothesis is that there is more uncertainty when the scope is insufficiently small. If the scope were too small, then the largest magnitude value is insufficient for some parameters although we hope that those values be given a non-zero probability. Because of this inefficiency, BP must explore alternative parameters to compensate for the unexplained variance between observed and expected. This hypothesis also explains why the entropy curves are similar for both scopes of 2 and 3. If the true scope were between 1 and 2, then any scope above 2 would cover superfluous parameter values. And since the entropy in Figure S14 is only a function of the aggregate probabilities of all negative, positive and zero-valued weights, then the entropy is agnostic to the distance between weight values, unlike the entropies in Figure S13.

These results are inconclusive and communicate only our progress towards achieving an objective strategy for picking an optimal scope and discretization that maximizes convergence rates and is sufficiently sensitive to balance the desire to explore parameter space against the attempt to restrict the parameter space to the most likely parameters.
