# Supplementary material for: Perturbation Biology: Inferring Signaling Networks in Cellular Systems
Source: PLoS Comput Biol. 2013 Dec 19;9(12):e1003290. doi: 10.1371/journal.pcbi.1003290 (PMC3868523; doi:10.1371/journal.pcbi.1003290)
Supplement: Text S2 — Monte Carlo inference. This section described in detail the Monte Carlo inference method used in the comparison studies in this manuscript. (DOCX) [file pcbi.1003290.s023.docx]

**Text-S2:** **The Monte Carlo Algorithm employed in this manuscript**.

We compare Belief Propagation (BP) against a standard Monte Carlo (MC) algorithm, as MC is ubiquitous and well understood throughout the literature. MC also has its roots in statistical physics. If MC is run through enough iterations, it is guaranteed to ultimately traverse the entire solution space and is in this, respect, a greed search algorithm. Such exhaustive searches are prohibitively long and thus an adequate termination criterion determines the run time of the MC. Marginal probability distributions are drawn from MC solutions by summing the frequency each parameter appears and weighting it by its likelihood. BP is guaranteed to converge to the exact marginal in tree-like relationships (no loops) between variables and constraints. However, since our problem is fully-connected, each variable is connected to all constraints, BP is not guaranteed to converge to the true marginal and is only an approximation. Since BP is a method to approximate the same marginal probabilities, MC is a suitable candidate for comparison.

MC relies on assumptions 1 and 2, as introduced in the main document. This is necessary, since it is imperative to compare both BP and MC such that they are tasked to solve the same problem with the same likelihood function over an identically sized search space. On the other hand, neither assumptions 3 nor 4 are not applicable to the MC algorithm, as MC is a search over discrete parameter configurations. Therefore, comparing BP results against MC results might isolate the effects of assumptions 1 and 2, collectively, on the quality. It should be noted that letting MC run for longer times would improve MC performance.

We encode a termination criterion that stops when the algorithm has found a strongly attracting minimum for a pre-fixed number of iterations. Our MC algorithm keeps track of the top 1,000 configurations it has encountered, as evaluated by the log-likelihood (or cost function) in Equation 6b. MC traverses solution space by drawing each parameter from a zero-heavy distribution, where all non-zero weights are given equal probability. When the current configuration has lower cost than the previous configuration, it is accepted and appended to the list of the top 1000 configurations, while the highest cost configuration in the list of the top 1000 is expelled. In order to prevent the search from getting stuck in a weakly attracting local-minimum, we consider accepting higher cost configurations with probability:

$$P\left( keep Wnew \right)= \frac{e^{C(W_{new})}}{e^{C(W_{current})}}$$

The algorithm terminates when the list of the top 1000 configurations does not change after 1000 iterations.
