# Supplementary material for: Perturbation Biology: Inferring Signaling Networks in Cellular Systems
Source: PLoS Comput Biol. 2013 Dec 19;9(12):e1003290. doi: 10.1371/journal.pcbi.1003290 (PMC3868523; doi:10.1371/journal.pcbi.1003290)
Supplement: Text S3 — Training patterns. This section contains results from an in silico experiment comparing the informative value of data from systematic low order perturbations (drug pairs) against random higher order perturbations (more than two drugs) with respect to BP inference. (DOCX) [file pcbi.1003290.s024.docx]

**Text-S3: BP accuracy on better data**

In this short analysis, we wanted to investigate the effect that perturbation strategy has on the performance of the BP data. Currently, we use a set of drugs targeting a subset of all model variables and apply them individually and in all possible pairs to generate the training data (Pair Perturbation Data). We wanted to compare this strategy against so-called higher order perturbations, where many nodes are simultaneously perturbed in a single experiment (Extended Perturbation Data). In each experiment, up to five nodes are randomly selected for perturbation. Furthermore, the set of targetable nodes are restricted to the same set as for the pair perturbation data. This study is performed entirely on a single toy model (the same 40 node network as that analyzed in the main manuscript), simulated to steady state for each perturbation condition described above. The steady state responses were subsequently used to infer the interactions of the underlying data-generating network.

The training patterns that emerged from the pair perturbation strategy tend to be highly correlated, such that most pair combinations provided little unique information. Conversely, the extended perturbation data set produces less correlated training patterns (Figure S2E,). It is clear that the training patterns with higher median correlation would have less information than the training patterns with lower correlation.

We subsequently evaluated recall and precision (Figure S2B,C respectively) of the BP models trained to the two data sets. The BP models from the extended data set reach a higher recall and do so faster than for the pair perturbation data. Similarly, the precision curve for the extended data set lies consistently much higher than for the pair perturbation set. Finally, as we increase the number of experiments from the pair perturbation data set (by allowing additional nodes to be targeted) we definitely see that we get new information about the true interactions since the recall curve goes up. However, the novel information per additional experiment is fairly low and yields networks with increasing numbers of interactions. These three results confirm that using BP against more informative data produces superior network model, and that different strategies of perturbing the system yield training patterns with different information.

Although we see similar correlations between pair perturbation datasets in biological experiments (data not shown), it is not clear if the alternative strategy proposed here that works on the synthetic data generator would hold in the biological setting due to unexpected drug-drug interactions.

Furthermore, we were interested in looking at BP results when Assumption 2 is not at play. There are many assumptions in the BP method described in the main manuscript. One of the most dangerous assumptions is Assumption 2, which decouples the likelihood function from the dynamics of the model parameters. The synthetic data sets analyzed in the manuscript were generated without assumption 2. In those tests, we see good but imperfect inference of the underlying interactions. To isolate the consequences of assumption 2 and assess the performance of BP when assumption 2 is exact, we have generated this artificial data set (Figure S3A), which is not based on dynamic simulation of the generator network. Instead, we generated 300 training patterns for each model node separately as in Equation 5. We use random, uncorrelated values for the values on the right hand side of Equation 5 to generate values on the left hand side. The result is a set of low training patterns with low correlation (Figure S3B). Inferring network parameters with BP from this data produces near perfect inference with zero false positive and only 2 false negatives (Figure S3D). Although this type of data is infeasible in any biological system, it demonstrates that BP works almost perfect in the extreme case of ample, ideal data. This result also reinforces our suspicion that the effect of Assumption 2 is most likely responsible for the observed limits in BP performance.
