# Supplementary material for: Perturbation Biology: Inferring Signaling Networks in Cellular Systems
Source: PLoS Comput Biol. 2013 Dec 19;9(12):e1003290. doi: 10.1371/journal.pcbi.1003290 (PMC3868523; doi:10.1371/journal.pcbi.1003290)
Supplement: Text S4 — MCF7. This section examines BP performance on a previously published dataset of perturbation responses in breast cancer from the MCF7 cell line. This section also compares BP to our previously published method for inferring models based on a greedy Monte Carlo search. (DOCX) [file pcbi.1003290.s025.docx]

**Text-S4: BP results on MCF7 data set.**

We previously published a different inference algorithm, called CoPIA [[1](#_ENREF_1)], which was applied to a small data set collected on mcf7 breast cancer cell linesl. The data set consists of 9 measured protein and phenotype responses to

21 perturbation experiments involving 7 drugs. In brief, CoPIA consists of a nested search, in which the outer loop searches over network topologies, while the inner loop optimizes the parameters of that topology with a gradient descent algorithm. Both CoPIA and BP are based on the same model equation as defined in Equation 1. The likelihood function employed in the CoPIA method is a function of explicit numerical simulation, while the likelihood function in BP is based on an algebraic approximation of the steady state, as discussed in Assumption 2. In this short study, we applied our BP based pipeline on the same mcf7 data and compare the results. In this analysis, we used only a single beta (inverse-temperature) and lambda (sparsity) penalty. The BP results are based on the top 100 models from a set of 10,000 models generated from the BP-calculated marginal distributions. The CoPIA results were taken from the edges reported in Table 1 in [[1](#_ENREF_1)].

BP returns many of the significant interactions determined by CoPIA (Figure S10A), where the edge widths are proportional to the estimated posterior probability from the top 100 CoPIA models. Of all 23 interactions inferred by CoPIA 13 interactions are observed in at least 2 of the top 100 models sampled from BP-calculated marginal probability distributions. Five of the ten excluded interactions were not eligible for inference with BP (blue edges), since BP considers neither self-interactions nor edges directed into ‘activity nodes’. Thus only 5 interactions, only 2 of which were high probability edges in CoPIA, are excluded by BP.

BP infers 27 parameters (Figure S10B) with a probability of greater than 20%, of which 8 are also interactions in the top 100 CoPIA models (green). Edge width in the bottom figure is proportional to their frequency in the top 100 BP models. Of particular interest is the large number of high confidence incoming edges into MEK and pERK, both of which were inferred to have self-feedback interactions in the CoPIA models (11 between those two). It is also interesting that with the exception of an inhibitory interaction between pERK and MEK, there are no high confident interactions originating from either pERK or MEK. We do not yet understand why this happens, but we suspect there is a pathological connection between their high connectivity in BP and the inferred self-connectivity in the CoPIA models. Finally, the top 100 BP derived models have a mean squared error of roughly 0.1, which is as low as we’ve seen.

There is significant agreement among a subset of high confidence edges. This suggests that both BP and CoPIA methods have explored similar areas of the total solution space. However, BP does not return the exact same models as CoPIA. There are many potential reasons why the results are different. The first possibility is that BP’s restriction to exclude self-interaction and edges into activity nodes forces BP to search in an alternative region of solutions space. Secondly, CoPIA is based on a fairly simple MC topology search, which is unlikely to explore much of the full solution space. Consequently, the CoPIA results might well reflect a local-minima in the solution space. Regardless, the low mean-squared-error of the top 100 BP models is a positive result.

1. Nelander, S., et al., *Models from experiments: combinatorial drug perturbations of cancer cells.* Molecular systems biology, 2008. 4: p. 216.
