# Supplementary material for: Perturbation Biology: Inferring Signaling Networks in Cellular Systems
Source: PLoS Comput Biol. 2013 Dec 19;9(12):e1003290. doi: 10.1371/journal.pcbi.1003290 (PMC3868523; doi:10.1371/journal.pcbi.1003290)
Supplement: Text S5 — Gaussian graphical models. This section reports the results of modeling the melanoma data described in this manuscript with Gaussian Graphical Models; a popular and statistically rigorous method for discriminating between direct and indirect correlations between system variables. (DOCX) [file pcbi.1003290.s026.docx]

**Text-S5: Comparison with Gaussian Graphical Models**

Gaussian Graphical Modeling (GGM) is a well-developed model for describing couplings between random variables [[1](#_ENREF_1), [2](#_ENREF_2)]Correlation based networks are typically found to poorly distinguish between direct and indirect associations. Gaussian models are theoretically based on conditional dependencies in multivariate Gaussian distributions, and are likely to better distinguish between direct and indirect associations. Like correlations, couplings in GGMs are symmetric between model variables, and thus the couplings do not distinguish direction. We have adapted standard methods of calculating GGMs, based on inverting the covariance matrix, to infer couplings not only between model variables, but also between drugs and their targets (presuming the targets are included in the set of model variables), denoted *J* and *H* respectively. We use a standard maximum-likelihood estimator to calculate both J and H simultaneously. The goal of this brief study was to compare the strongest couplings from GGMs against those inferred by BP.

The results are summarized in this figure. The GGM calculation is dependent on a single parameter, referred to as the *weight cutoff*, which reweights the training patterns (in this case, perturbation experiments). The cutoff is constrained to be less than or equal to 1, and a cutoff of 1 corresponds to zero reweighting of the patterns. We find that a cutoff of 1 results in the highest likelihood model, given the data (Figure S11A) and the J and H matrices analyzed here are the result of this cutoff. The distributions for both J and H coupling strengths (Figure S11B and S11C, respectively) have the majority of strengths on or near zero, which indicates strong discerning power between strong and weak couplings.

To assess the extent of agreement between GGMs and the BP interactions we must decide where to fix the threshold, below which all J and H couplings are set to zero and thus ignored as edges in a network. If the threshold is too high, we end up with a very sparse network with only the strongest couplings. If the threshold is too low, all variables are coupled and we lose any ability to discern between strong and weak couplings. The agreement with varying threshold is summarized with the Receiver Operator Characteristic (ROC) curve in Figure S12 (top). Crucially, the curve lies firmly above the diagonal line. Two points on this curve that correspond to the maximum F1 score and Matthews Correlation Coefficient (MCC), are independent metrics of the balance between accuracy and precision and are marked on the ROC curve (red and green dots, respectively). Of particular interest is the threshold corresponding to the F1 max (J cutoff of 30), which appears to capture almost 70% of the BP interactions, with only a 40% false positive rate. The network of couplings above the F1-max threshold (Figure S12 bottom left) is still highly coupled with 122 couplings out of a possible 289, of which 18 are captured in the BP network (Figure S12 bottom right). None of the analyses presented here argue in favor of either methodology. The best strategy would depend on the method that can produce the best models in terms of reproducing response to trained perturbations and predicting response to new perturbations, none of which is evaluated here. Instead, we only have evidence that BP and GGMs are non-trivially capturing similar couplings between system variables.

It is important to realize that GGMs, however useful, are not sufficient for our modeling needs by themselves. For instance, they do not infer direction. Additionally, GGMs have no dynamic engine with which to run simulations of the models to predict response to perturbation. Despite these limitations, GGMs remain a well-regarded and mathematically grounded analysis of significant correlations. We are currently exploring ways of incorporating this GGM analysis into the BP-based strategy, potentially using GGMs to define reasonable initial conditions for all marginal distributions.

1. Krumsiek, J., et al., *Gaussian graphical modeling reconstructs pathway reactions from high-throughput metabolomics data.* BMC systems biology, 2011. **5**(1): p. 21.

2. Lauritzen, S., *Graphical Models*1996: Oxford University Press. 308.
