# Supplementary material for: Perturbation Biology: Inferring Signaling Networks in Cellular Systems
Source: PLoS Comput Biol. 2013 Dec 19;9(12):e1003290. doi: 10.1371/journal.pcbi.1003290 (PMC3868523; doi:10.1371/journal.pcbi.1003290)
Supplement: Text S6 — Implementation details. This section briefly explains some of the more tedious details regarding implementation of the BP algorithm described in this paper. (DOCX) [file pcbi.1003290.s027.docx]

S16: BP Implementation considerations.

The main manuscript covers all of the theoretical considerations of our BP algorithm. However, there are a few implementation issues that affect the efficiency of the algorithm.

Computational calculation of Equation 14.A for each update requires substantial memory and computational power. The algorithm has to keep track of all M x(N -1) factors, and then perform *M* multiplications, (*N-*1) times in preparation for a single update. We take advantage of the multiplicative relationship between a marginal P(wij ) and its factors $\rho^{\mu}(w_{ij})$, to reduce the number of computations need to evaluate $P^{\mu}(w_{ij})$and eliminate precision error associated with many multiplications of small numbers.

The marginal distribution and the global information are related by a simple division of the marginal by the cavity factor (Equations S1A and S1B). In log space, the same calculation is just a simple subtraction (Equation S1C).

**Equation S1**

$$\begin{matrix} P(w_{ij})=e^{-\lambda\delta(w_{ij})}\prod_{\nu}^{M} \rho^{\nu}(w_{ij}) \\ P^{\mu}(w_{ij})=\frac{P(w_{ij})}{\rho^{\mu}(w_{ij})} \\ \ln P^{\mu}(w_{ij})=\ln P(w_{ij})-\ln\rho^{\mu}(w_{ik}) \\ =-\lambda\delta(w_{ij})+ \sum_{\nu}^{M} \ln\rho^{\nu}(w_{ij})-\ln\rho^{\mu}(w_{ik}) \end{matrix}$$

The calculation in equation S1C is a fast calculation and involves only an inverse log operation, one subtraction and the cost of doing the normalization (K additions and K division operations). The calculation needs only to keep track of all factors and the sum of the natural logarithms of the factors across all M conditions, stored in an array $\Psi$.

**Equation S2**

$$\begin{matrix} \Psi(j)=\sum_{\nu}^{M} \ln\left(\rho^{\nu}(w_{ij})\right)-\lambda\delta(w_{ij}) \\ P^{\mu}(w_{ij})=\frac{1}{Z}\left(e^{\Psi(j)-\ln(\rho^{\mu}(w_{ik}))}\right) \end{matrix}$$

An additional advantage for performing the calculation in log space is that the operation does not risk precision errors from the multiplication of many small numbers. The calculation is a subtraction of negative numbers followed by a natural antilogarithm calculation, neither of which risk precision error. In non log space, the calculation requires M multiplications of probability distributions, which contain some very small numbers. In fact, an earlier implementation of the BP algorithm experienced detrimental errors for even small M. This error cascaded and produced meaningless results. The log space calculation is robust up to about at least M=1,000, above which has not been tested.

**Analytical approximation of the local factor update**

The integral in Equation 14B is not solvable analytically due to the nonlinearity introduced from the sigmoid function . One can solve the integral numerically with a method such as trapezoidal integration. However, since Equation 14B has to be calculated NxM times per iteration over many iterations, numerical solutions of this kind dramatically slow down the computational time to convergence. To alleviate the cost of this calculation, we use a simple algebraic rearrangement as shown in Equation S4B to make the fitness term $F^{\mu}(s_{k}^{\mu})$ linear with the integration variable $s_{k}^{\mu}$.

The goal is to rewrite the error inside the sum of squares term to be linear in $s_{k}^{\mu}$. The fitness is maximum when the cost is zero and therefore $x_{i}^{*\mu}=x_{i}^{\mu}$, which in turns means that . Inverting the nonlinear function on both sides of the equation (Equation S3A) and yields an approximation of the fitness that is linear with $s_{k}^{\mu}$.

**Equation S3**

Equation S4 is the analytical solution to the integral in Equation S3B.

**Equation S4**

**The Algorithm**

See Figure-S16 for an algorithm flow-chart.
