# Supplementary material for: Perturbation Biology: Inferring Signaling Networks in Cellular Systems
Source: PLoS Comput Biol. 2013 Dec 19;9(12):e1003290. doi: 10.1371/journal.pcbi.1003290 (PMC3868523; doi:10.1371/journal.pcbi.1003290)
Supplement: Text S7 — Comparison with Bayesian Inference. This section reports the results of a preliminary comparison of BP models against those inferred from Bayesian Inference. It also compares both class of models to random models and prior knowledge models. The data used in this preliminary study is from similar perturbation experiments on the dedifferentiated liposarcoma cell line DDLS8817. (DOCX) [file pcbi.1003290.s028.docx]

**BP comparison with Bayesian Network Models**

There are many methods available for constructing network models of signaling pathways from experimental data. These methods vary in their strengths and weaknesses with respect to scale (number of nodes), the ability to distinguish direct vs indirect correlations, causation, directed interaction and the ability to generate new dynamic or otherwise quantitative predictions to unseen drug perturbations. One of the most popular modeling and inference strategies, but by no means the best, is Bayesian Network Inference. We sought to compare our BP method for inferring interactions against the popular Bayesian Network Inference method. In this method, we use the academic software Biolearn1.0 available from the laboratory web page of Dr. Dana Pe’er. While other Bayesian network inference software exists, biolearn1.0 is the one that was part of one of the more popular papers for Bayesian Network inference of signaling pathways[[1](#_ENREF_1)]. The data, in this study, is a recently published dataset on a Dedifferentiated Liposarcoma cell line (DDLS8817). The data is also RPPA data of similar quality as that reported in this manuscript, and was generated from a similar design of systematic single drug and drug pair perturbations.

Given that Bayesian networks do not have any inherent dynamic simulation ability, we take interactions from Bayesian inference, incorporate them into the same neural-network like system of ODEs and optimize those parameters with the same gradient descent algorithm as applied to the BP models. For additional perspective, we also include both random models and models whose interactions are collected from prior knowledge. Random models restricted to having approximately the same number of total edges and the same ratio of positive to negative edges as observed in the BP models. Although all prior knowledge networks have the same network topology, we create diversity by exploring the space of parameter assignments. Only the top 100 of each model class are evaluated in this comparison.

All models, being parameterized neural-network like ODEs, are simulated under *in silico* conditions that mimic the perturbations in the training data. The reported metric is the mean-squared-error (MSE) per data point.

The distributions of the four classes of models (random, prior knowledge, Bayesian, and BP) are clearly separate (Supplemental Figure S-17). As expected, the random models have both the highest MSE and the widest distribution. The prior knowledge models perform better, with a distribution centered at an MSE of about 0.28. Fortunately, the Bayesian models, being the first class of models actually trained to data, show substantial improvement over the prior knowledge models as its distribution shows minimal overall with that from prior knowledge models. The Bayesian model distribution is centered approximately 0.25. BP models are clearly the best performing models, in this comparison, as their distribution is completely separated from all others with an average MSE centered at below 0.2, and thus about 20% improvement over Bayesian models. This analysis does not confirm that BP models are categorically better than Bayesian models or even Bayesian inference. Rather, it suggests that the out-of-the-box BP method outperforms the biolearn1.0 software for generating this kind of neural-network like simulation models of signaling pathways from this kind of data. Each interaction is not independently validated and thus neither method’s results were compared to any set of true biological interactions. Furthermore, the performance might vary with different classes of models. From this preliminary analysis, we believe that our method’s performance is at least on par with one popular modeling and inference strategy.

**Using BioLearn 1.0**. Details of the biolearn software are available from the lab website of Dr. Dana Pe’er. The software has many customizable user options controlling the scoring method and search method used in the algorithm. For this test, only the BDe scoring function was working. Attempts to use the Mean Squared Error scoring function failed. This failure is most likely results from insufficient replicates, since BioLearn was designed to analyze data from many single cells thus constituting a larger and more statistically significant sample. Furthermore, we saw best performance with the Greedy Hill Climbing search algorithm and an edge penalty of 0.0. Finally, BioLearn was run with a set of prior knowledge interactions. The prepared data and results files are also available in the supplement (Supplementary DataSet-S2). The code, however, is only available upon request through the laboratory webpage of Dr. Dana Pe’er (http://www.c2b2.columbia.edu/danapeerlab/html/software.html).

1. Sachs K, Perez O, Pe'er D, Lauffenburger DA, Nolan GP (2005) Causal protein-signaling networks derived from multiparameter single-cell data. Science 308: 523-529.
